# CRISPR/Cas9-based genome editing and functional analysis of *SlHyPRP1* and *SlDEA1* genes of *Solanum lycopersicum* L. in imparting genetic tolerance to multiple stress factors

**DOI:** 10.3389/fpls.2024.1304381

**Published:** 2024-02-02

**Authors:** Banashree Saikia, Remya S, Johni Debbarma, Jitendra Maharana, G. Narahari Sastry, Channakeshavaiah Chikkaputtaiah

**Affiliations:** ^1^ Biological Sciences and Technology Division, CSIR-North East Institute of Science and Technology (CSIR-NEIST), Jorhat, Assam, India; ^2^ Academy of Scientific and Innovative Research (AcSIR), Ghaziabad, Uttar Pradesh, India; ^3^ Distributed Information Centre (DIC), Department of Agricultural Biotechnology, Assam Agricultural University, Jorhat, Assam, India; ^4^ Advanced Computational and Data Science Division, CSIR-NEIST, Jorhat, Assam, India

**Keywords:** CRISPR/Cas9, multi-stress tolerance, drought, salinity, bacterial leaf spot, bacterial wilt, SlHyPRP1, SlDEA1

## Abstract

CRISPR/Cas is a breakthrough genome editing system because of its precision, target specificity, and efficiency. As a speed breeding system, it is more robust than the conventional breeding and biotechnological approaches for qualitative and quantitative trait improvement. Tomato (*Solanum lycopersicum* L.) is an economically important crop, but its yield and productivity have been severely impacted due to different abiotic and biotic stresses. The recently identified *SlHyPRP1* and *SlDEA1* are two potential negative regulatory genes in response to different abiotic (drought and salinity) and biotic stress (bacterial leaf spot and bacterial wilt) conditions in *S. lycopersicum* L. The present study aimed to evaluate the drought, salinity, bacterial leaf spot, and bacterial wilt tolerance response in *S. lycopersicum* L. crop through CRISPR/Cas9 genome editing of *SlHyPRP1* and *SlDEA1* and their functional analysis. The transient single- and dual-gene *SlHyPRP1* and *SlDEA1* CRISPR-edited plants were phenotypically better responsive to multiple stress factors taken under the study. The CRISPR-edited *SlHyPRP1* and *SlDEA1* plants showed a higher level of chlorophyll and proline content compared to wild-type (WT) plants under abiotic stress conditions. Reactive oxygen species accumulation and the cell death count per total area of leaves and roots under biotic stress were less in CRISPR-edited *SlHyPRP1* and *SlDEA1* plants compared to WT plants. The study reveals that the combined loss-of-function of *SlHyPRP1* along with *SlDEA1* is essential for imparting significant multi-stress tolerance (drought, salinity, bacterial leaf spot, and bacterial wilt) in *S. lycopersicum* L. The main feature of the study is the detailed genetic characterization of *SlDEA1*, a poorly studied 8CM family gene in multi-stress tolerance, through the CRISPR/Cas9 gene editing system. The study revealed the key negative regulatory role of *SlDEA1* that function together as an anchor gene with *SlHyPRP1* in imparting multi-stress tolerance in *S. lycopersicum* L. It was interesting that the present study also showed that transient CRISPR/Cas9 editing events of *SlHyPRP1* and *SlDEA1* genes were successfully replicated in stably generated parent-genome-edited line (GEd0) and genome-edited first-generation lines (GEd1) of *S. lycopersicum* L. With these upshots, the study’s key findings demonstrate outstanding value in developing sustainable multi-stress tolerance in *S. lycopersicum* L. and other crops to cope with climate change.

## 1 Introduction

The global temperature, fluctuation in weather patterns, sea level rise, increase in drought, and floods make the world’s population face expanding risks of food security, especially in underdeveloped and developing countries (Baillo et al., 2019; Zandalinas et al., 2021). The serious climate shift is giving rise to abiotic stresses like drought, salinity, heat, and cold. This, in turn, leads to an outburst of biotic stresses, including pests and pathogens, adversely affecting crop productivity and causing yield loss (Baillo et al., 2019; Hossain, 2019; Teshome et al., 2020). It expected that, by 2050, abiotic stresses such as drought, salinity, and temperature extremes will cause a 50% loss in average crop productivity (Brychkova et al., 2013; Calanca, 2017; Malhi et al., 2021). Crop improvement using conventional breeding methods, random mutagenesis, or genetic recombination is time-consuming and is not enough to meet the increasing global food demand (Abdelrahman et al., 2018; Ahmar et al., 2020). In that regard, precision speed breeding approaches like CRISPR/Cas9 can help to meet the needs for efficient crop development research (Pickar-Oliver & Gersbach, 2019; Saikia et al., 2020a; Saikia et al., 2020b). Even single-base editing with the help of the CRISPR/Cas9 approach through non-homologous end joining (NHEJ) has great potential to aid in the breeding of crops that can produce high yields under the conditions of abiotic/biotic stresses (Chen et al., 2019).

Apart from exploring it in various overexpression and knockout studies (Li et al., 2016; Yang et al., 2018), CRISPR/Cas9 technology has been successfully applied in the modification of tomato (*Solanum lycopersicum* L.) genomes for various traits including mitigation of single stress factors (Van Vu et al., 2020; Debbarma et al., 2023; Tran et al., 2023). However, considering the current climate change scenario and the occurrence of multiple stresses under field conditions, very few studies are ongoing in developing multi-stress tolerance in crops including tomato through advanced genome editing approaches. In addition, targeting single, dual, or multiple genes through CRISPR/Cas-based genome editing to develop multiple stress tolerance in crops has gained significant importance recently. In that context, targeting multi-stress negative regulatory genes is key to achieve multiple abiotic and biotic stress tolerance in crop plants. *Hybrid Proline-rich Protein* (*HyPRP1*) and *differentially expressed in response to arachidonic acid 1* (*DEA1*) are two potential multi-stress negative regulatory genes of biotic and abiotic stress responses in tomato. They are the members of 8 cysteine motif (8CM) family genes (Weyman et al., 2006a; Weyman et al., 2006b; Gehl et al., 2009; Gerszberg et al., 2015; Li et al., 2016; Saikia et al., 2020a; Saikia et al., 2020b). There are various reports where *HyPRP1* from *Solanaceous* model plants showed a significant role in the enhancement of pathogen susceptibility by suppressing the expression of defense-related genes (Huang et al., 2011; Yeom et al., 2012; Li et al., 2016; Yang et al., 2018). *HyPRP1* from *Gossypium barbadense* has been known to play a major role in the negative regulation of resistance to bacterial wilt via the thickening of cell walls and reactive oxygen species (ROS) accumulation (Yang et al., 2018). Through transgenic functional analysis and transcriptome analysis, it was shown very recently that *HyPRP1* acts as a negative regulator of various abiotic stresses in wild tomato *Solanum pennellii* as well as in other cultivated varieties (Li et al., 2016; Yang et al., 2018; Tran et al., 2021), whereas *DEA1* is a circadian-regulated gene in tomato that shares a sequence similarity to *Arabidopsis* cold-responsive gene *EARL1* (Kapoor et al., 2019). The transcription of *DEA1* was altered by *Phytophthora infestans* infection causing late blight in tomato. It has been reported that *GmDEA1* is involved in the biotic stress regulation of soybeans (Klink et al., 2011). The promoter region of *DEA1* contains stress-signaling elements (Weyman et al., 2006b). One such element is the W-box motif in tomato *DEA1* which is known to be associated with abiotic and biotic stress responses. Along with that, the W-box motif is also known to be associated with pathogen attacks (Eulgem et al., 2000). Salt stress tolerance response is known to be associated with an Alfin-1 response motif present in the promoter region of *DEA1* (Winicov & Bastola, 1999). Looking at the functional similarity, it is very prominent that *DEA1* has a lot to explore in the direction of multi-stress regulation. In our previous report, a protein–protein interaction study of *HyPRP1* and *DEA1* showed strong interaction signals when visualized under a confocal microscope (Saikia et al., 2020b).

To gain a deeper understanding of the functional role of *HyPRP1* and *DEA1* genes as negative regulators in imparting genetic tolerance to multiple stress factors in *S. lycopersicum* L. cv. Arka Vikas, we performed a systematic CRISPR/Cas9-based genome editing and functional analysis. We successfully generated target-specific CRISPR-editing events in transient and stable *S. lycopersicum* L. system (GEd0 and GEd1). The CRISPR/Cas9 genome editing of *SlHyPRP1* and *SlDEA1* revealed significant tolerance responses to multiple abiotic (drought and salinity) and biotic stress (bacterial wilt and bacterial leaf spot) diseases, respectively. Furthermore, with a detailed functional analysis of single and dual-gene CRISPR-edited system, we showed for the first time that *DEA1*, a poorly studied 8CM family gene, functions together as an anchor gene with *HyPRP1* in imparting multi-stress tolerance in *S. lycopersicum* L.

## 2 Materials and methods

### 2.1 SgRNA designing and CRISPR construct preparation through the Gibson assembly cloning method

**Figure 1:**
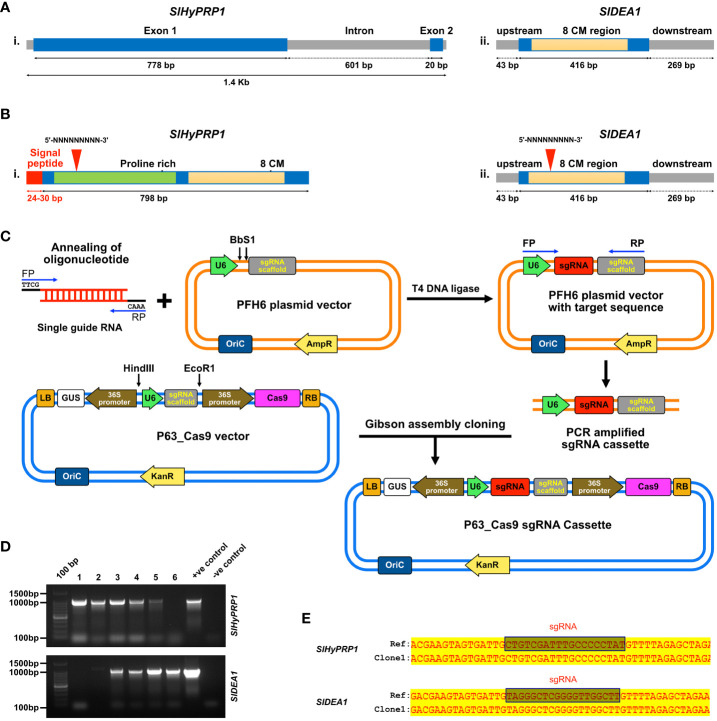
Diagrammatic description of the structure, functional motif, and sgRNA target sites of *SlHyPRP1* and *SlDEA1*, confirmation of CRISPR constructs and Gibson assembly cloning methodology. **(A)** Structure showing the genomic DNA region of *SlHyPRP1* (i) and *SlDEA1* (ii) along with the 8Cysteine motif region in light yellow. Proline-rich N-terminal region and signal peptide of *SlHyPRP1* are shown in green and red color, while the 8Cysteine motif is shown in light yellow. **(B)** sgRNA target sites for *SlHyPRP1* (i) and *SlDEA1* (ii) shown in red triangles (i and ii). **(C)** Stepwise methodology for Gibson assembly cloning method. **(D)** Screening of the positive CRISPR clones of *SlHyPRP1* and *SlDEA1* through colony PCR. **(E)** Confirmation of positive clones of *SlHyPRP1* and *SlDEA1* by Sanger sequencing.

The target 19–22-bp protospacer sgRNA of *SlHyPRP1* and *SlDEA1* genes was designed taking several parameters into account, such as target specificity, efficiency, fewer off-targets (not more than four mismatches), secondary structures, GC content (50%–55%) with the help of bioinformatics tools like CCTop (https://cctop.cos.uni-heidelberg.de:8043) (Stemmer et al., 2015), CRISPR-P (https://crispr.hzau.edu.cn/CRISPR2/) (Lei et al., 2014), and CHOPCHOP (https://chochop.ebu.uib.no/) (Labun et al., 2019) (**Supplementary Table S2**). Annealed oligonucleotides were generated in the PCR thermal cycler for which the designed 19–20 oligonucleotides along with primers having 4-bp overhang sites ATTG forward and AAAC reverse diluted into a final concentration of 10 µM, heated first at 98°C for 10 s and followed by slowly cooling down to 25°C. The 3,612-bp-long pEH6 vector (sgRNA entry vector) was digested and linearized with the *BbsI* enzyme (**Supplementary Table S1**). With the help of T4 DNA ligase, the annealed oligonucleotides were then inserted into the linear pFH6 vector in the location between the U6 promoter and the sgRNA scaffold. DH5α competent cells were used for transforming the ligated product, and they were grown for 2 h in liquid LB media. Then, the liquid LB medium carrying the transformed colonies was spread-plated on LB solid media supplemented with kanamycin (100 mg/L) to select positive colonies. A PCR amplicon size of around 416 bp was first amplified, which is the sgRNA cassette from the positive clone. Using a mini-elute Gel Extraction kit (Qiagen, Germany), the PCR product was extracted from the gel and purified. Those purified products were then Sanger-sequenced to confirm the sgRNA insertion. The Cas9 expression vector p63 was used to assemble the sgRNA entry clone into using it in the Gibson assembly method (Wang et al., 2015; Li et al., 2018). The clones were confirmed by colony PCR (**Figure 1D**).

### 2.2 Transient genetic transformation of *Solanum lycopersicum* L. through the infiltration method

The seeds of *S. lycopersicum* L. cultivar Arka Vikas were procured from ICAR-IIHR Bangalore, India. Following standard protocol, the seeds were stored for long-term use at 10°C and at 20% humidity in the seed storage cabinet (SR LAB, Mumbai). They were then germinated in soil rite in the ratio of 1:1:4 (vermiculite/perlite/cocopeat). To generate CRISPR-edited leaves (CRELs) in a transient system, the abaxial side of 16 leaves of 4- to 5-week-old tomato seedlings was syringe-infiltrated with an *Agrobacterium* suspension harboring CRISPR constructs for both *SlHyPRP1* and *SlDEA1* genes. Briefly, the agrobacterial cell suspensions were pressure-infiltrated to the abaxial surface of *S. lycopersicum* L. cv. Arka Vikas leaves using a 1-mL disposable syringe. Infiltration of the leaves was done in sections until the whole area appears translucent, and the leaves were saturated with agrobacterial cell suspensions. As it was infiltrated, the area of infiltration slowly became dark green (**Supplementary Figure S5**). After infiltration, the plants were covered with black polythene bags with holes in them, followed by spraying of water inside to maintain the high moisture content near the plants for 24 h. After that, the polythene bags were removed, followed by an occasional spray of water. Stress assays were then performed on the infiltrated and editing-confirmed leaves (**Supplementary Figure S5**) (Liu et al., 2019).

### 2.3 Stable transformation of *Solanum lycopersicum* L. through *Agrobacterium*-mediated transformation with binary CRISPR/Cas9 constructs

Arka Vikas cv. was stably transformed via Agrobacterium-mediated transformation using a modified protocol (Manamohan et al., 2011). For a stable transformation, the seeds were taken for surface sterilization. The sterilization was done for 5 min with 70% ethanol, 10 min washing with 4% sodium hypochlorite (V/V), and washing thrice with sterile distilled water. Then, ½ MS (Murashige and Skoog) media supplemented with 3% sucrose and 0.3% Gelrite (Sigma Aldrich, USA) was used to germinate the sterile seeds in the dark for 3 days. This was followed by growing the plants in a growth chamber at 25°C–28°C and 70% relative humidity with a light intensity of 15 µEm-2sec-2- and 16/8-hour light–dark photoperiod. The hypocotyl segment of the 10- to 12-day-old seedlings was transformed via an *Agrobacterium tumefaciens* (LBA4404) strain harboring the CRISPR/Cas9 binary constructs of *SlHyPRP1* and *SlDEA1*. The hypocotyl was washed with the antibiotic cefotaxime (250 mg/L) for 20 min to remove excessive *Agrobacterium* growth. After drying them on a sterile surface, the hypocotyls were covered and kept at 25°C in the dark for 2 days at 70% relative humidity. The hypocotyl segments were subcultured bi-weekly on shooting media containing MS media + sucrose and 1 mg/L of zeatin for 1 month. The shoots, upon attaining a size of approximately 1.5–2 cm, were transferred to a selective rooting medium. The rooting media containing MS media + sucrose at 1 mg/mL were supplemented with 0.1 mg/L indol 3-butyric acid. The well-rooted plants were transferred to soil rite (mixture of cocopeat/vermiculite/perlite/vermicompost) for acclimatization and kept in a greenhouse to study the replication of gene editing events (**Supplementary Figure S7**). Because our Cas9 plant expression vector did not have an antibiotic selection marker, for stably regenerated plants, we directly performed PCR-based molecular confirmation using Cas9 primers. The Cas9-positive transformant plants were regenerated through shooting and rooting media to generate stable CRISPR transformant plants of *S. lycopersicum* as mentioned above.

### 2.4 *Agrobacterium* cell culture and preparation of infiltration suspension

Single guide RNAs of *SlHyPRP1* and *SlDEA1* harboring in p63 CRISPR/Cas9 expression vector were introduced by electroporation method. For the transformation of CRISPR constructs, *SlHyPRP1* and *SlDEA1* (3 µL) were added in 100 µL of *Agrobacterium* LBA4404 cells. Electric shock has been employed, after which 1 mL of YEB media was immediately added in a 2-mm cuvette. The cells were incubated and allowed to grow at 29°C for 3 h at 220 RPM. Then, the cells were centrifuged for 1 min, and 200 µL of fresh YM media was added. The bacterial culture was plated in YM media containing yeast (0.4 g), mannitol (10.0 g), K_2_HPO_4_·3H_2_O (0.5 g), NaCl (0.1 g), MgSO_4_·7H_2_O (0.2 g), and bacteriological agar (15 g) in 1 L of double-distilled water at pH 7, following the Invitrogen Life Technologies user manual, and autoclaved at 121°C for 15 min (100 µg/mL kanamycin and 50 µg/mL streptomycin) for 2 days at 29°C. The positive clones were grown and confirmed by PCR analysis using U6 forward and sgRNA scaffold reverse primers and Cas9 primers. *SlHyPRP1* and *SlDEA1* were co-transformed for dual-gene CRISPR/Cas9 *SlHyPRP1*_*SlDEA1* editing, for which properly grown cultures were inoculated into YM suspension medium in separate flasks and grown at the same temperature in a shaker incubator at 180 RPM overnight. The YM medium contains yeast extract (0.4 g/L), mannitol (10 g/L), K_2_HPO_4_·3H_2_O (0.5 g/L), NaCl (0.1 g/L), and MgSO_4·_7H_2_O (0.2 g/L), with 100 µg/mL kanamycin and 50 µg/mL streptomycin, and the pH was maintained at 7. The strains were allowed to grow until the cell density reached an OD_600_ of 0.8–1. Each 20 mL of bacterial culture was centrifuged at 5,000–6,000 RPM for 20 min at 25°C, and the supernatant was discarded. The cells were resuspended with the infiltration medium containing 10 mM MgCl_2_ and 10 mM MES at pH 5.6. The flasks containing the infection medium of *SlHyPRP1* and *SlDEA1* were mixed, and the resuspension was incubated for 30 min at 29°C and was then ready for infiltration.

### 2.5 Infiltration of the *Solanum lycopersicum* L. cv. Arka Vikas leaves

Seedlings at 4 weeks old were chosen to infiltrate (4-week-old plants were chosen because the leaves of *S. lycopersicum* L. were relatively young and fully expanded after growing for about 3 weeks, which are essential for the transient expression in *S. lycopersicum* L.). The agrobacterial cell suspensions were pressure-infiltrated to the abaxial surface of *S. lycopersicum* L. cv. Arka Vikas leaves using a 1-mL disposable syringe (**Supplementary Figure S5**). Infiltration of the leaves was done in sections until the whole area appeared translucent, and the leaves were saturated with agrobacterial cell suspensions. As it was infiltrated, the area of infiltration slowly became dark green. After infiltration, the plants were covered with black polythene bags with holes in them, followed by spraying of water inside to maintain the high moisture content near the plants for 24 h. After that, the polythene bags were removed, followed by an occasional spray of water (**Supplementary Figure S6**).

### 2.6 β-Glucuronidase histochemical staining

**Figure 2:**
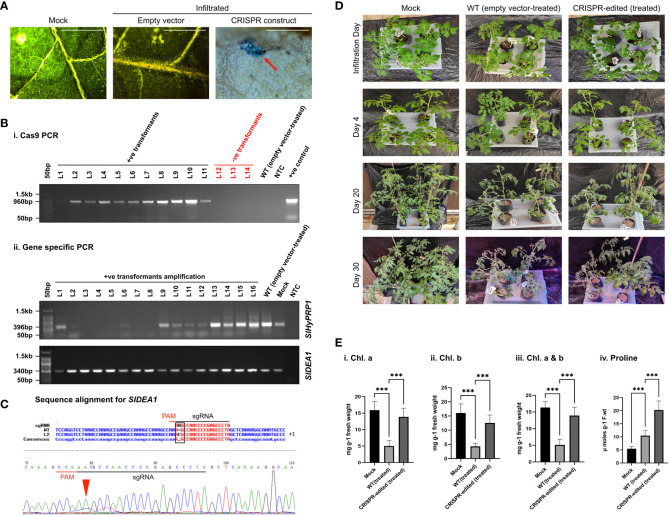
Drought stress response study of CRISPR transformants of *SlHyPRP1* **and** *SlDEA1.* **(A)** GUS and molecular confirmation of CRISPR transformants of *SlHyPRP1* and *SlDEA1* leaves after GUS staining and observation under a compound microscope. The bar represents the image taken in 500 pixels under a compound microscope. **(B)** Primary confirmation of dual-gene CRISPR transformants through PCR using Cas9 primers (i) followed by PCR using gene-specific primers of *SlHyPRP1* and *DEA1* with positive and negative controls (ii). **(C)** The Sanger sequencing alignment of the gel-purified products of *SlDEA1* gene showed a single-base-pair insertion mutation that caused editing within the gene sequence. The alignment was done using Multalin online tool. **(D)** Plain view of transiently transformed *S. lycopersicum* L. plants grown in soil under drought treatment. Severely visible drought effects on the 30th day were observed on the WT (empty vector-treated) seedlings (last panel, wilting of the whole seedling) as compared to dual-gene CRISPR-edited (treated) (right panel) seedlings. **(E)** The effect of drought stress on chlorophyll content (mg g^-1^ fresh weight) and proline (µmol) in transiently transformed plants. Chl.a, Chl.b, and total Chl. (a and b) showing elevated levels in CRISPR-edited (treated) plants treated with drought compared to WT (empty vector-treated), while the untreated mock showed a constant level of Chl. content (i–iii). The proline content was significantly higher in CRISPR-edited (treated) plants compared to WT (empty vector-treated). The untreated mock showed a steady level of proline (iv). Data expressed as mg/g of fresh weight are the mean ± SE of three biological replicates. The mean ± SE values from technical replicates (three) are represented in the error bar (*P\0.05, **P\0.01, ***P\0.001 according to the Tukey test, followed by Student’s *t*-test).

**Figure 3:**
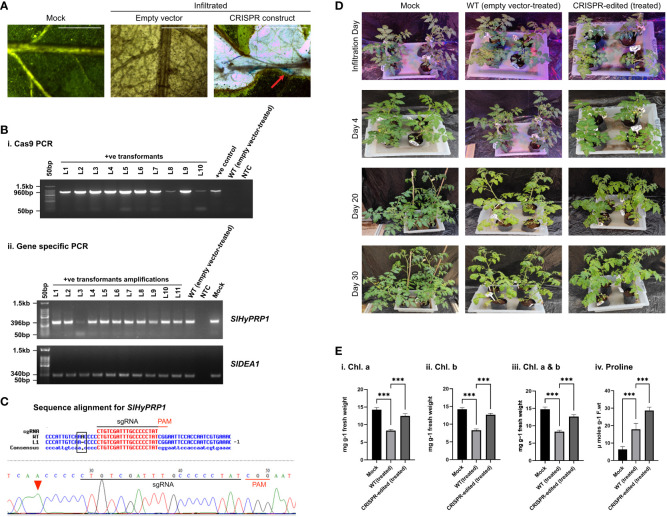
Salt stress response study of CRISPR transformants of *SlHyPRP1* and *SlDEA1.* **(A)** GUS and molecular confirmation of CRISPR transformants of *SlHyPRP1* and *SlDEA1* leaves after GUS staining and as observed under a compound microscope. The bar represents the image taken in 500 pixels under a compound microscope. **(B)** Primary confirmation of dual-gene CRISPR transformants through PCR using Cas9 primers (i) followed by PCR using gene-specific primers of *SlHyPRP1* and *SlDEA1* with positive and negative controls (ii). **(C)** Sanger sequencing alignment of the gel-purified products of *SlHyPRP1* gene showing a single-base-pair deletion mutation that caused editing within the gene sequence. The alignment was done using Multalin online tool. **(D)** Plain view of transiently transformed *S. lycopersicum* L. plants grown in soil under salt treatment. Visibly severe salt effects (yellowing and crunchy texture) on the 30th day were observed on the WT (empty vector-treated) seedlings (last panel, wilting of the whole seedling) as compared to dual-gene CRISPR-treated (right panel) seedlings. **(E)** The effect of salt stress on chlorophyll content (mg g^-1^ fresh weight) and proline (µmol) in transiently transformed plants. Chl.a, Chl.b, and total Chl. (a and b) showing elevated levels in CRISPR-edited (treated) plants treated with drought compared to WT (empty vector-treated), while the untreated mock showed a constant level of Chl. content (i–iii). The proline content was significantly higher in CRISPR-edited (treated) plants compared to WT (empty vector-treated). The untreated mock showed a steady level of proline (iv). Data expressed as mg/g of fresh weight are the mean ± SE values of three biological replicates. Mean ± SE from technical replicates (three) are represented in the error bar (*P\0.05, **P\0.01, ***P\0.001) according to the Tukey test, followed by Student’s *t*-test.

**Figure 4:**
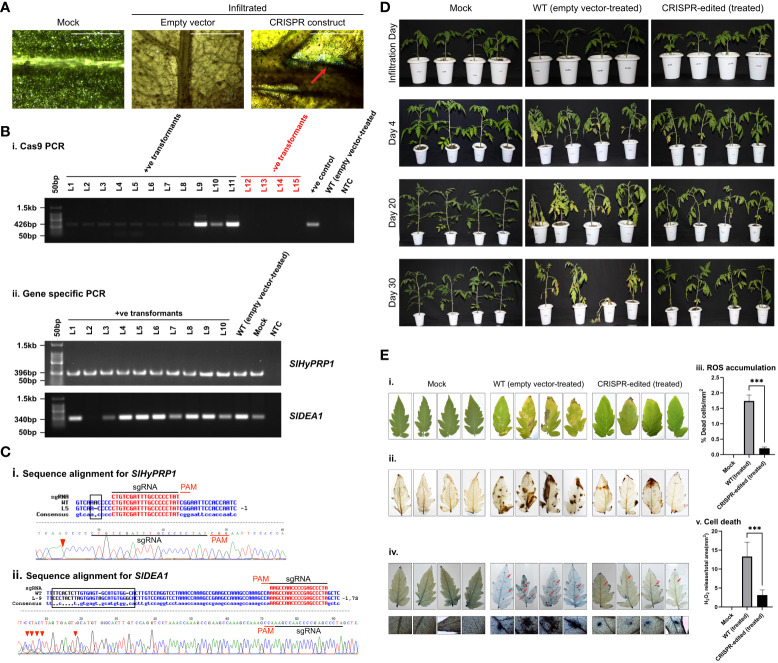
Bacterial leaf spot stress response study of CRISPR transformants of *SlHyPRP1* and *SlDEA1.* **(A)** GUS and molecular confirmation of CRISPR transformants of *SlHyPRP1* and *SlDEA1* leaves after GUS staining and as observed under a compound microscope. The bar represents the image taken in 500 pixels under a compound microscope. **(B)** Primary confirmation of dual-gene CRISPR transformants through PCR using Cas9 primers (i) followed by PCR using gene-specific primers of *SlHyPRP1* and *DEA1* with positive and negative controls (ii). **(C)** Sanger sequencing alignment of the gel-purified products of S1HyPRP1 (i) and *S1DEA1* (ii) genes showing base substitution and deletion. The alignment was done using Multalin. **(D)** Phenotypic analysis of transiently transformed *S. lycopersicum* L. seedlings grown and exposed to *X. campestris* pv. *vesicatoria*. The visible symptoms were observed on the WT (empty vector-treated) seedlings (middle panel, starting with small brown spots on the leaves followed by chlorosis, necrosis, and eventual plant death) as compared to dual-gene CRISPR-edited (treated) (last panel) seedlings. **(E)** Visualization (photographic) of *X. campestris* pv. *vesicatoria*-infected 1-month-old leaves of WT (empty vector-treated) and CRISPR-edited (treated) plants stained with DAB (i and ii). Quantification of H_2_O_2_ release (ROS accumulation)/total area (mm^2^) was measured with Image J software (iii). Microscopic visualization of *X. campestris* pv. *vesicatoria.* One-month-old leaves of WT (empty vector-treated) and CRISPR-edited (treated) plants stained with trypan blue (iv). The bar represents 0.2 µm. Quantification of cell death/total area (mm^2^) was measured with Image J software (v). Data were collected from three independent biological replicates; four leaves were used in each biological replicate. The average lesion areas are expressed as means ± standard errors (*n* = 3). Mean ± SE from technical replicates (three) are represented in the error bar (*P\0.05, **P\0.01, ***P\0.001) according to the two-way ANOVA test and Tukey’s test, followed by Student’s *t*-test.

**Figure 5:**
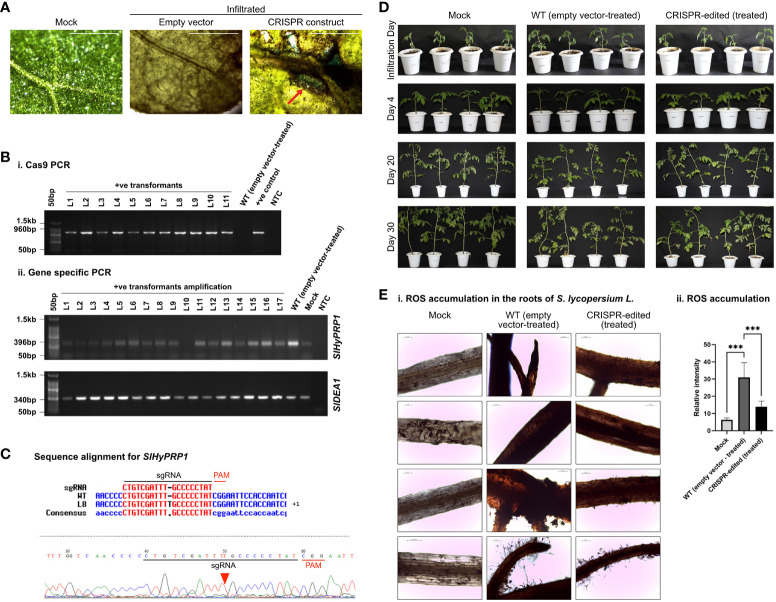
Bacterial wilt stress response study of CRISPR transformants of *SlHyPRP1* **and** *SlDEA1*. **(A)** GUS and molecular confirmation of CRISPR transformants of *SlHyPRP1* and *SlDEA1* leaves after GUS staining and as observed under a compound microscope. The bar represents the image taken in 500 pixels under a compound microscope. **(B)** Primary confirmation of dual-gene CRISPR transformants through PCR using Cas9 primers (i) followed by PCR using gene-specific primers of *SlHyPRP1* and *SlDEA1* with positive and negative controls (ii). **(C)** Sanger sequencing alignment of the gel-purified products of the *SlHyPRP1* gene showing base substitution and deletion that led to the ORF shift mutation causing editing events. The alignment was done using Multalin tool. **(D)** Phenotypic analysis of transiently transformed *S. lycopersicum* L. seedlings grown and exposed to *R. solanacearum*. No visible symptoms were observed on the WT (empty vector-treated) seedlings as well as dual-gene CRISPR-edited (treated) seedlings. **(E)** Representative microscopic images of DAB staining. Visibly higher necrosis was observed on the roots of WT (empty vector-treated) seedlings as compared to dual-gene CRISPR-edited (treated) seedlings (i). The bar represents 0.2 µm. Quantification of fluorescence and coloration intensity was performed using ImageJ (ii). The level of significance difference (*P\ 0.05, **P\0.01, ***P \0.001) was calculated by two ANOVA tests using PRISM Graph Pad 9.0. software.

*S. lycopersicum* L. leaves were collected 3–7 days post-agroinfiltration of dual-gene CRISPR constructs of *SlHyPRP1* and *SlDEA1* for histochemical β-glucuronidase (GUS) staining. Histochemical GUS staining was carried out as described (Liu et al., 2019). The infiltrated leaves were submerged in the GUS staining solution containing 50 Mm NaH_2_ PO_4_, 2 Mm K_3_Fe (CN), 1 mM X-Gluc, and 1% Triton X. The volume was made up to 20 mL and incubated for 16 h in the dark at 37°C. The GUS-stained leaves were observed under a light microscope and photographed. The wild-type (WT) leaves stained with GUS were used as negative controls. The stained leaves were photographed (**Figures 2A**, **3A**, **4A**, **5A**).

### 2.7 Genomic DNA preparation

The plant tissues (approx. 100 mg) of infiltrated and stably transformed lines were crushed in 1 mL DNA extraction buffer with a ceramic mortar and pestle. The homogenized sample was centrifuged for 10 min at 12,000 RPM. The supernatant was collected carefully, and an equal volume of phenol/chloroform/isoamyl alcohol (25:24:1) was added, and the mixture was centrifuged for 10 min at 12,000 RPM. The aqueous phase was separated, an equal volume of chloroform and isoamyl alcohol (24:1) was added and centrifuged for 10 min, and the aqueous phase was collected. Furthermore, 100% isopropanol was added and incubated at room temperature for 1 h. The DNA was pelleted down by centrifuging at 15,000 RPM for 15 min. The pellet was washed with 70% ethanol for 10 min. After the removal of ethanol, the pellets were diluted with TE buffer (Tris, EDTA, pH 8). Then, 1 µL of RNaseA (25 mg/mL) was added and incubated at 37°C for 20 min. The concentration of gDNA samples was quantified using a nanodrop spectrophotometer (Eppendorf Bio-spectrometer). The genomic DNA was stored at -20°C in a freezer.

### 2.8 Screening and validating editing events by Sanger sequencing

Genomic DNA was isolated from 3–7 days post-agroinfiltration of dual-gene CRISPR constructs of *SlHyPRP1* and *SlDEA1*, and for each sample gDNA was isolated from 2-week-old leaves using the SDS method (Xia et al., 2019). Using Cas9-specific primers, the positive CRISPR transformants of *SlHyPRP1* and *SlDEA1* were molecularly confirmed by PCR using Emerald Amp® GT PCR Master Mix (DSS-Takara) (**Supplementary Table S1**). For the negative control, genomic DNA isolated from WT untransformed leaves was taken. PCR amplification of dual-gene (*SlHyPRP1* and *SlDEA1*) transformant plants was performed using gene-specific primers of both genes separately with Emerald Amp® GT PCR Master Mix (DSS-Takara) (**Figures 2B**, **3B**, **4B**, **5B**; **Supplementary Table S1**). The PCR-purified products were then Sanger-sequenced. The sequencing reads were aligned with the reference gene (WT) sequence, which revealed the CRISPR editing events (INDELs, large/small deletions, etc.). The analysis was done with the help of Vector NTI software (Thermo Fisher, Life Technologies) and the Multalin online sequence alignment tool (**Figures 2C**, **3C**, **4C**, **5C**; **Supplementary Figures S8**, **S9**).

### 2.9 Identification of off-target loci

The putative off-targets of *SlHyPRP1* and *SlDEA1* genes were analyzed using CCTop and CRISPR P 2.0 version software tool (Stemmer et al., 2015). The oligos of putative off-target genes were designed and performed with off-target analysis (**Supplementary Tables S1**, **S3**).

### 2.10 Stress assays on transiently transformed CRISPR-edited lines

#### 2.10.1 Drought stress

The selected plants were subjected to transient drought stress analysis (Liu et al., 2019) with slight modifications. The plants were grown in soil with sufficient watering, and the leaves were infiltrated with *A. tumefaciens* containing dual-gene CRISPR constructs of *SlHyPRP1* and *SlDEA1* and maintained for 7 days During this time, the GUS and molecular confirmation for CRISPR-positive transformants have been carried out as described earlier. At 7 days post-infiltration, the CRISPR-positive transformants were subjected to drought treatment by halting the irrigation. The plants infiltrated with empty plasmid p63:CMV : Cas9:beta glucuronidase (dicot) were used as controls. The irrigated plants throughout the assay were used as mock. The plants were observed for morphological and phenotypic characteristics of drought stress response in CRISPR-edited treatment, WT (empty vector-treated), and mock on a weekly basis for up to 1 month and photographed. When the plants showed obvious wilting, yellowing, and lethal effects of dehydration, one set of leaves was collected for evaluation of CRISPR editing events, and another set of leaves was collected for physiological and biochemical assays of drought stress response. The experiment was carried out with three biological replicates (**Supplementary Figure S1**; **Supplementary Tables S4**, **S5**).

#### 2.10.2 Salinity stress

At 3 days post-agroinfiltration, the selected plants were subjected to salt stress as described with minor modifications (Liu et al., 2019). The GUS and PCR confirmation of CRISPR-positive transformants has been confirmed similar to drought stress as described earlier. The plants were irrigated with 250 mmol/L NaCl solution, and about 2 to 3 L NaCl solution was poured into the trays containing tomato plants in plastic bags. The holes were made in the plastic bags to allow NaCl to enter the plants through these holes. The excess NaCl solution was poured out after 24 h of soaking. The plants were treated with NaCl solution every 4 to 5 days until the plants showed apparent phenotypes. The plants infiltrated with empty plasmid p63:CMV : Cas9:beta glucuronidase (dicot) were used as controls. The untreated plants were used as mock throughout the assay. One set of leaves was collected for the evaluation of CRISPR editing events, and another set of leaves was collected for the physiological and biochemical assays of salt stress response. The experiment was carried out with three biological replicates (**Supplementary Figure S2**; **Supplementary Tables S6**, **S7**).

#### 2.10.3 Bacterial leaf spot stress

For bacterial stress assays, 4-week-old Agro-infiltrated *S. lycopersicum* L. plants were selected and inoculated with *Xanthomonas campestris* pv. Vesicatoria which is the causal organism of the bacterial spot disease of *S. lycopersicum* L. The master culture of *Xanthomonas campestris* pv. Vesicatoria BU::0001 was procured from ITCC, New Delhi. The master plate was subcultured into YGCA solid media (yeast extract, glucose, CaCO_3_ finely granulated, and bacteriological agar) at 28°C for 2 to 3 days (ITCC, New Delhi; prescribed selective media). The inoculation of plants with *X. campestris* pv. Vesicatoria and the assessment of disease progression were carried out by dipping the undetached leaves of 4-week-old Agro-infiltrated *S. lycopersicum* L. plants into bacterial suspensions having OD_600_ in the range of 0.5 to 0.8 and containing yeast extract, glucose, and finely granulated CaCO_3_ grown for 24 h (Tamir-Ariel et al., 2007). Before dipping into the bacterial suspension, the abaxial side of undetached leaves was pricked with a needle to ease the passage of the bacterial culture (Hoshikawa et al., 2019). The excess of the bacterial suspension was washed off with distilled water twice. The plants were covered with plastic bags to keep them in a moist environment for 48 h. The plants infiltrated with empty plasmid p63:CMV : Cas9:beta glucuronidase (dicot) were used as controls. The untreated plants were used as mock throughout the assay. After the removal of the plastic bags, disease progression and development of symptoms were observed and photographed until 1 month. One set of leaves was collected for the evaluation of CRISPR editing events and another set of leaves was collected for ROS accumulation and histochemical cell death quantification by staining the leaves with 3,3′-diaminobenzidine (DAB) and trypan blue (TB), respectively (Bach-Pages and Preston, 2018; Yang et al., 2018). The experiment was carried out with three biological replicates (**Supplementary Figure S3**; **Supplementary Tables S8**, **S9**).

#### 2.10.4 Bacterial wilt stress

Agro-infiltrated *S. lycopersicum* L. plants at 4 weeks old were selected and inoculated with *Ralstonia solanacearum* which is the causal organism of the bacterial wilt disease of *S. lycopersicum* L. The master culture of the virulent strains was procured from ITCC, New Delhi. The master plate was subcultured into triphenyl tetrazolium chloride (TTC) media (peptone, casein hydrolysate, glucose, triphenyl tetrazolium chloride—0.005%, and bacteriological agar at pH 7.2) at 29°C for 2 to 3 days. The virulent colonies were further inoculated and cultured on TTC suspension (peptone, casein hydrolysate, glucose, triphenyl tetrazolium chloride—0.005%) at 29°C for 24 h (Kumar et al., 2018). The concentration of the suspension was adjusted to 0.8–1 by taking the OD at 600 nm. The inoculation of plants and the assessment of disease progression were carried out by using the protocol described by Kim et al. (2016) with slight modifications. The roots of the *S. lycopersicum* L. plants were artificially wounded using a sterile blade, and the wounded roots were then dipped into the bacterial suspension until they became saturated, while a few roots were still in the soil so as not to disrupt the natural growth of the plants. The locations of the wounds were marked after removing them from the bacterial suspension and were covered with soil. The plants infiltrated with empty plasmid p63:CMV : Cas9:beta glucaronidase (dicot) were used as controls. The untreated plants were used as mock throughout the assay. The disease progression and the development of symptoms were observed and photographed up to 1 month. One set of leaves was collected for the evaluation of CRISPR editing events, and roots were collected for ROS (hydrogen peroxide) accumulation study by staining them with DAB (Bach-Pages and Preston, 2018; Yang et al., 2018). However, no observable symptoms on the aerial part of the plants could be recorded. The experiment was carried out with three biological replicates (**Supplementary Figure S4**; **Supplementary Table S10**).

#### 2.10.5 Determination of chlorophyll content

The determination of chlorophyll content was carried out by following the equations described with little modifications (Arnon, 1949; Sibley et al., 1996). About 0.1 g of leaf was ground in a pre-chilled pestle and mortar with 4 mL of 90% acetone until fully homogenized. The homogenized samples were centrifuged at 10,000 RPM for 10 min. The optical density of the supernatant was measured at 664 and 647 nm, respectively, using a spectrophotometer (The Eppendorf BioPhotometer D30). The content of chlorophyll a and chlorophyll b was calculated by the following formula:


$$\left[\text{Chlorophylla}\right]=12.4\times A_{664}-2.79\times A_{647}$$

$$\left[\text{Chlorophyllb}\right]=20.7\times A_{647}-4.67\times A_{664}$$

$$\left[\text{Chlorophyllab}\right]=17.90\times A_{647}+8.08\times A_{664}$$

The experiments were carried out with three biological replicates (**Supplementary Tables S4**, **S6**).

#### 2.10.6 Estimation of proline content

Following the methods described by Bates et al. (1973), the proline content was measured. A standard curve was generated with the serial concentration of proline made in 3% sulphosalicylic acid as follows: 50 µM, 100 µM, 150 µM, 200 µM, 300 µM, and 1 mL for each dilution. Furthermore, 500 µL of acetic acid and 500 µL of ninhydrin reagents were added to each 500 µL standard solution in 15 mL falcons, boiled in a water bath for 45 min, and then cooled in ice for 30 min. To each sample, an equal volume of toluene was added; the sample was vibrated for 1 min followed by centrifuging at 1,000 RPM for 5 min. The optical density was measured at 520 nm by using a spectrophotometer. Each 0.5 gm of the sample was ground in 2 mL 3% sulphosalicylic acid, and the fully homogenized samples were centrifuged at 500 RPM for around 5 min and then the supernatant was collected. To each of the 500-µL supernatant collected in a falcon tube, 500 µL ninhydrin was added in the dark. The falcons containing the solution were boiled in the water bath for 45 min and cooled in ice for 30 min. The solutions change their color from light pink to violet depending on the presence of proline content. After cooling down the samples on ice, an equal volume of toluene was added to each sample, followed by vibrating for 1 min and then centrifugation at 1,000 RPM for 5 min. The upper phase of the samples was taken to a spectrophotometer (The Eppendorf Bio Photometer D30) to measure their optical density at 520, nm and the proline content was measured using the standard curve of the above-mentioned concentrations. The experiments were carried out with three biological replicates (**Supplementary Tables S5**, **S7**).

#### 2.10.7 Hydrogen peroxide detection assay

For the hydrogen peroxide detection assay, the DAB staining method was used (Liu et al., 2017; Bach-Pages and Preston, 2018) with little modifications. The DAB (HiMedia) solution was prepared at a concentration of 1 mg/mL using 0.1 HCl; pH was adjusted to 3.6. To dissolve the DAB solution, it was kept at 37°C in vigorous shaking for 2 h. Mock, WT (empty vector-treated), and CRISPR-edited (treated) infected leaves were incubated overnight by dipping in 10 mL DAB solution at 37°C. The excess DAB solution was removed with distilled water. After that, a fixation solution was used to remove the chlorophyll content of the leaves, and these were photographed. The presence of H_2_O_2_ production/mm^2^ in WT (empty vector-treated) and the necrotic leaves of CRISPR-edited leaves were quantified using Image J software (Schneider et al., 2012). The level of significance (*P\0.05, **P\0.01, ***P\0.001) was calculated by pairwise Student’s *t*-test by using the PRISM GraphPad 9.0 software. Similar steps have been followed in terms of DAB staining of *S. lycopersicum* L. seedling roots to measure the intensity of ROS accumulation. The experiment was repeated thrice (**Supplementary Tables S8**, **S10**).

#### 2.10.8 Trypan blue assay to count cell death per total area of leaves

Trypan blue stain was used to detect the dead cells as described by Wang et al. (2011) and Bartsch et al. (2006) with slight modifications. To prepare trypan blue (HiMedia) stock solution, 10 mg of trypan blue was dissolved in 10 mL of distilled water along with 10 mL phenol and 10 mL of 85% lactic acid. Then, 95% ethanol at a ratio of 1:1 was used to dilute the stock solution to yield a workable solution. In the working solution, the leaves were dipped and incubated for 1 h; after that, they were boiled for 1 min, cooled, and stored at RT overnight (Bartsch et al., 2006; Liu et al., 2016). The chlorophyll content was removed by dipping the leaves in 95% ethanol and boiling them for 8 min (Rao et al., 2020). Then, the leaves were photographed and examined under a 4×/0.25 numerical aperture objective under bright-field microscopy (Leica microsystem, Germany) (Bartsch et al., 2006). The cell death per square millimeter was marked and quantified using Image J software (Schneider et al., 2012). The level of significance (*P\0.05, **P\0.01, ***P\0.001) was calculated by pairwise Student’s *t*-test using the PRISM GraphPad 9.0 software. The experiment was repeated thrice (**Supplementary Table S9**).

## 3 Results

### 3.1 *In silico* study of the structure and functional domains of *HyPRP1* and *DEA1*

By doing extensive literature mining *Hybrid proline-rich protein 1* (*HyPRP1)* and *Differentially expressed in response to Arachidonic acid-induced protein 1 SlDEA1*, members of 8 cysteine motif family genes have been chosen for the current study. The key reason for choosing these genes is that they are prominent in multi-stress responses in several plant species. In *Solanaceous* plants, they have been identified to be negative regulators of multiple biotic and abiotic stresses (Yeom et al., 2012; Li et al., 2016; Yang et al., 2018). The genomic information has been retrieved from Solgenomics and NCBI: the *SlHyPRP1* gene (gene name: Solyc12009650.1, NCBI accession no.: AF308937) and the *SlDEA1* gene (gene name: Solyc08g078900.1, NCBI accession no.: AF308937) (Saikia et al., 2020b). The structural arrangement that has been deduced for the genes is diagrammatically the N-terminal region of the *SlHyPRP1* CDS region that has a proline-rich domain, and the C-terminal region has 8CM (**Figure 1A**i, ii). A 24- to 30-bp-long signal peptide was found in the upstream of the *SlHyPRP1* N-terminal end (**Figure 1A**ii), whereas *SlDEA1* (416 bp) was found to be flanked 43 bp upstream and downstream by 269 bp along with the CDS containing C-terminal 8CM (**Figure 1A**iii). The comparative multiple sequence alignments were done for both *SlHyPRP1* and *SlDEA1* using the DNAMAN software tool where 8CM was found to be conserved in many *Solanaceous* species. This is the distinguishing feature of *SlHyPRP1* and *SlDEA1* proteins. The amino acid sequence alignment showed that the N-terminal proline-rich domain, as well as C-terminal 8CM, is uniformly present across a range of solanaceous species. These characteristic domains signify that *SlHyPRP1* and *SlDEA1* belong to the 8CM family proteins (Weyman et al., 2006a; Weyman et al., 2006b; Kapoor et al., 2019). There was a 96% similarity found between wild tomato *Solanum pennellii* and *Solanum lycopersicum* L. via phylogenetic analysis, whereas in the case of *SlDEA1*, 100% similarity was found with proline-rich protein from *S. pennellii* along with proline-rich protein from *S. tuberosum* with 98% similarity. Moreover, 91% similarity was shown by *EARL1* from *Capsicum annum* and *Capsicum chinensis* with *SlDEA1*. An analysis of the phylogenetic and multiple sequence alignment of both *SlHyPRP1* and *SlDEA1* show that 8CM was conserved between Solanaceous plants (Weyman et al., 2006a; Weyman et al., 2006b; Li et al., 2016). Significantly, *SlDEA1* and *Arabidopsis* lipid transfer protein EARL1 share 50–65% similarity, which is known to be associated with multiple abiotic stress tolerance in plants (Weyman et al., 2006a; Weyman et al., 2006b; Saikia et al., 2020a).

### 3.2 CRISPR constructs of *SlHyPRP1* and *SlDEA1* in a dual-gene editing system to obtain precise transient and stable editing

The sgRNA for *SlHyPRP1* was designed at the N-terminal proline-rich domain and for *SlDEA1* sgRNA was designed at the N-terminal end as well (**Figure 1B**). The CRISPR constructs were prepared using the Gibson assembly cloning method (**Figure 1C**). The tomato plants were transformed both transiently and stably to obtain CRISPR/Cas9-mediated editing events. For transient transformation, the abaxial side of the leaves of the tomato plants was infiltrated with the help of a syringe, whereas for stable transformation an *Agrobacterium-*mediated plant transformation was performed by taking the tomato explants (hypocotyl and cotyledon) (**Supplementary Figure S5**). The transiently transformed lines Sanger sequencing analysis for CRISPR editing events showed a single base insertion mutation of *SlDEA1* in the drought-stressed transient lines (**Figure 2C**). The editing was found to be within the sgRNA target region. Along with that, clear editing in the upstream and downstream of the PAM site was observed in several transient leaves (**Supplementary Figure S8**), whereas in the case of salt-stressed lines the editing was observed in the N terminal region close to the sgRNA (**Figure 3C**). The other plants showing editing events were also found to be at or near the sgRNA target site (**Supplementary Figure S8**). A number of leaves showed editing which showed positive response towards abiotic stress. This was a significant finding of CRISPR editing events which has been observed from the transient assay in *S. lycopersicum* L.

Similarly, in the transient plants observed under biotic stress, a mixed pattern of editing was observed in the form of single and large deletions as well as insertions and substitution mutations in several leaves (**Supplementary Figure S8**). Interestingly, under bacterial spot stress, editing could be obtained in both genes *SlHyPRP1* and *SlDEA1*. Prominent editing events were found nearby and upstream of the PAM site in the case of *SlHyPRP1*, whereas in the case of *SlDEA1*, it was found to be editing at the down-stream of the PAM site (**Figure 4C**). From the plants under bacterial wilt stress, *SlHyPRP1* showed prominent editing at the sgRNA as well as the near upstream of the PAM site (**Figure 5C**).

### 3.3 Off-target analysis

**Figure 6:**
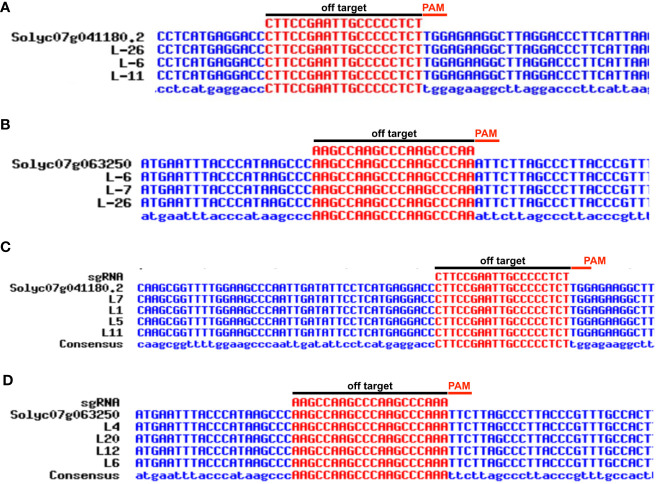
Potential off-target sites generated in transient and stable (GEd0) CRELs by Sanger sequencing. Sequences of CRISPR-edited plant lines (L-6, L-7, L-11, and L-26) were taken for potential off-target analysis and aligned with reference WT by Multalin online alignment tool for **(A)** *SlHyPRP1* and **(B)** *SlDEA1* in transiently transformed lines. Sequences of CRISPR plant lines (L-1, L-4, L-5, L-6, L-7, L-11, L-12, and L-20) were taken for potential off-target analysis and aligned with reference WT by Multalin online alignment tool for **(C)** *SlHyPRP1* and **(D)** *SlDEA1* in stably transformed lines.

To identify the potential off-targets, the sgRNAs of each gene were subjected to CRISPR-P 2.0 (http://crispr.hzau.edu.cn/CRISPR2/). The off-target genes displayed in *Solanum lycopersicum* (SL 2.50) were selected. For experiments and validation primers of the off-target genes were designed in Primer3 and Benchling online tools (**Table 1**; **Supplementary Table S3**). The PCR products were used for Sanger sequencing. The data for Sanger sequencing alignment is presented in **Figure 6**.

**Table 1:** An update on the CRISPR/Cas9 editing events of *SlHyPRP1* and *SlDEA1* and multi-stress tolerance responses in transient *S. lycopersicum* L. system.

CRISPR editing pattern	Multiple stress imposed (abiotic and biotic)	Multiple stress tolerance response of CRISPR/Cas9 editing of *SlHyPRP1* and *SlDEA1*		
		Phenotypic analysis	Physiological/histochemical analysis	Biochemical analysis
Deletion (*SlDEA1*)	Drought	Tolerant	Tolerant	Tolerant
Insertion (*SlHyPRP1*)	Salt	Tolerant	Tolerant	Tolerant
InDel (*SlHyPRP1* and *SlDEA1*)	Bacterial leaf spot (*X. campestris*)	Tolerant	Tolerant	Tolerant
InDel (*SlHyPRP1*)	Bacterial wilt (*R. solanacearum*)	No visible phenotype	Tolerant	Tolerant

### 3.4 Drought stress tolerance response of CRISPR-edited *SlDEA1* in *S. lycopersicum* L.

In disease-susceptible *S. lycopersicum* L. cv. Arka Vikas, CRISPR-positive transformants of *SlHyPRP1* and *SlDEA1* were pre-confirmed by histochemical GUS staining (**Figure 2A**). The putative CRISPR-positive transformants were molecularly confirmed by PCR with Cas9-specific primers (**Figure 2B**).

With morphological and phenotypic observations, the CRISPR-edited leaves (CRELs) were shown to be better responsive to drought stress than WT (empty vector-treated) plants (**Figure 2D**). A physiological and biochemical analysis of CRELs was performed to evaluate the drought stress responses in comparison to WT plants imposed with drought stress. The chlorophyll a and chlorophyll b levels of CRELs were found to be significantly higher than the WT plants (**Figure 2E**i-iii). CRISPR-edited (treated) showed a Chl. a level of 10-13 mg/g fresh wt. compared to WT (treated) which showed 3–5 mg/g fresh wt. The mock plants showed a Chl. a level of 15 to 16 mg/g fresh wt. Similarly, CRELs showed a Chl. b level of 11–13 mg/g fresh wt. compared to WT (treated) which showed 3 to 4 mg/g fresh wt. The mock plants showed a Chl. b level of 15 to 16 mg/g fresh wt. Similarly, the total Chl. content in mock plants used showed 15 to 16 mg/g fresh weight, whereas the CRISPR-edited (treated) was found to be 11 to 12 mg/g fresh weight as compared to WT (treated) which was 4 to 5 mg/g fresh wt. The untreated mock plants showed total a Ch. content of 11 to 12 mg/g fresh wt.

The biochemical assay was performed by measuring the proline content of CRELs in comparison to WT (empty vector-treated) induced by drought stress. A highly significant elevation of proline content was observed in CRELs than in WT (empty vector-treated) plants (**Figure 2E**iv). The CRISPR-edited (treated) plants showed a proline content of approximately 20 µM/g fresh weight compared to WT (empty vector-treated) plants which showed 9 to 10 µM/g fresh weight. The mock plants showed a proline content of 5 to 6 µM/g fresh weight.

### 3.5 Salt stress tolerance response of CRISPR-edited *SlHyPRP1* in *S. lycopersicum* L.

With morphological and phenotypic observations, the CRISPR-edited leaves (CRELs) were shown to be better responsive to salt stress than WT (empty vector-treated) stress-treated plants (**Figure 3D**). The CRISPR-edited leaves (CRELs) were fresher and greener in color as compared to WT plants, and a physiological and biochemical analysis of CRELs was performed to evaluate the salt stress responses in comparison to WT plants imposed with salt stress. During salt stress, the leaves of the plants used to become pale yellow and crunchy in texture, making it difficult for stomatal opening and closing, which was also observed in our study (Gharsallah et al., 2016) (**Figure 3D**). Less leaf damage was observed in CRELs as compared to the WT, whereas the mock plant leaves were growing normally. The CRELs showed a Chl. a level of 10 to 11 mg/g fresh wt. compared to WT which showed 7 to 8 mg/g fresh wt. The mock plants showed a Chl. a level of 13 to 14 mg/g fresh wt. (**Figure 3E**i–iii). The CRELs likewise showed a Chl. b level of 10 to 11 mg/g fresh weight compared to WT which showed 4–6 mg/g fresh weight. The mock plants showed a Chl. b level of 13 to 14 mg/g fresh wt. Similarly, the total Chl. content in CRELs was found to be 13 to 14 mg/g fresh wt. as compared to WT which was 12–14 mg/g fresh wt. The untreated mock plants showed a total chlorophyll content of 13 to 14 mg/g fresh wt. like in drought stress (**Figure 3E**i–iii). The biochemical assay was also performed in salt stress by measuring the proline content, wherein a significantly higher proline content in CRELs was observed in comparison to WT-treated plants induced by salt stress (**Figure 3E**iv). The CRELs showed a proline content of 27 to 28 µM/g fresh wt. compared to WT which showed 18–20 µM/g fresh wt. The mock plants showed a steady level of proline content which ranged at 7 to 8µM/g fresh wt. (**Figure 3E**iv).

### 3.6 Bacterial spot stress (*Xanthomonas campestris* pv. vesicatoria) and bacterial wilt stress (*Ralstonia solanacearum*) tolerance response of CRISPR-edited *SlHyPRP1* and *SlDEA1* in *S. lycopersicum* L. cv. Arka Vikas

In this study, the dual gene CRISPR/Cas9 genome editing of *SlHyPRP*1 and *SlDEA1* showed a significant editing event for both *SlHyPRP1* and *SlDEA1* after performing an alignment of Sanger-sequenced leaf samples in bacterial spot-stressed *S. lycopersicum* L. plants (**Figure 4C**).

With morphological and phenotypic observations, the CRISPR-edited leaves (CRELs) were shown to be better responsive to *X. campestris* stress (**Figure 4D**). The symptoms started developing from 4 to 5 dpi when light brown lesions started to appear on the older leaves, which eventually turned into dark brown spots (**Figure 4D**). The leaves were collected between 15 and 20 dpi and examined for disease progression study by doing ROS assays and cell death count through DAB and trypan blue (TB) staining, respectively (**Figure 4E**i–v). It is evident from the ROS assays that the WT-X.cv-treated leaves showed a higher accumulation of ROS represented by dark brown DAB-stained areas as compared to the CREL leaves (**Figure 4E**ii, iii). Similarly, the occurrence of cell death was also observed to be higher in WT-*X. campestris*-treated leaves as compared to the CREL leaves (**Figure 4E**iv, v). The level of ROS accumulation and the cell death occurrence per area of infected leaves were significantly higher in WT-treated leaves than the CRELs (**Figure 4**ii, iii). Similarly, in the case of bacterial wilt stress assay, the CRISPR-edited lines infected with *R. solanacearum* showed editing in the *SlHyPRP1* gene within the sgRNA target region as well as upstream of the PAM region (**Figure 5C**). No visible phenotypic changes were observed while comparing the wild-type-treated and CRISPR-edited lines (**Figure 5D**). However, the DAB staining of the roots revealed that it still had an impact on the colonization of *R. solanacearum*, and a significantly higher accumulation of ROS was observed in WT-treated roots than the CRELs (**Figure 5E**i, ii).

### 3.7 Replication of CRISPR/Cas9 genome editing through stable plant transformation and confirmation of editing events in CRISPR-edited lines (GEd0 and GEd1) in *S. lycopersicum* L.

After observing the potential and effective role of *SlHyPRP1* and *SlDEA1* as negative multiple stress regulatory genes in the transient *S. lycopersicum* L. system, it was essential to establish and replicate the CRISPR/Cas9 genome editing through stable plant transformation. The CRISPR/Cas9 constructs of *SlHyPRP1 and SlDEA1* were co-transformed into *Agrobacterium tumefaciens* and generated stable lines in cultivar Arka Vikas.

**Figure 7:**
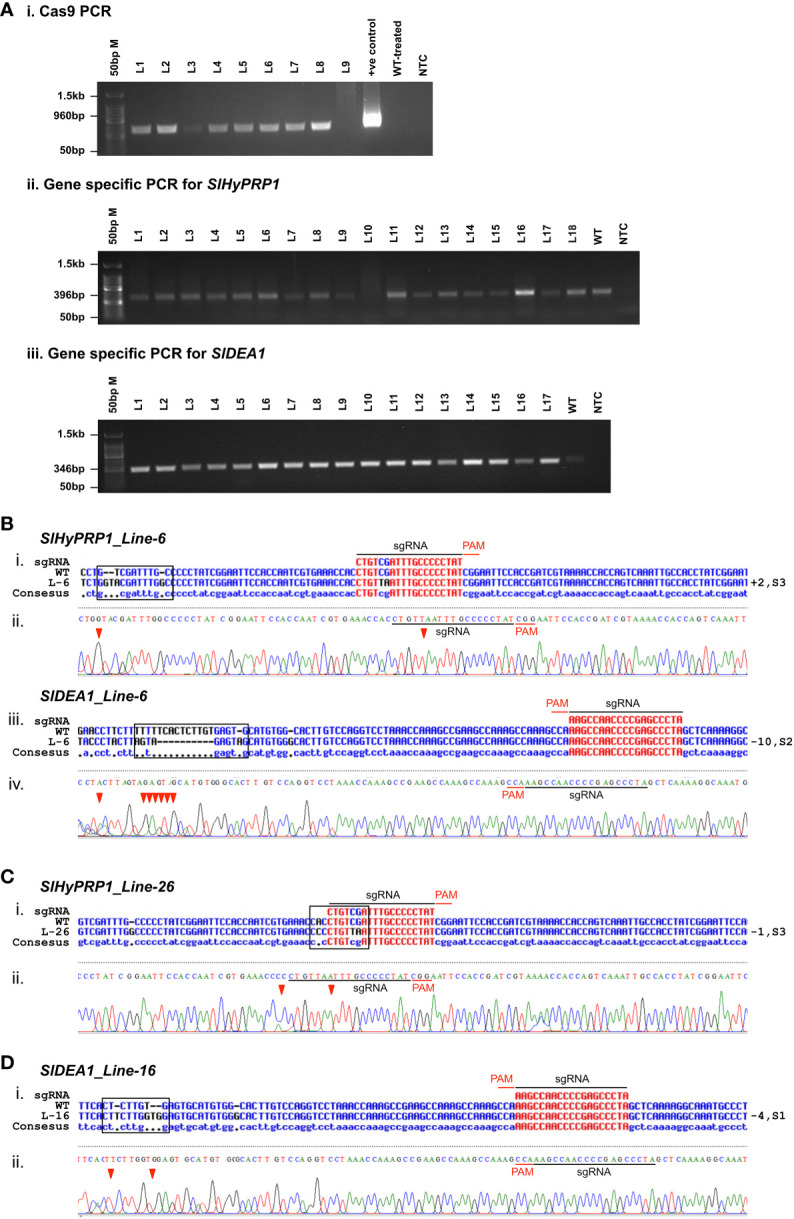
Confirmation of *Agrobacterium*-mediated transformation of CRISPR/Cas9 constructs in *S. lycopersicum* L. cv. Arka Vikas. **(A)** Confirmation of transformation with Cas9 primers (i). PCR with gene-specific primers of *SlHyPRP1* and *SlDEA1* (ii, iii). **(B)** Sanger sequencing confirmation of dual gene *SlHyPRP1* and *SlDEA1* Line-6 (i-iv). **(C)** Single gene editing of *SlHyPRP1*-Line-26 (i, ii). **(D)** Single gene editing of *SlDEA1*-Line-16 (i, ii).

To determine the CRISPR/Cas9-induced mutagenesis in *S. lycopersicum* L. (GEd0) lines, genomic DNA was isolated, and positive transformants were confirmed by PCR using Cas9 primers (**Figure 7A**; **Table 2**). Direct-sequenced PCR products were evaluated for editing events using Multalin, a freely available online sequence alignment tool (Corpet, 1988). This is one of the most reliable online alignment tools which is based on the conventional dynamic programming method of pairwise alignment (Corpet, 1988). The functioning is quite simple and straightforward and shows raw alignments with no errors. For error-free data analysis, the sequencing files were manually checked for each line. The tissue culture regeneration and genetic transformation of *S. lycopersicum* L. cultivars have been improvised in the model and cultivated *S. lycopersicum* L. cultivars of late (Abu-El-Heba et al., 2008; Cruz-Mendívil et al., 2011). However, in multiple-stress-susceptible Arka Vikas cultivars, the tissue culture and genetic transformation have shown 53.2% efficiency (Manamohan et al., 2011). Furthermore, with little modifications, in this study it was found that out of 120 explants that were regenerated, 32 plants were hardened under greenhouse conditions. Among them, 28 plants were found to be positive transformants with the presence of Cas9 (**Supplementary Figure S9**). The efficiency of transformation and editing of GEd0 plants of *S. lycopersicum* L. cv. Arka Vikas is given in **Table 2**. Importantly, both single- and dual-gene editing were observed in stably transformed lines similar to the observation of the transient study. Out of 28 Cas9-positive transformants, two lines (line no. 2 and line no. 6) showed dual-gene editing, whereas single-gene editing was obtained in three lines for *SlHyPRP1* and in five lines for *SlDEA1.* The rest of the 18 lines did not show any editing. A part of the editing pattern was shown, where in line no. 6 both *SlHyPRP1* and *SlDEA1* showed deletion and substitution mutations within and upstream and downstream of the sgRNA target region (**Figure 7B**i–iv). A part of single-gene editing pattern is also shown in **Figures 6C**i-iii, Di-ii and **Supplementary Figure S10**.

**Table 2:** The efficiency of transformation and frequency of editing events in *SlHyPRP1* and *SlDEA1*.

No. of ex-plants	No. of plants hardened	Cas9-positive plants (efficiency)	Editing efficiency (%)			
120	32	28 (87.5%)	Single gene (*SlHyPRP1*)	Single gene (*DEA1)*	Dual gene (*SlHyPRP1+DEA1)*	No editing
			3 (10.71%)	5 (17.85%)	2 (7.14%)	18 (64.28%)
			Pattern of editing			
			Large deletion, insertion and substitution	Deletion and substitution	Deletion and substitution	
			Single gene (*SlHyPRP1*)	Single gene (*DEA1)*	Dual gene (*SlHyPRP1 + DEA1)*	

### 3.8 Potential off-target analysis for *SlHyPRP1* and *SlDEA1 S. lycopersicum* L. cv. Arka Vikas

To evaluate whether the potential off-target effects are caused by CRISPR/Cas9 other than the targeted sgRNA of *SlHyPRP1* and *SlDEA1* of the entire *S. lycopersicum* L. genome, the potential off-target sites were identified using CRISPR P software tool (Liu et al., 2017). In the present study, off-target sites have been selected with less than 3- to 4-bp mismatches, and specific primers were designed using primer3 and Benchling cloud-based platform. CRISPR lines (L-6, L-11, L-26, L-7) were randomly chosen for the off-target analysis in GEd0. The amplified PCR product of the CREL plants covering the off-target sites with corresponding primer pairs was analyzed by Sanger sequencing (**Figures 6A**–**D**; **Supplementary Table S3**). The sequencing data showed no mutations at the sites of off-target, which suggests that the CRISPR/Cas9 editing of *SlHyPRP1* and *SlDEA1* was target-specific and the multiple-stress tolerance response is because of the target-specific editing of these two genes.

## 4 Discussion

### 4.1 CRISPR/Cas9 editing events of *SlHyPRP1* and *SlDEA1* genes in transient and stable genetic transformation are replicable

From the current study, one major upshot was the CRISPR/Cas9 editing efficiency of the sgRNA designed for *SlHyPRP1* and *SlDEA1* in transient as well as in stable *S. lycopersicum* L. lines. The single-guide RNAs for both genes have been designed using the popular and efficient guide RNA designing tools CC-Top, CRISPR-P, and Chop-Chop (Lei et al., 2014; Stemmer et al., 2015; Labun et al., 2019). The sgRNAs were designed at the functional domain, they were successfully able to guide the Cas9 effectively to make precise double-stranded breaks on the target genes, and there was no off-target activity, which is one of the crucial components of the genome editing approach.

Overall, the CRISPR/Cas9-based transient *S. lycopersicum* L. system has shown insertion, deletion, and substitution mutations upstream and downstream of the PAM site of both *SlHyPRP1* and *SlDEA1* in the plants taken for the given four stresses. In case of drought stress, the leaves of the edited lines showed insertion mutation for *SlDEA1* within the sgRNA target region that caused the frameshift mutation (**Figure 2C**). Similarly, the leaves of the edited lines exposed to salt stress showed a deletion mutation upstream of the PAM sequence for *SlHyPRP1* (**Figure 3C**). This pattern of editing is quite common in Cas9-mediated double-stranded DNA breaks. In a targeted mutagenesis in soybean protoplast, Cas9-mediated editing was achieved successfully on the three target genes, namely, *Glyma12g37057*, *Glyma08g02290*, and *Glyma0614180*, with no off-target activity and with a mutation efficiency of 3.2%–9.7% (Sun et al., 2015). The editing pattern in both *Glyma12g37057* and *Glyma0614180* was achieved upstream of PAM, but, interestingly, in the case of *Glyma08g02290*, editing was observed both upstream and downstream of the target site in the form of small (single base pairs) to large deletion and substitutions (single to multiple base pairs). Recently, in another study, a precise CRISPR/Cas9-mediated multiplex editing in the *SlHyPRP1* gene has been achieved at the functional domains upstream of the PAM site (Tran et al., 2021). Similarly, in the case of biotic stresses (bacterial leaf spot and bacterial wilt disease), the transient edited lines showed INDEL as well as substitution mutations both upstream and downstream of the seed sequence of *SlHyPRP1* and *SlDEA1*. However, in the case of bacterial wilt stress, no editing could be achieved in *SlDEA1*, but *SlHyPRP1* showed INDEL mutations with the sgRNA target site as well as in the deletion upstream of the sgRNA target site. When tomato *eIF4E1* was targeted by Cas9 to enhance resistance against potyvirus, it produced homozygous mutations in transgene-free GEd1 lines, whereas GEd0 lines produced deletion mutations ranging from 11 to 43 base pairs both upstream and downstream of the PAM sites (Yoon et al., 2020). Interestingly, the same pattern of editing events was achieved in the current study as well. A transformation efficiency of 87.5% was obtained in stable *S. lycopersicum* L. Among the Cas-positive transformants, 10.7% showed *SlHyPRP1* editing, 17.8% showed *SlDEA1* editing, and 7.1% showed dual-gene editing (**Table 2**; **Supplementary Figure S10**). This makes the evidence stronger in such a way that the selected guide sequences are efficient enough to produce effective editing on the target genes. In the cv. Micro-Tom, an *Agrobacterium*-mediated transformation has shown 72% of transformation efficiency for *eIF4E1*, with 3.5% of editing efficiency in the PDS gene and with both copies edited (Yoon et al., 2020).

Significantly, in the stable transformation of the present study, both single-gene and dual-gene editing were achieved. GEd0 line 26 showed a deletion of one base pair along with substitution at three locations upstream of the sgRNA target site of *SlHyPRP1*. For *SlDEA1*, GEd0 line 16 showed deletion and substitution mutation at four sites downstream of the PAM, whereas GEd0 line6 has shown dual-gene editing of both *SlHyPRP1* and *SlDEA1* upstream and downstream of the sgRNA target site. The editing was achieved in the form of small to large insertions (+2 bp)/deletions (-10 bp) and substitutions (**Figure 7**; **Supplementary Figure S9**). These mutations resulted in frameshift mutations which caused the gene to be non-functional. In a previous study in Micro-Tom, the Cas9-mediated editing observed in the PDS gene was INDEL mutation of three base pairs upstream of PAM, which led to an early stop codon causing the loss of PDS gene function (Yoon et al., 2020), whereas in the case of *eIF4E1*, INDEL mutation was observed at different locations and sometimes beyond the PAM site, creating a premature stop codon and truncated *eIF4E1* protein. A 29-bp deletion has also been achieved (Yoon et al., 2020). In the targeted mutation in three genes of soybeans through CRISPR/Cas9, a range of 14.7%–20.2% mutation efficiency was achieved, along with a biallelic mutation in a T0 line which led to the desired phenotype (Sun et al., 2015). Very recently, an attempt has been made to produce DNA-free CRISPR/Cas9 mutation in wild tomato targeting several important genes, including genes involved in defense against diseases like yellow leaf curl virus and other pathogenesis-related genes (Lin et al., 2022). Using vectors carrying the Cas9 and sgRNA via *Agrobacterium*-mediated transformation, they targeted three important genes: *SpSGS3*, *SpRDR6*, and *SpPR1*. They got 8.3%, 13.2%, and 13.9% mutation efficiency in the three genes, respectively. In our study, a similar pattern of gene editing events for *SlHyPRP1* and *SlDEA1* was observed in GEd1 lines of *S. lycopersicum* L. (**Supplementary Figure S9**).

### 4.2 CRISPR/Cas9 editing of *SlHyPRP1* and *SlDEA1* leads to multi-stress tolerance in *S. lycopersicum* L. cv Arka Vikas

Even though in the stable lines the editing events were successfully achieved, a systematic stress analysis in the transient lines showed prominent/sufficient data to prove the hypothesis.

#### 4.2.1 A potential role of *SlDEA1* in the negative regulation of drought stress tolerance

**Figure 8:**
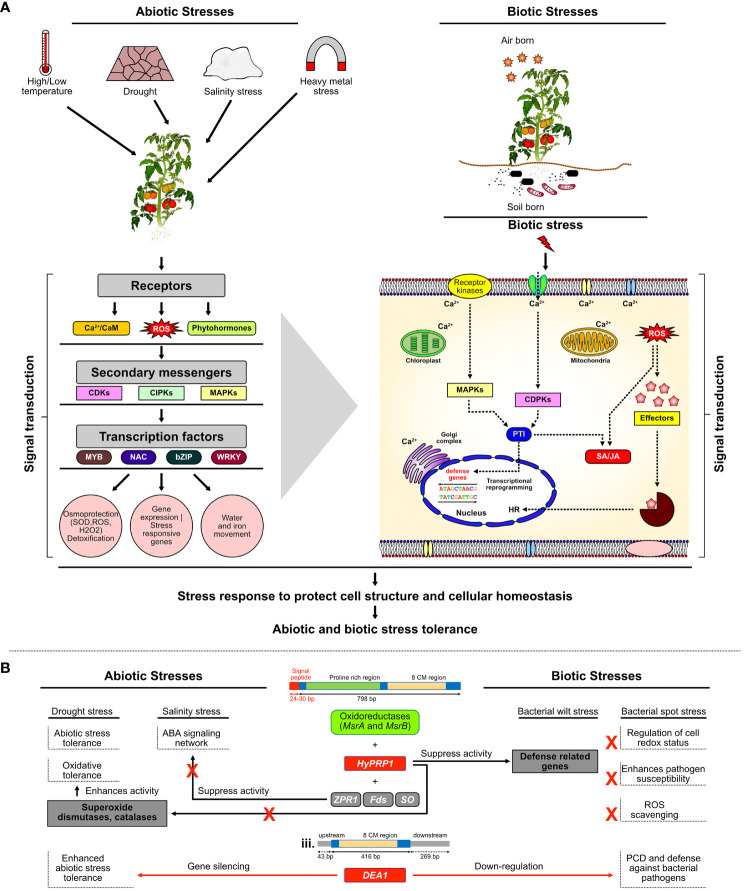
Mechanism and genetic regulation of multi-stress responses in plants. **(A)** Under MAPKs module, when plants experience stresses such as salt, drought, cold, or pathogen attack, the production of ROS scavengers functions as the signal to the first line of defense. Osmotic or oxidative stress occurs due to salt, drought, cold, or pathogen which triggers sensors or receptor proteins to activate various protein kinases such as MAPKs and signaling cascade to restore cellular osmotic homeostasis. **(B)** Proposed model of SlHyPRP1- and SlDEA1-mediated negative regulatory mechanism of multi-stress tolerance in *S. lycopersicum* L.

During drought stress, plants try to cope with water scarcity by halting growth and reducing photosynthesis and other plant processes to reduce water use, resulting in the discoloration of leaves and foliage wilting, eventually leading to plant death. The basic research has produced notable findings in the understanding of the plants’ complex physiological and molecular responses to drought, but a large research gap between yield-favorable and stress conditions is still not explored (Khan, 2015) (**Figure 8A**). That gap can be filled by molecular biology approaches by targeting genes best known to be positive or probable negative regulators of stress. A notable finding in the present study is that the CRISPR/Cas9 editing of negative stress regulatory gene *SlDEA1* has caused the frameshift mutation distorting the function of the gene. This probably has contributed to protecting against damage to the chloroplast by ROS and thus maintaining the chlorophyll content during drought stress (Mafakheri et al., 2010) (**Figure 8B**).

In general, drought stress inhibits the regular photosynthesis process in plants by affecting the level of chlorophyll contents and chlorophyll components and by damaging the photosynthetic apparatus (Iturbe-Ormaetxe et al., 1998; Mafakheri et al., 2010). However, the principal factor for limiting photosynthesis is lower stomatal conductance to conserve water and stomatal diffusive resistance to CO_2_ entry (Mafakheri et al., 2010). In crops like sunflower under drought stress, there was a large decline in chlorophyll a, chlorophyll b, and total chlorophyll contents (Manivannan et al., 2007). Various studies have shown that water stress has led to a decrease in chlorophyll pigments in tomato, followed by changes in leaf water potential, stomatal resistance, and protein content in leaves (Nayyar et al., 2005; Zgallaï et al., 2006). Changes in the level of osmoprotectants like proline has also enlightened the potential negative regulatory role of *SlDEA1* during drought stress (**Figure 8B**). The loss-of-function of *SlDEA1* might have contributed to maintaining the levels of osmoprotectants, mainly proline, to restore or balance its level in *S. lycopersicum* L. Proline is known to protect structures of folded protein from denaturation, to stabilize cell membrane by interacting with phospholipids, and also to function as a hydroxyl radical scavenger or to serve as an energy or nitrogen source (Claussen, 2005). Proline is one of the most common compatible osmolytes for a wide range of stresses such as drought, salinity, extreme temperatures, and high light intensity. It has been observed that an increased level of proline under mild drought can allow plants to survive under stress (Claussen, 2005; Mafakheri et al., 2010). Proline does not also interfere with normal biochemical reactions; its accumulation can influence adaptive responses being a part of the stress signal (Maggio et al., 2002; Mafakheri et al., 2010). When chickpea varieties were subjected to drought stress, it was observed that the proline content increased in all growth stages, but especially in the vegetative stage, it was about 10-fold higher, which led to osmotic compatibility that resulted in the avoidance of drought stress in chickpea (Mafakheri et al., 2010). When the effects of drought stress were also studied in a tomato cv. Bombino, due to a decrease in drought stress conditions, the proline content in cell sap increased (Khan, 2015). In recent studies, *SlHyPRP1* has been shown to function as a negative regulator of salt and drought stress—for instance, multiplexed CRISPR/Cas9 genome editing of negative multi-stress regulatory gene *SlHyPRP1* in *S. lycopersicum* L. showed a higher survival rate than WT-treated plants under salt stress (Tran et al., 2021; Tran et al., 2023). However, in our current study, the significantly elevated levels of chlorophylls and proline content in the CRELs of *SlDEA1* than WT plants revealed the role of *SlDEA1* as a negative regulator along with *SlHyPRP1*, a known negative regulator in imparting genetic tolerance to drought stress in *S. lycopersicum* L. (**Figure 8B**; **Table 1**).

#### 4.2.2 *SlHyPRP1* in the negative regulation of salt stress tolerance

In our recent study, we reported that SlHyPRP1 and SlDEA1 strongly interact with each other at the plasma membrane and cytoplasm, revealing their site of function for stress tolerance (Saikia et al., 2020a; Saikia et al., 2020b). As a follow-up, our current study also showed significant results, wherein both chlorophyll and proline content were found to be higher in *SlHyPRP1*-edited lines than WT under salt stress. In general, the presence of excessive salt in soil inhibits water uptake by plants, which causes ionic imbalance leading to ionic toxicity and osmotic stress. Hence, to cope with this, plants accumulate similar solutes like proline, which is known to decrease osmotic potential, promote water absorption, and help in ROS scavenging (**Figure 8A**), though different signaling pathways lead to the expression of genes that, in turn, allow the activation of the proteins that determine plant phenotype under salt stress (Gharsallah et al., 2016). However, proline accumulation is one of the many plant adaptations to salinity and water deficit (Kahlaoui et al., 2018). Similarly, the chlorophyll contents in plants are also affected by salt stress. *HyPRP1* from wild tomato, when subjected to knockdown by RNAi approach, also causes the tomato plants to show enhanced tolerance to various abiotic stresses, including salinity stress (Li et al., 2016). In several plants including tomato, salt stress severely affects the chloroplast structures and decreases the chlorophyll content, resulting in a reduced photosynthetic rate (Yang et al., 2020). The direct effect of salt stress in plants is achieved by regulating the activity and expression levels of enzymes involved in chlorophyll biosynthesis and photosynthesis (Yang et al., 2020). Previous studies claim that the decrease in Chl. a and Chl. b content during salt stress in *Phaseolus vulgaris* L. and *Vigna subterranean* L. is considered a typical symptom of oxidative stress and causes the inhibition of chlorophyll synthesis as well as activates its degradation by the enzyme chlorophyllase (Taïbi et al., 2016). Proline was reported to be a reliable indicator of environmental stress imposed on hydroponically grown tomato plants (Claussen, 2002). When 20 different tomato cultivars, which were clustered depending on scale classes according to their response to salt, were studied to measure their ion concentration, proline content, antioxidant enzyme activities, and gene expression, it was found that the moderately salt-tolerant cultivars showed higher levels of ion and proline concentration (Gharsallah et al., 2016). In a previous study, two tomato cultivars grown in saline soil with a foliar spray of a low concentration of proline also showed increased tolerance to salt with an elevated level of proline contents (Kahlaoui et al., 2018).

#### 4.2.3 *SlHyPRP1* and *SlDEA1* dual-gene editing imparts bacterial leaf spot and wilt tolerance

Bacterial leaf spot caused by *Xanthomonas campestris* pv. vesicatoria (X.cv) and bacterial wilt caused by *Ralstonia solanacearum* are two devastating diseases of tomato (Maneva et al., 2009; Park & Han, 2017). X.cv can penetrate their host through the leaf stomata, sometimes hydathodes, and through a wounded site in humid conditions, causing small lesions that subsequently turn into small brown spots to appear on leaves, stems, and fruits (Park & Han, 2017). The molecular mechanism underlying the interactions between this pathogen and its hosts reveals that X.cv uses a type III secretion system to secrete effectors into the host cell and where they interact with the cellular processes of the host to promote disease or to elicit a defense response (Tamir-Ariel et al., 2007), whereas *R. solanacearum* is a soil-born saprophytic bacteria that generally enters into the plant’s roots (Kumar et al., 2018). Its vigorous growth in the roots of *S. lycopersicum* L. leads to wilting and eventually causes the death of the host plant. Even though many research on understanding the inherent mechanism of bacterial wilt disease progression caused by *R. solanacearum* have been performed in model crops, they could only reveal the involvement of a few resistant genes like *RRS1*, *RPS4*, *SGT1*, etc., which may take part in conferring resistance against *R. solanacearum*. However, how *R. solanacearum* infection progresses and distributes in model plants like *Arabidopsis* is still unknown (Xu et al., 2022). Moreover, just like X.cv, *R. solanacearum* can also survive in soil and within the weeds dwelling in the same field where the tomato is grown (Kumar et al., 2018). It makes them difficult to eradicate from the crop field, thus leaving a potential long-term damage. Hence, apart from chemical fertilizer applications, it is necessary to develop *S. lycopersicum* L. varieties that can tolerate or are resistant to bacterial spot disease caused by X.cv and *R. solanacearum.*

Generally, in biotic stress regulation, ROS accumulation is one of the PAMP-triggered immunity (PTI), whereas programmed cell death is an effector-triggered immunity (ETI) (Bach-Pages and Preston, 2018). ROS and cell death per total area of infected leaf are one of the key defense responses in plants That means, in a compatible host–pathogen interaction, the pathogen is more easily able to suppress or evade the plant immunity by invading the tissues and causing disease or stress. The production of ROS and hypersensitive response in terms of the local cell as a defense response will be higher in susceptible plants (Bach-Pages and Preston, 2018) (**Figure 8**). This has correlated with the current study as upon *X. campestris* stress, the editing of *SlHyPRP1* and *SlDEA1* and editing of both genes in the *S. lycopersicum* L. leaves might have imparted an enhanced immunity of the plants which, in turn, caused less PTI and ETI production, thereby generating less damage to the CREL leaves as compared to WT leaves. However, when WT-treated plants and the CRELs were exposed to *R. solanacearum* stress, interestingly, there were not many visible symptoms or difference in disease progression observed on the aerial parts of the plants, yet a clear difference in disease progression was observed when the roots of the CRELs and WT-treated plants were examined through DAB staining, which showed that there is a significantly higher accumulation of ROS in WT-treated roots than that of the CREL roots.

The underlying reason could be that *R. solanacearum* is a pathogen that penetrates and colonizes the root cortex of the host plant and multiplies in the xylem tissue to reach the aerial parts and that colonization might have not been enough to cause a subsequent wilting symptom in the whole plant. It was also interesting that, in the case of *R. solanacearum* stress, the editing was obtained in *SlHyPRP1* genes, and it was a single-gene CRISPR editing event. This is possibly the cause of those minimal phenotypic changes on the *S. lycopersicum* L. seedlings and may not have enough functional distortion to produce visible phenotypic changes on the areal part of the seedlings. However, it still had an impact on the colonization of *R. solanacearum* and a significantly higher accumulation of ROS in WT-treated roots than the CRELs. Hence, it can be inferred that CRISPR/Cas9 genome editing of *SlHyPRP1* and *SlDEA1* makes them non-functional or that editing both genes has a significant negative role in the biotic stress regulation of *S. lycopersicum* L. as the host plant. It makes them difficult to eradicate from the crop field, which leaves a potential long-term damage. Hence, apart from chemical fertilizer applications, it is necessary to develop *S. lycopersicum* L. varieties that can tolerate or be resistant to bacterial spot diseases caused by X.cv and *R. solanacearum*.

## 5 Conclusions

The current findings on CRISPR/Cas9-based editing of *SlHyPRP1* and *SlDEA1* genes in a transient *S. lycopersicum* L. system showed a significantly tolerant response to drought, salinity, bacterial leaf spot, and bacterial wilt, indicating their negative regulatory role in imparting multiple stress tolerance. This corroborated with our previous study which showed a strong interaction of *SlHyPRP1* and *SlDEA1* in the cytoplasm and towards the cell periphery, suggesting that they possibly function together in multi-stress regulation. The significance of the current study is that, functional *SlHyPRP1* serves as a major negative regulator of multiple stress response with a strong functional association of *SlDEA1* as an anchor gene in *S. lycopersicum* L. Notably, the single- and dual-gene CRISPR/Cas9 editing of *SlHyPRP1* and *SlDEA1* has been replicated in stable genetically transformed lines of *S. lycopersicum* L. The novel finding of the present study is that *SlDEA1* emerged as a potential negative stress regulatory gene that has been poorly explored so far. Therefore, even though *SlHyPRP1* has been studied in many major crop plants for its negative regulatory role in stress tolerance including *S. lycopersicum* L., a combined loss-of-function effect of both *SlHyPRP1* and *SlDEA1* has been thoroughly characterized for the first time in our study. The authors would like to stress that the functional evaluation of CRISPR/Cas9-edited stable lines (GEd0 and GEd1) generated from our current findings for multiple stress tolerance is beyond the scope of the current study.

The findings of the current study in a transient and stably transformed *S. lycopersicum* L. system would pave the way to evaluate the genetic heritability of CRISPR/Cas9 editing and the development of genetic tolerance to multiple abiotic and biotic stresses in *S. lycopersicum* L. This would have a significant impact to sustain crop productivity to tackle the global climate change scenario.

## Data availability statement

The original contributions presented in the study are included in the article/**Supplementary Material**. Further inquiries can be directed to the corresponding authors.

## Author contributions

BS: Conceptualization, Data curation, Formal analysis, Investigation, Methodology, Project administration, Software, Validation, Visualization, Writing – original draft, Writing – review & editing, Funding acquisition. RS: Data curation, Methodology, Software, Validation, Writing – review & editing. JD: Data curation, Methodology, Software, Validation, Writing – review & editing. JM: Data curation, Formal analysis, Writing – review & editing, Software. GS: Formal analysis, Project administration, Software, Writing – review & editing. CC: Conceptualization, Formal analysis, Funding acquisition, Project administration, Resources, Supervision, Validation, Visualization, Writing – review & editing.
