# Supplementary material for: CRISPR/Cas9-based genome editing and functional analysis of SlHyPRP1 and SlDEA1 genes of Solanum lycopersicum L. in imparting genetic tolerance to multiple stress factors
Source: Front Plant Sci. 2024 Feb 2;15:1304381. doi: 10.3389/fpls.2024.1304381 (PMC10869523; doi:10.3389/fpls.2024.1304381)
Supplement: Supplementary file 1 [file DataSheet_1.pdf]

Supplementary Material

CRISPR/Cas9-based genome editing and functional analysis of *SlHyPRP1* and *SlDEA1* genes of *Solanum lycopersicum* L. in imparting genetic tolerance to multiple stress factors

Banashree Saikia[1,2*], Remya S[1], Johni Debbarma[1,2], Jitendra Maharana[3], G. Narahari Sastry[4] and Channakeshavaiah Chikkaputtaiah[1,2*]

[1]Biological Sciences and Technology Division, CSIR-North East Institute of Science and Technology (CSIR-NEIST), Jorhat-785006, Assam, India

[2]Academy of Scientific and Innovative Research (AcSIR), Ghaziabad, Uttar Pradesh-201 002, India

[3]Institute of Biological Chemistry, Academia Sinica, Taipei 11529, Taiwan

[4]Advanced Computational and Data Science Division, CSIR-NEIST, Jorhat-785006, Assam, India

*** Correspondence:** Corresponding Authors: channakeshav@neist.res.in ; banasreesaikia39@gmail.com

1. Supplementary Figures

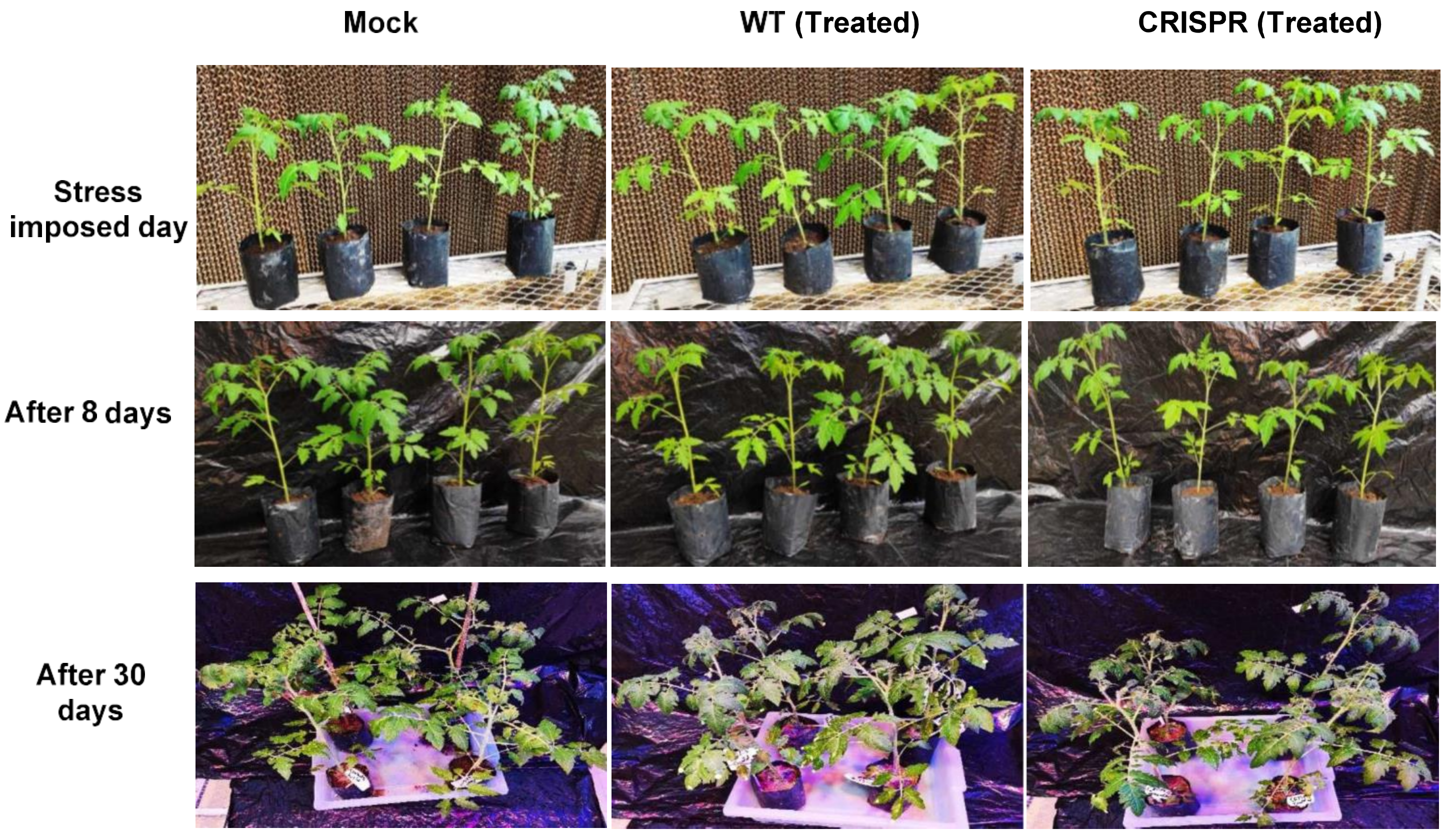

Figure. S1. Analysis of drought stress tolerance response of CRELs in transient *S. lycopersicum* L. system: Agro infiltration was performed and the WT-Treated and CRISPR-edited plants were imposed with drought stress and phenotypic changes were observed (Replicate 1 is given in Fig. 2, Replicate 2 is given here, Replicate 3 not given).

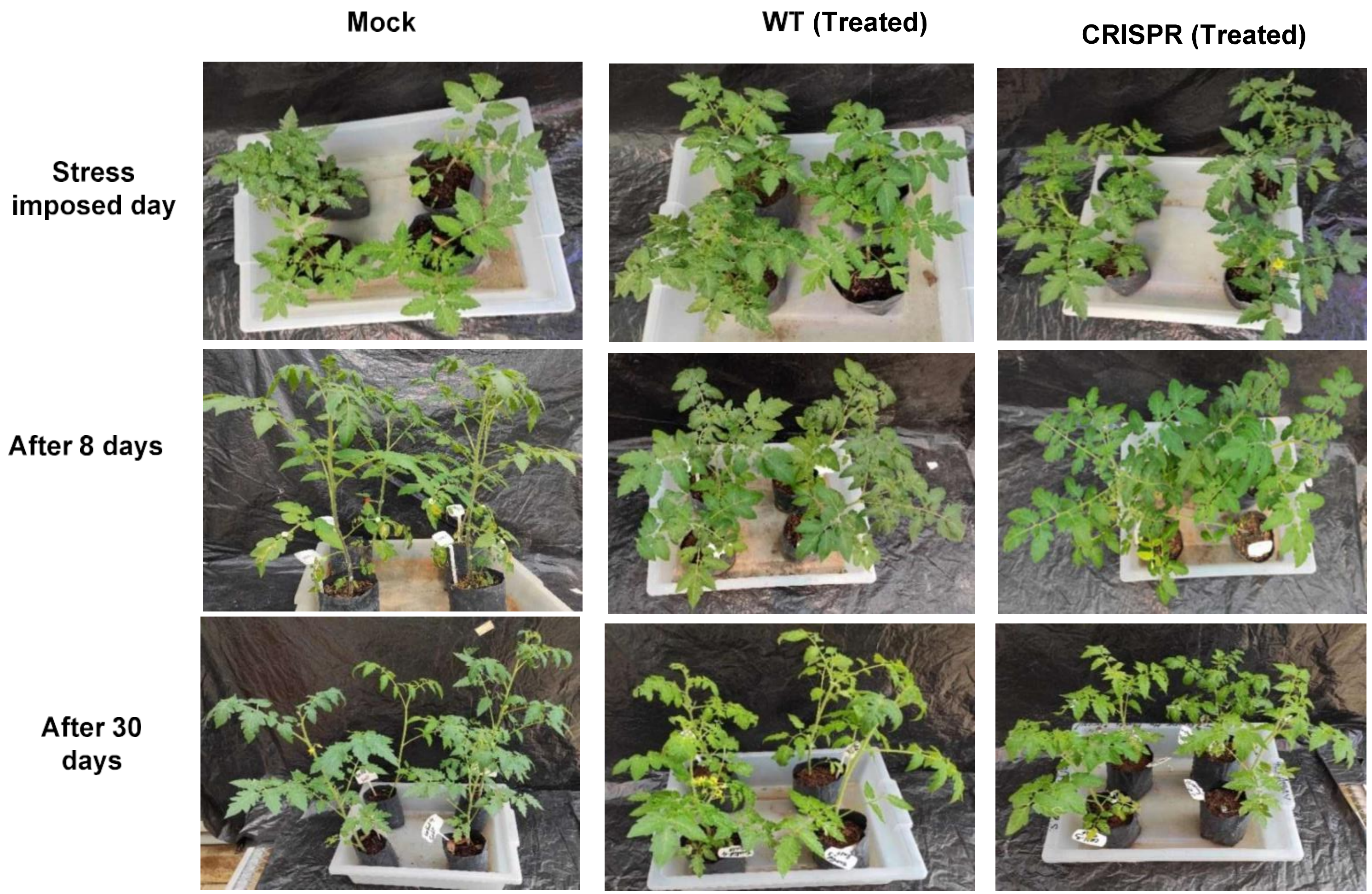

Figure. S2. Analysis of salt stress tolerance response of CRELs in transient *S. lycopersicum* L. system: Agro infiltration was performed and the WT-Treated and CRISPR-edited plants were imposed with salt stress and phenotypic changes were observed (Replicate 1 is given in Fig. 3, Replicate 2 is given here, Replicate 3 not given).

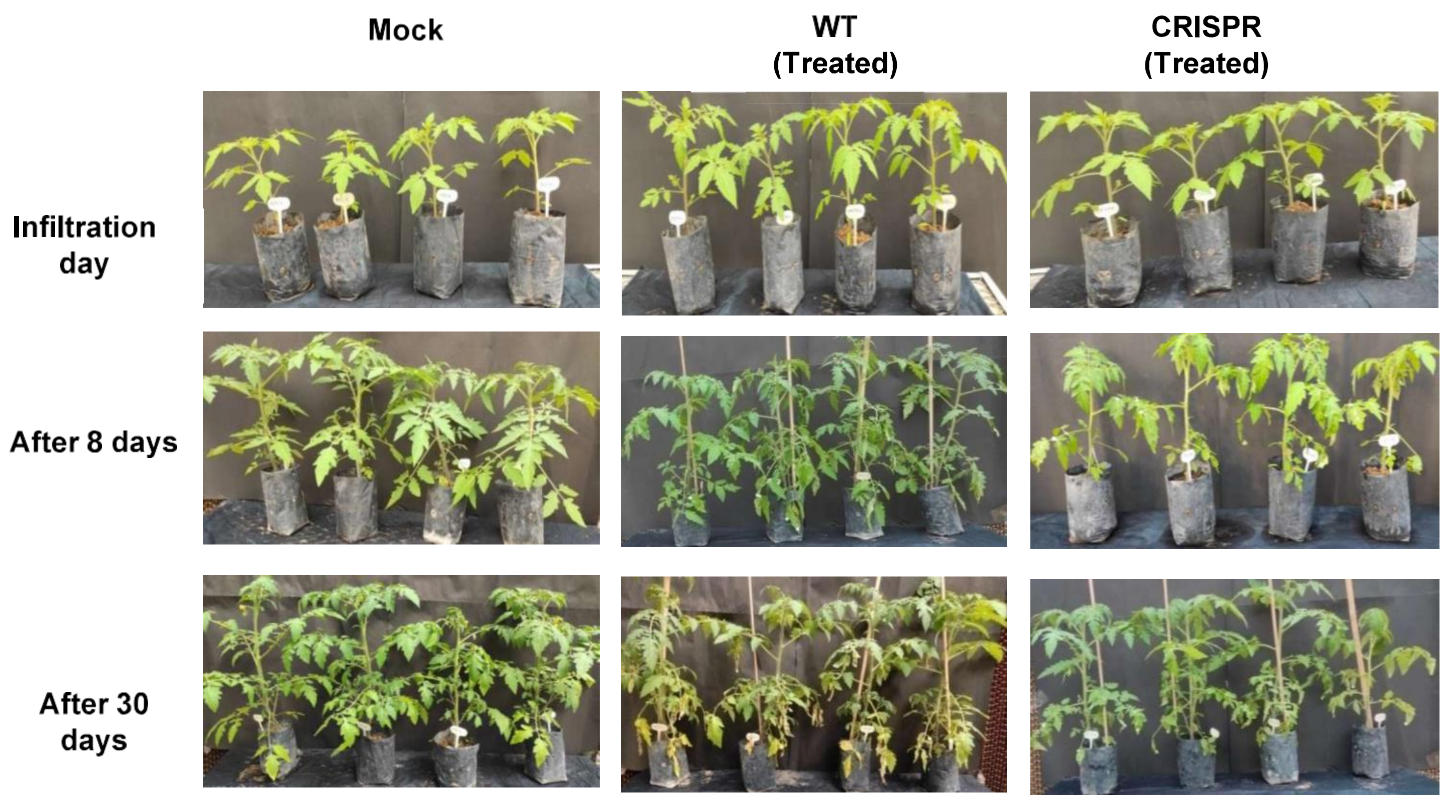

Figure. S3. Analysis of bacterial leaf spot (*X. campestris*) stress tolerance response of CRELs in transient *S. lycopersicum* L. system: Agro infiltration was performed and the WT-Treated and CRISPR-edited plants were imposed with bacterial leaf spot (*X. campestris*) stress and phenotypic changes were observed (Replicate 1 is given in Fig. 4, Replicate 2 is given here, Replicate 3 not given).

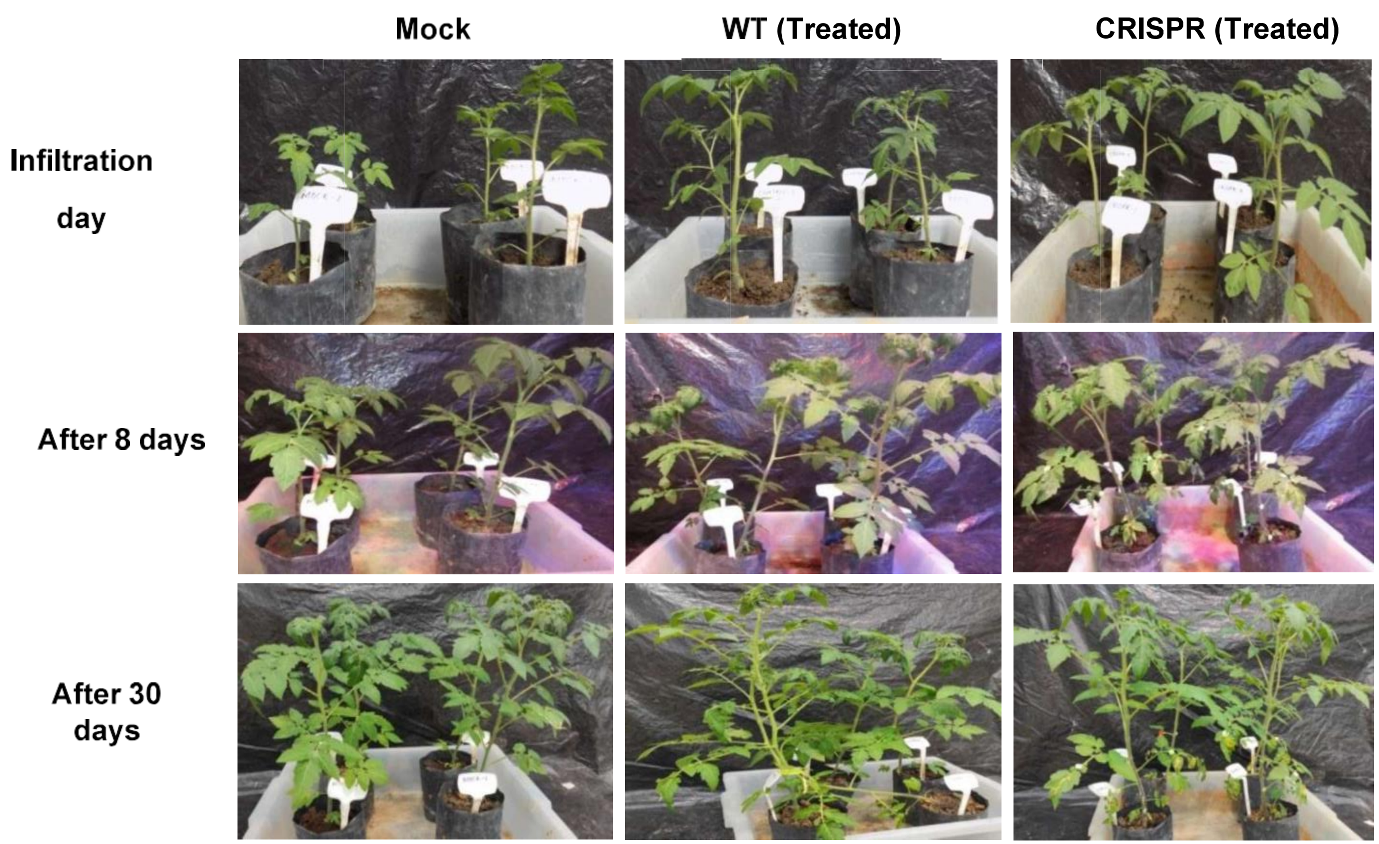

Figure. S4. Analysis of bacteria wilt (*R. solanacearum*) stress tolerance response of CRELs in transient *S. lycopersicum* L. system: Agro infiltration was performed and the WT-Treated and CRISPR-edited plants were imposed with bacterial wilt (*R. solanacearum*) stress and phenotypic changes were observed (Replicate 1 is given in Fig. 5, Replicate 2 is given here, Replicate 3 not given).

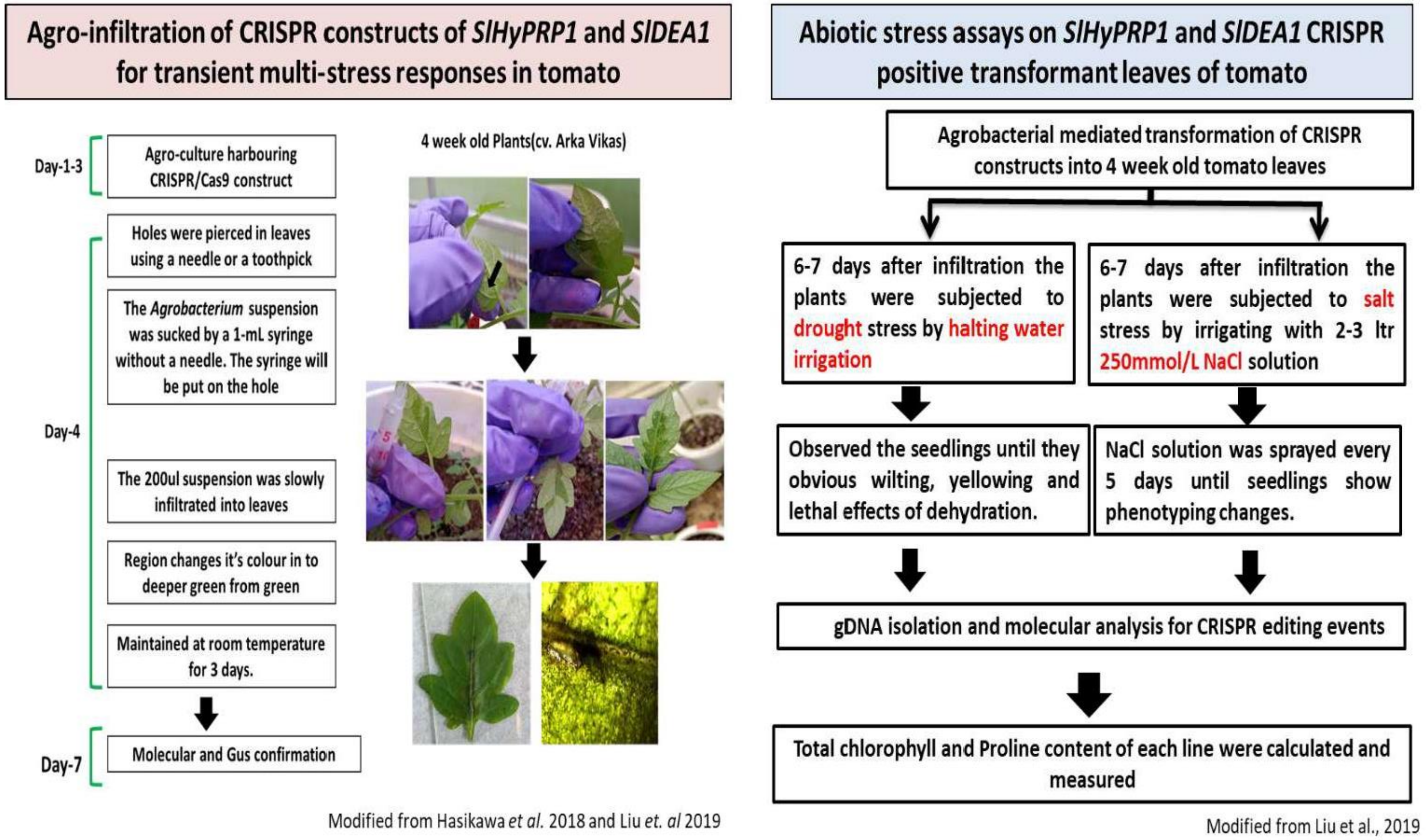

Figure. S5. Methods for transient Agrobacterium-mediated infiltration and imposing abiotic stress: *Solanum lycopersicum* L. (3-4 weeks) leaves were Agro-infiltrated using CRISPR constructs of *SlHyPRP1* and *SlDEA1* for transient analysis and subjected to abiotic stresses such as drought and salinity for their responses. The leaves were pierced and infiltrated with a syringe containing the Agro-suspension and the CRISPR-positive transformants were confirmed through molecular analysis and GUS assays.

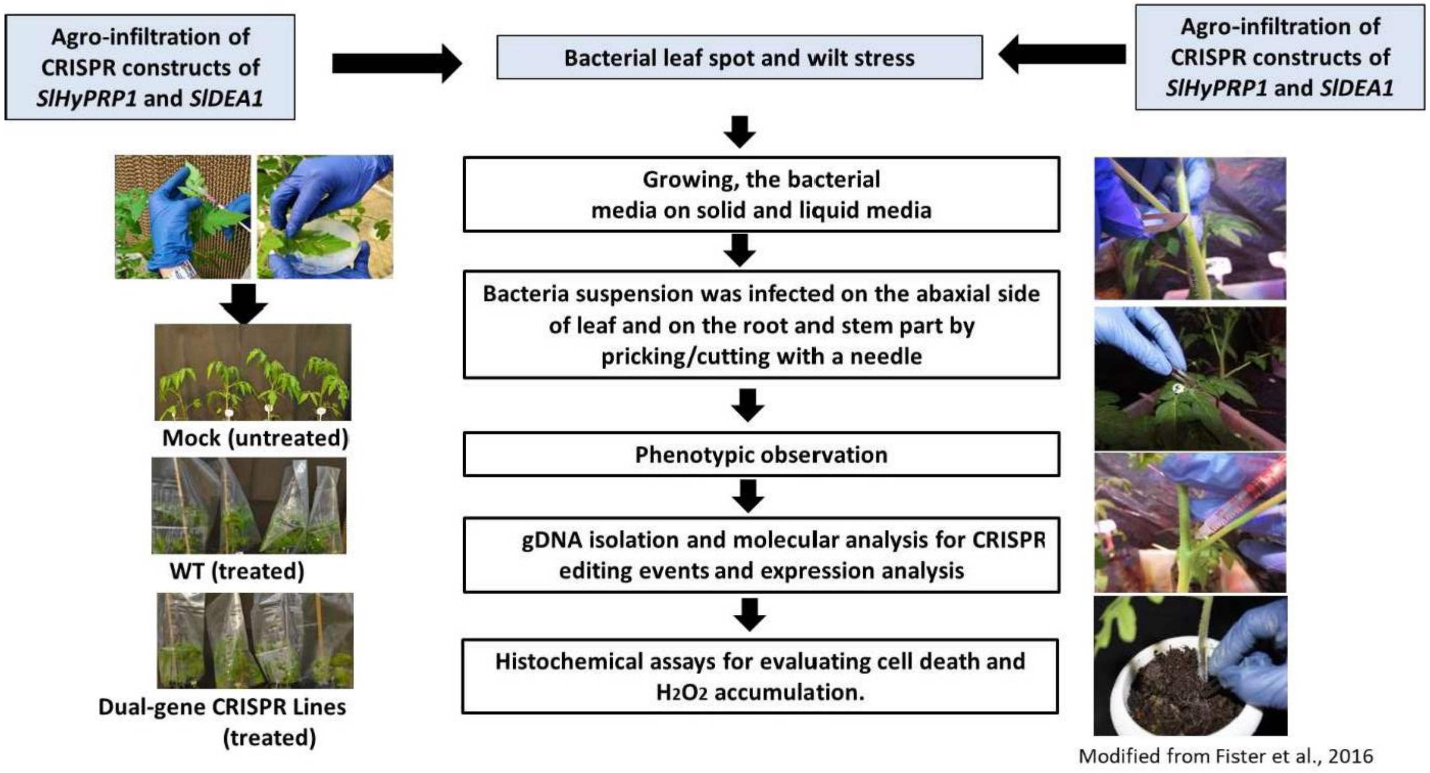

Figure. S6. Methods for imposing biotic stress: Bacterial leaf spot (*X. campestris*) and Bacterial wilt (*R. solanecearum*). The Agro-infiltration was performed as described earlier (Fig. S1), leaves, stem and roots were treated with bacterial suspension of *R. solanacearum* and *X. campestris* and evaluated for their phenotypic responses.

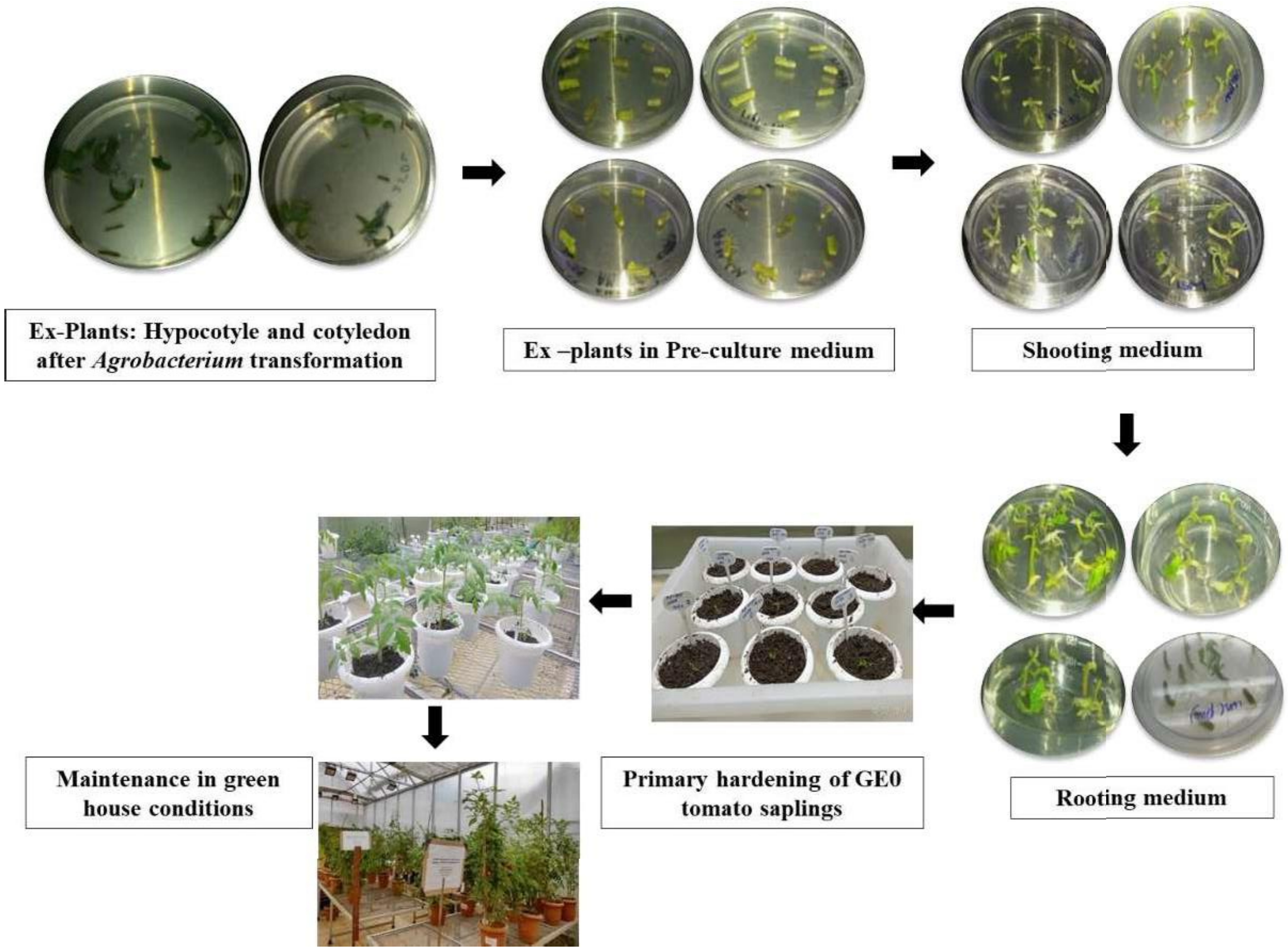

Figure. S7. Standardization of *Agrobacterium*-mediated plant transformation and tissue culture regeneration.

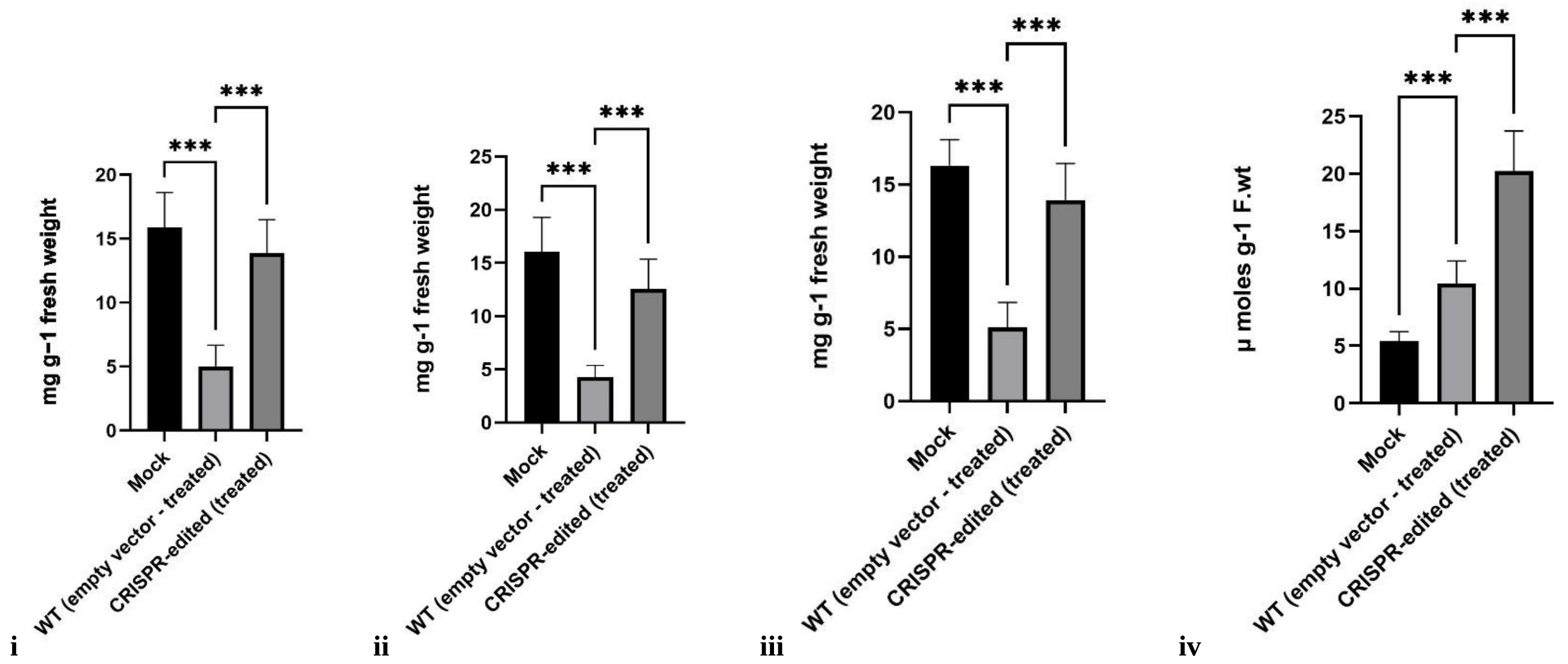

Fig. S8 A. Physiological and biochemical parameters of drought-stressed plants. i. Chl a, ii. Chl b, iii. Ch a&b, iv. Proline content (Replicate 2).

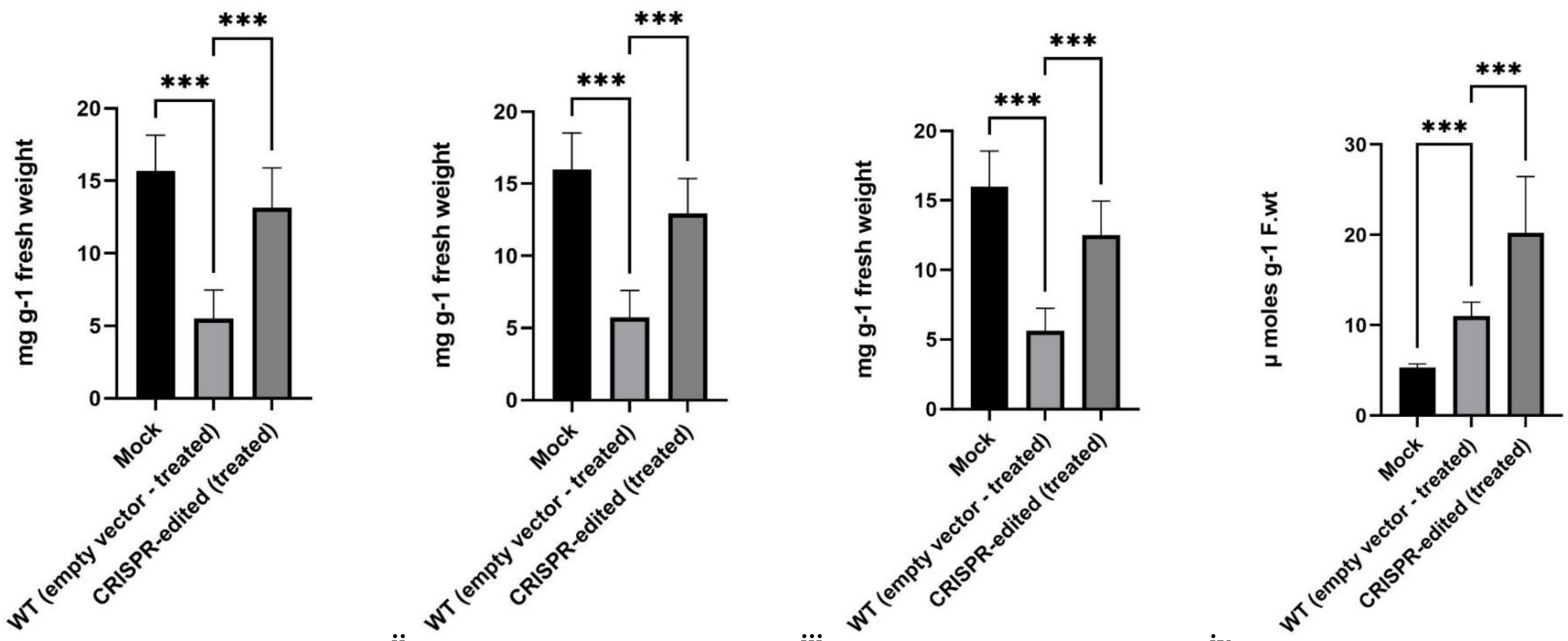

i ii iii iv

Fig. S8 B. Physiological and biochemical parameters of drought-stressed plants. i. Chl a, ii. Chl b, iii. Ch a&b, iv. Proline content (Replicate 3).

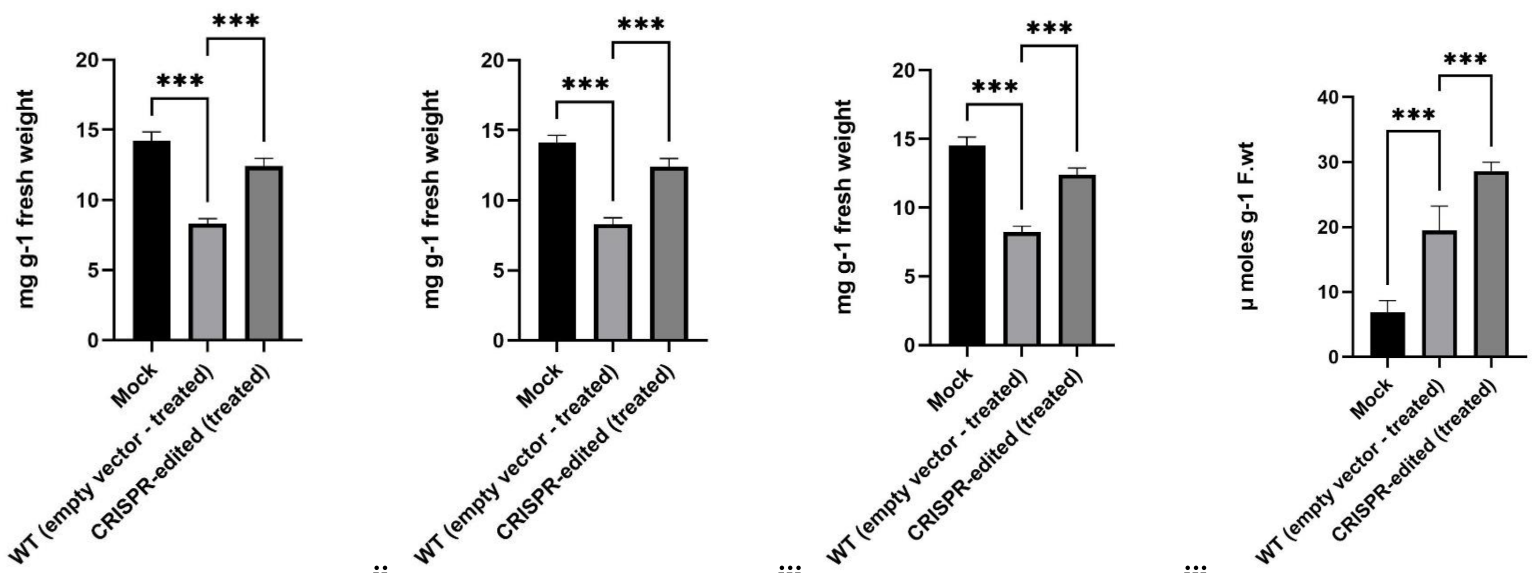

i ii iii iii

Fig S8 C. Physiological and biochemical parameters of salt-stressed plants. i. Chl a, ii. Chl b, iii. Ch a&b, iv. Proline content (Replicate 2).

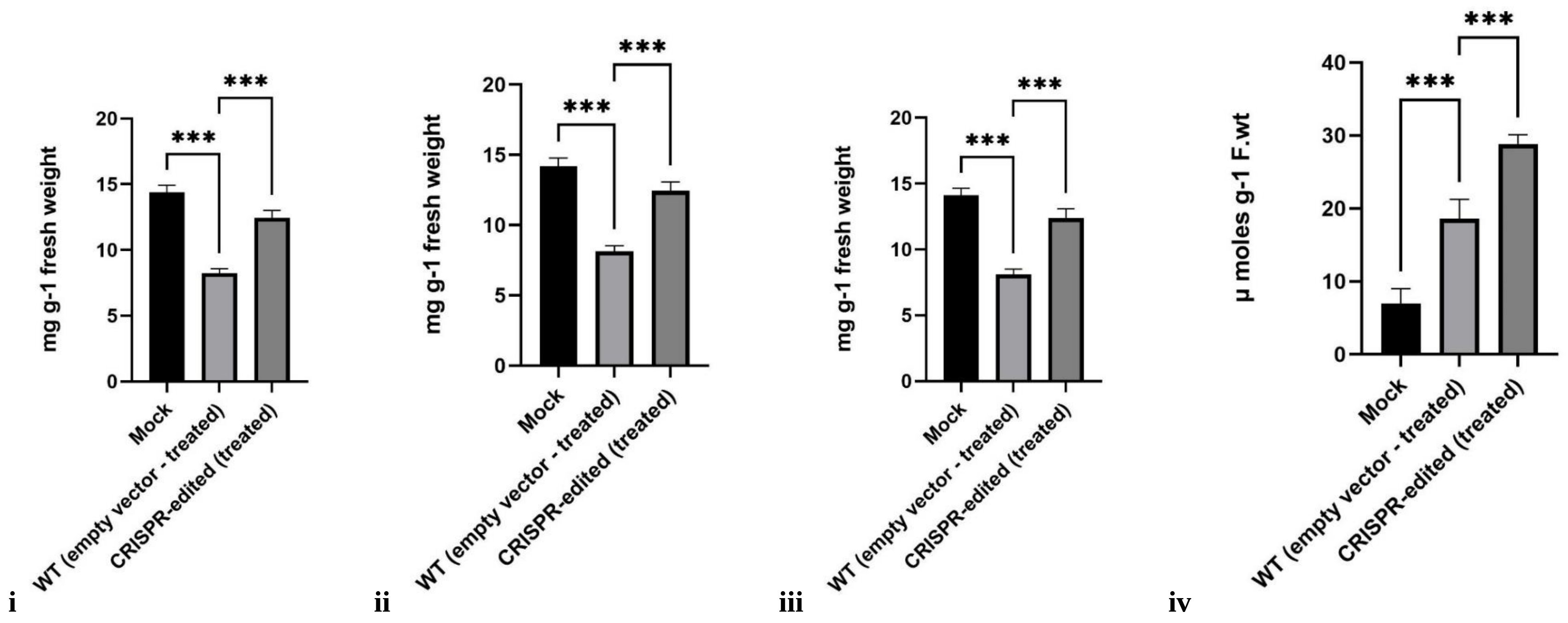

Fig S8 D. Physiological and biochemical parameters of salt-stressed plants. i. Chl a, ii. Chl b, iii. Ch a&b, iv. Proline content (Replicate-3).

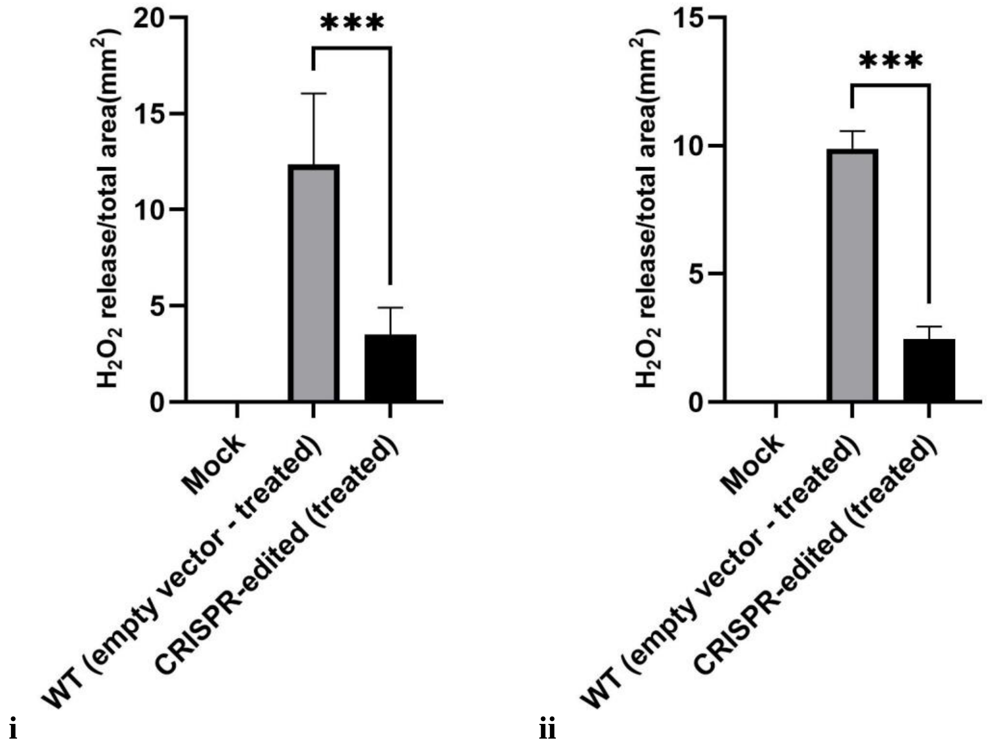

Fig S8 E. Quantification of ROS accumulation in *X. campetris* (BLS)-stressed plants. i. Replicate 2, ii. Replicate 3.

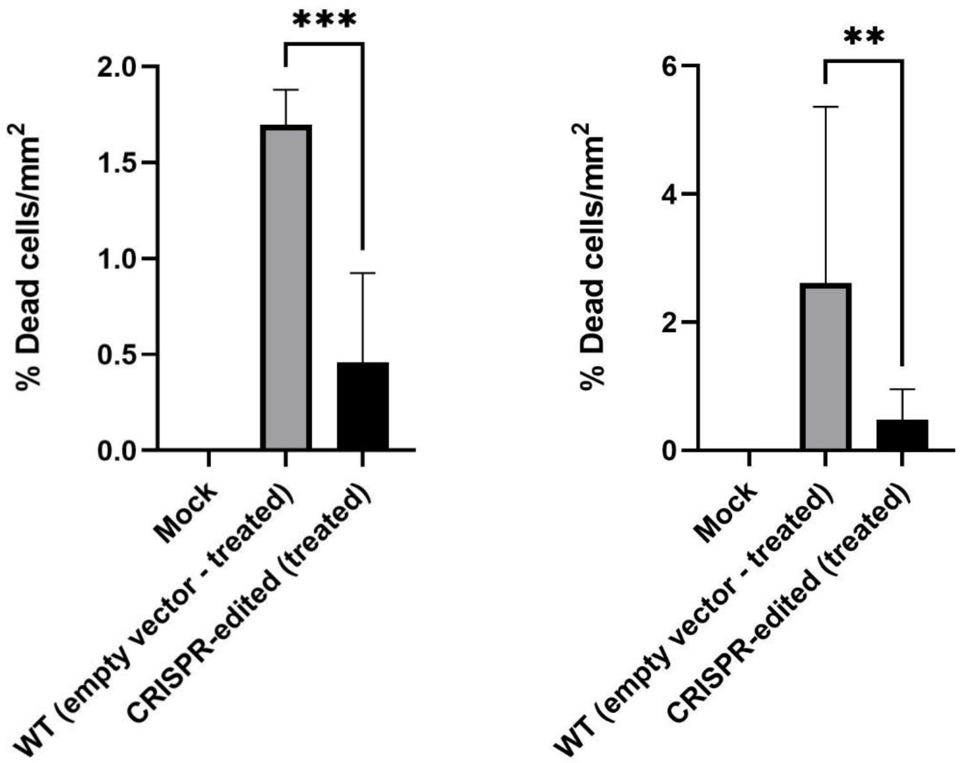

i ii

Fig S8 F. Quantification of cell death in *X. campetris* (BLS)-stressed plants. i. Replicate 2, ii. Replicate 3.

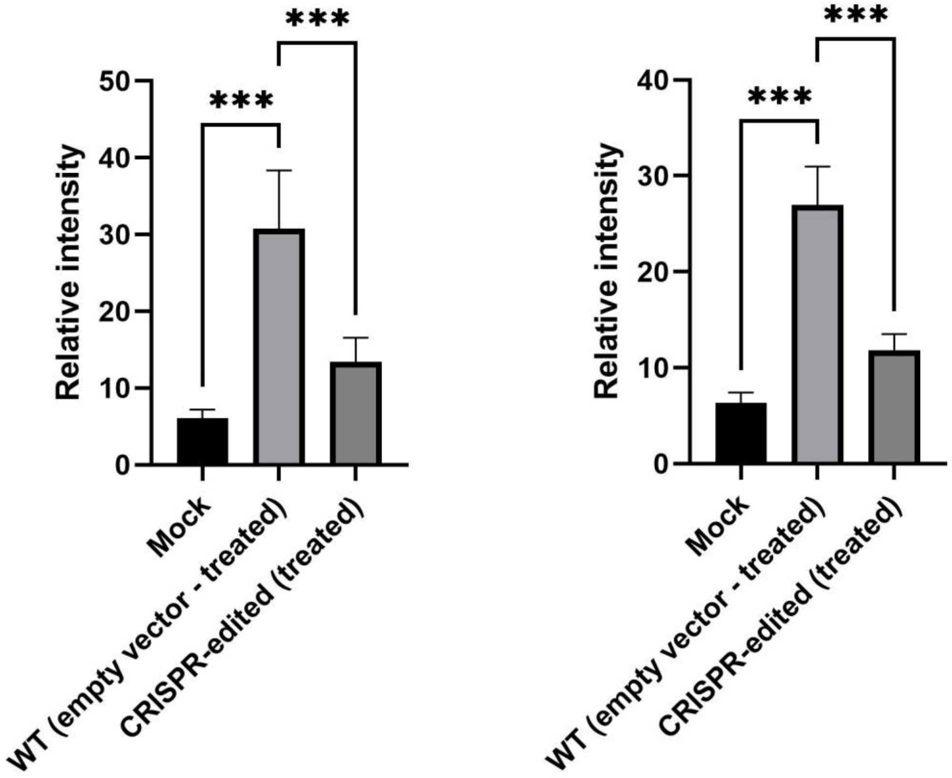

i ii

Fig S8 G. Quantification of ROS accumulation in *R. solanacearum* (Bacterial wilt)-stressed plants. i. Replicate 2, ii. Replicate 3.

A. Drought stressed plants analysed through Sanger Sequencing for CRISPR-edited events

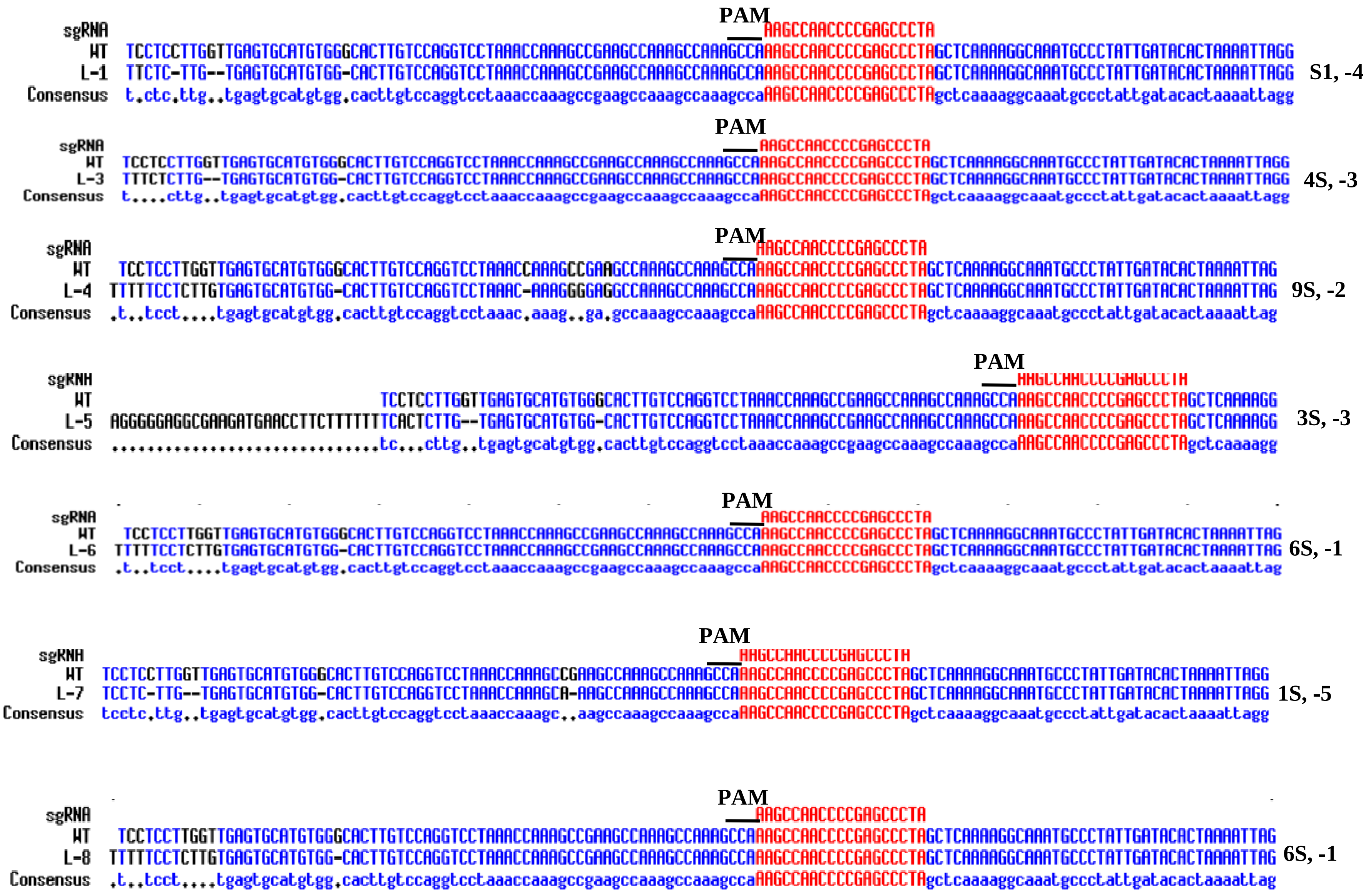

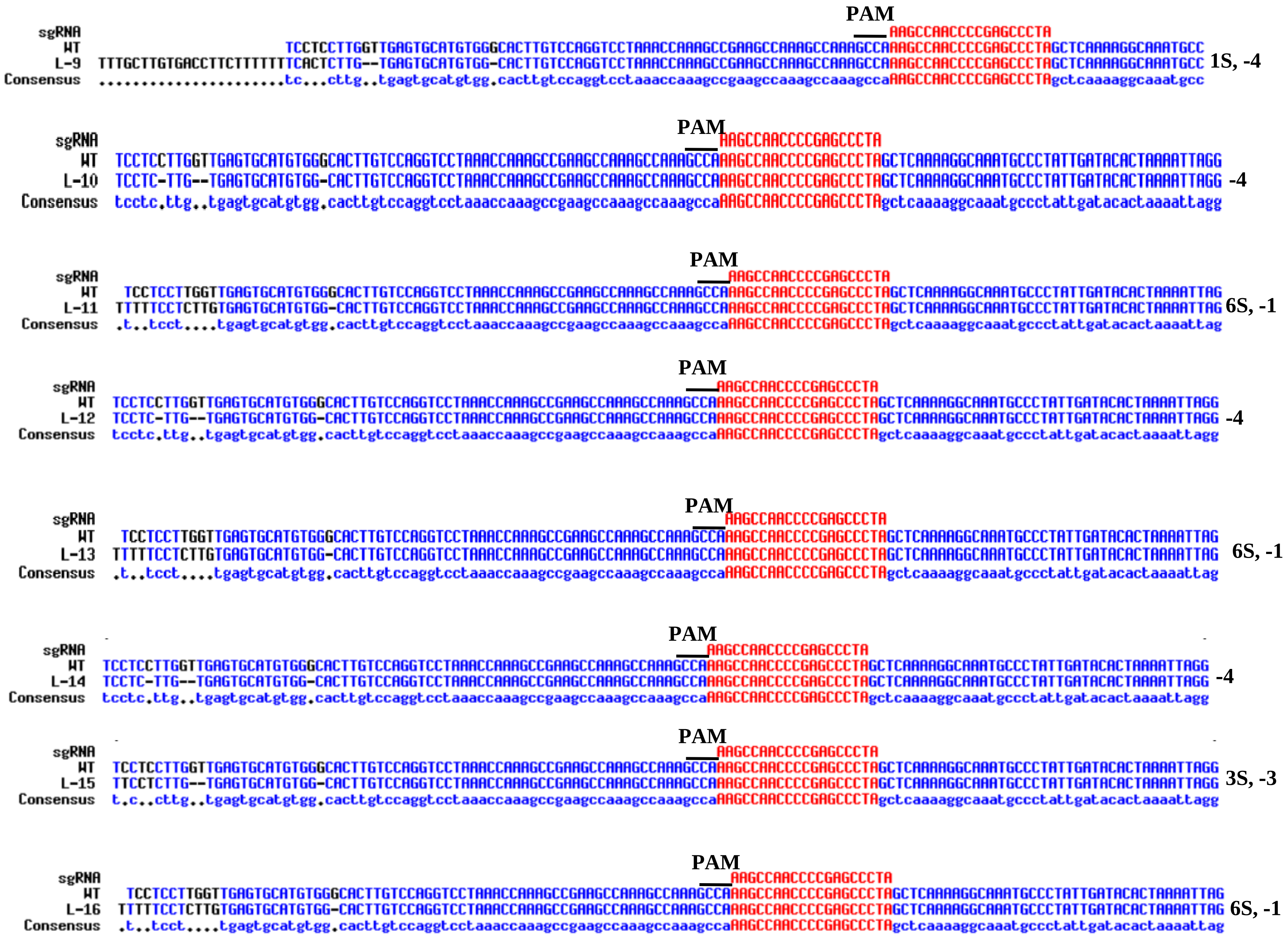
PAM
sgRNA AAGCCAACCCCGAGCCCTA
WT TCCTCCTTGGTTGAGTGCATGTGGGCACTTGTCCAGGTCCTAAACCAAAGCCGAAGCCAAAGCCAAAGCCAAAGCCAACCCCGAGCCCTAGCTCAAAAGGCAAATGCC
L-9 TTTGCTTGTGACCTTCTTTTTTTCACTCTTG--TGAGTGCATGTGG-CACTTGTCCAGGTCCTAAACCAAAGCCGAAGCCAAAGCCAAAGCCAAAGCCAACCCCGAGCCCTAGCTCAAAAGGCAAATGCC
Consensus ....tc...cttg..tgagtgcatgtgg.cacttgtccaggtcctaaaccaaagccgaagccaaagccaaagccaAAGCCAACCCCGAGCCCTAgctcaaaaggcaaatgcc
1S, -4
PAM
sgRNA AAGCCAACCCCGAGCCCTA
WT TCCTCCTTGGTTGAGTGCATGTGGGCACTTGTCCAGGTCCTAAACCAAAGCCGAAGCCAAAGCCAAAGCCAAAGCCAACCCCGAGCCCTAGCTCAAAAGGCAAATGCCCTATTGATACACTAAAATTAGG
L-10 TCCTC-TTG--TGAGTGCATGTGG-CACTTGTCCAGGTCCTAAACCAAAGCCGAAGCCAAAGCCAAAGCCAAAGCCAACCCCGAGCCCTAGCTCAAAAGGCAAATGCCCTATTGATACACTAAAATTAGG
Consensus tcctc.ttg..tgagtgcatgtgg.cacttgtccaggtcctaaaccaaagccgaagccaaagccaaagccaAAGCCAACCCCGAGCCCTAgctcaaaaggcaaatgccctattgatacactaaaattagg
-4
PAM
sgRNA AAGCCAACCCCGAGCCCTA
WT TCCTCCTTGGTTGAGTGCATGTGGGCACTTGTCCAGGTCCTAAACCAAAGCCGAAGCCAAAGCCAAAGCCAAAGCCAACCCCGAGCCCTAGCTCAAAAGGCAAATGCCCTATTGATACACTAAAATTAG
L-11 TTTTTCCTCTTGTGAGTGCATGTGG-CACTTGTCCAGGTCCTAAACCAAAGCCGAAGCCAAAGCCAAAGCCAAAGCCAACCCCGAGCCCTAGCTCAAAAGGCAAATGCCCTATTGATACACTAAAATTAG
Consensus .t..tcct....tgagtgcatgtgg.cacttgtccaggtcctaaaccaaagccgaagccaaagccaaagccaAAGCCAACCCCGAGCCCTAgctcaaaaggcaaatgccctattgatacactaaaattag
6S, -1
PAM
sgRNA AAGCCAACCCCGAGCCCTA
WT TCCTCCTTGGTTGAGTGCATGTGGGCACTTGTCCAGGTCCTAAACCAAAGCCGAAGCCAAAGCCAAAGCCAAAGCCAACCCCGAGCCCTAGCTCAAAAGGCAAATGCCCTATTGATACACTAAAATTAGG
L-12 TCCTC-TTG--TGAGTGCATGTGG-CACTTGTCCAGGTCCTAAACCAAAGCCGAAGCCAAAGCCAAAGCCAAAGCCAACCCCGAGCCCTAGCTCAAAAGGCAAATGCCCTATTGATACACTAAAATTAGG
Consensus tcctc.ttg..tgagtgcatgtgg.cacttgtccaggtcctaaaccaaagccgaagccaaagccaaagccaAAGCCAACCCCGAGCCCTAgctcaaaaggcaaatgccctattgatacactaaaattagg
-4
PAM
sgRNA AAGCCAACCCCGAGCCCTA
WT TCCTCCTTGGTTGAGTGCATGTGGGCACTTGTCCAGGTCCTAAACCAAAGCCGAAGCCAAAGCCAAAGCCAAAGCCAACCCCGAGCCCTAGCTCAAAAGGCAAATGCCCTATTGATACACTAAAATTAG
L-13 TTTTTCCTCTTGTGAGTGCATGTGG-CACTTGTCCAGGTCCTAAACCAAAGCCGAAGCCAAAGCCAAAGCCAAAGCCAACCCCGAGCCCTAGCTCAAAAGGCAAATGCCCTATTGATACACTAAAATTAG
Consensus .t..tcct....tgagtgcatgtgg.cacttgtccaggtcctaaaccaaagccgaagccaaagccaaagccaAAGCCAACCCCGAGCCCTAgctcaaaaggcaaatgccctattgatacactaaaattag
6S, -1
PAM
sgRNA AAGCCAACCCCGAGCCCTA
WT TCCTCCTTGGTTGAGTGCATGTGGGCACTTGTCCAGGTCCTAAACCAAAGCCGAAGCCAAAGCCAAAGCCAAAGCCAACCCCGAGCCCTAGCTCAAAAGGCAAATGCCCTATTGATACACTAAAATTAGG
L-14 TCCTC-TTG--TGAGTGCATGTGG-CACTTGTCCAGGTCCTAAACCAAAGCCGAAGCCAAAGCCAAAGCCAAAGCCAACCCCGAGCCCTAGCTCAAAAGGCAAATGCCCTATTGATACACTAAAATTAGG
Consensus tcctc.ttg..tgagtgcatgtgg.cacttgtccaggtcctaaaccaaagccgaagccaaagccaaagccaAAGCCAACCCCGAGCCCTAgctcaaaaggcaaatgccctattgatacactaaaattagg
-4
PAM
sgRNA AAGCCAACCCCGAGCCCTA
WT TCCTCCTTGGTTGAGTGCATGTGGGCACTTGTCCAGGTCCTAAACCAAAGCCGAAGCCAAAGCCAAAGCCAAAGCCAACCCCGAGCCCTAGCTCAAAAGGCAAATGCCCTATTGATACACTAAAATTAGG
L-15 TTCCTCTTG--TGAGTGCATGTGG-CACTTGTCCAGGTCCTAAACCAAAGCCGAAGCCAAAGCCAAAGCCAAAGCCAACCCCGAGCCCTAGCTCAAAAGGCAAATGCCCTATTGATACACTAAAATTAGG
Consensus t.c..cttg..tgagtgcatgtgg.cacttgtccaggtcctaaaccaaagccgaagccaaagccaaagccaAAGCCAACCCCGAGCCCTAgctcaaaaggcaaatgccctattgatacactaaaattagg
3S, -3
PAM
sgRNA AAGCCAACCCCGAGCCCTA
WT TCCTCCTTGGTTGAGTGCATGTGGGCACTTGTCCAGGTCCTAAACCAAAGCCGAAGCCAAAGCCAAAGCCAAAGCCAACCCCGAGCCCTAGCTCAAAAGGCAAATGCCCTATTGATACACTAAAATTAG
L-16 TTTTTCCTCTTGTGAGTGCATGTGG-CACTTGTCCAGGTCCTAAACCAAAGCCGAAGCCAAAGCCAAAGCCAAAGCCAACCCCGAGCCCTAGCTCAAAAGGCAAATGCCCTATTGATACACTAAAATTAG
Consensus .t..tcct....tgagtgcatgtgg.cacttgtccaggtcctaaaccaaagccgaagccaaagccaaagccaAAGCCAACCCCGAGCCCTAgctcaaaaggcaaatgccctattgatacactaaaattag
6S, -1

B. Salt stressed plants analysed through Sanger Sequencing for CRISPR-edited events

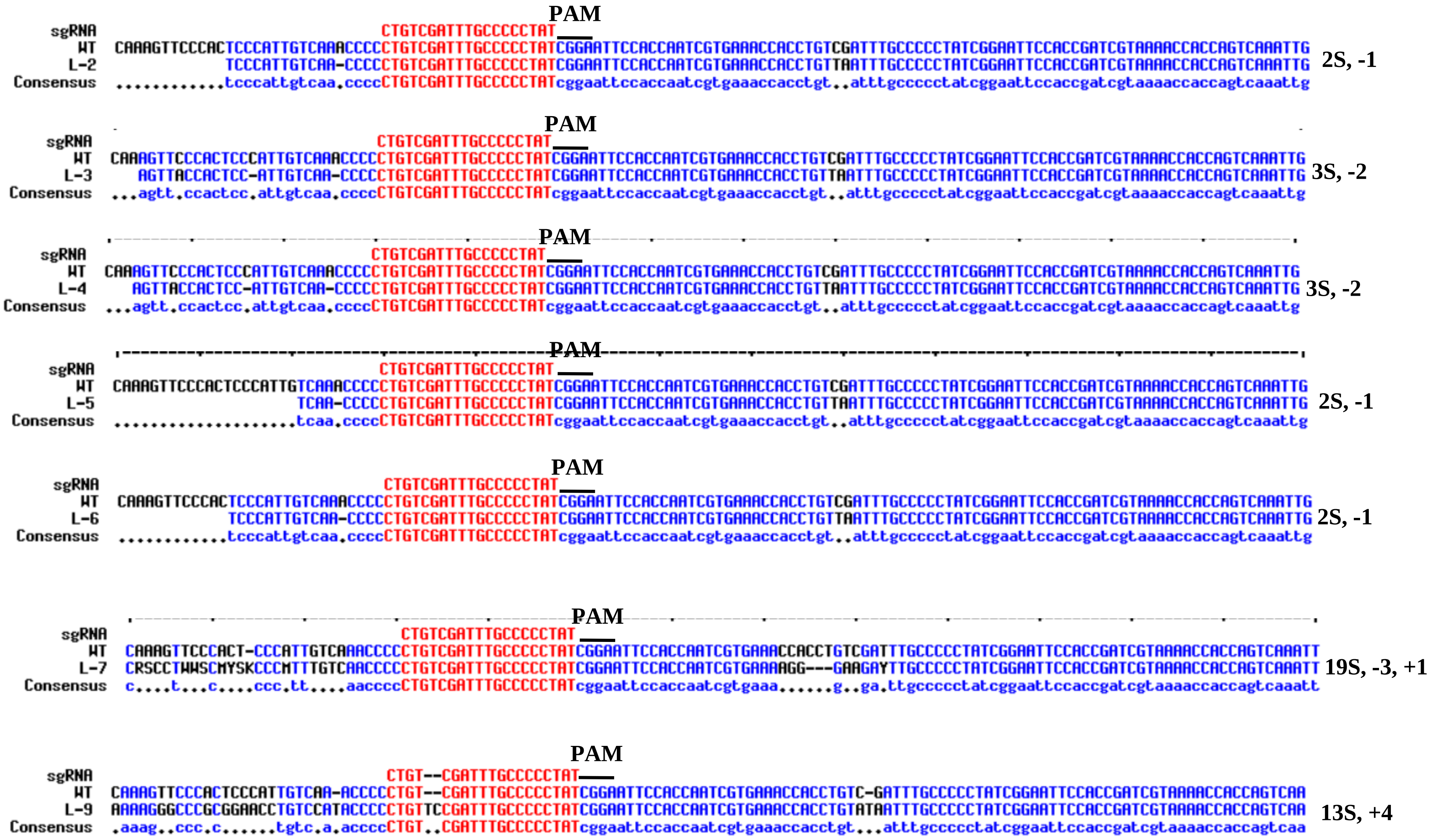

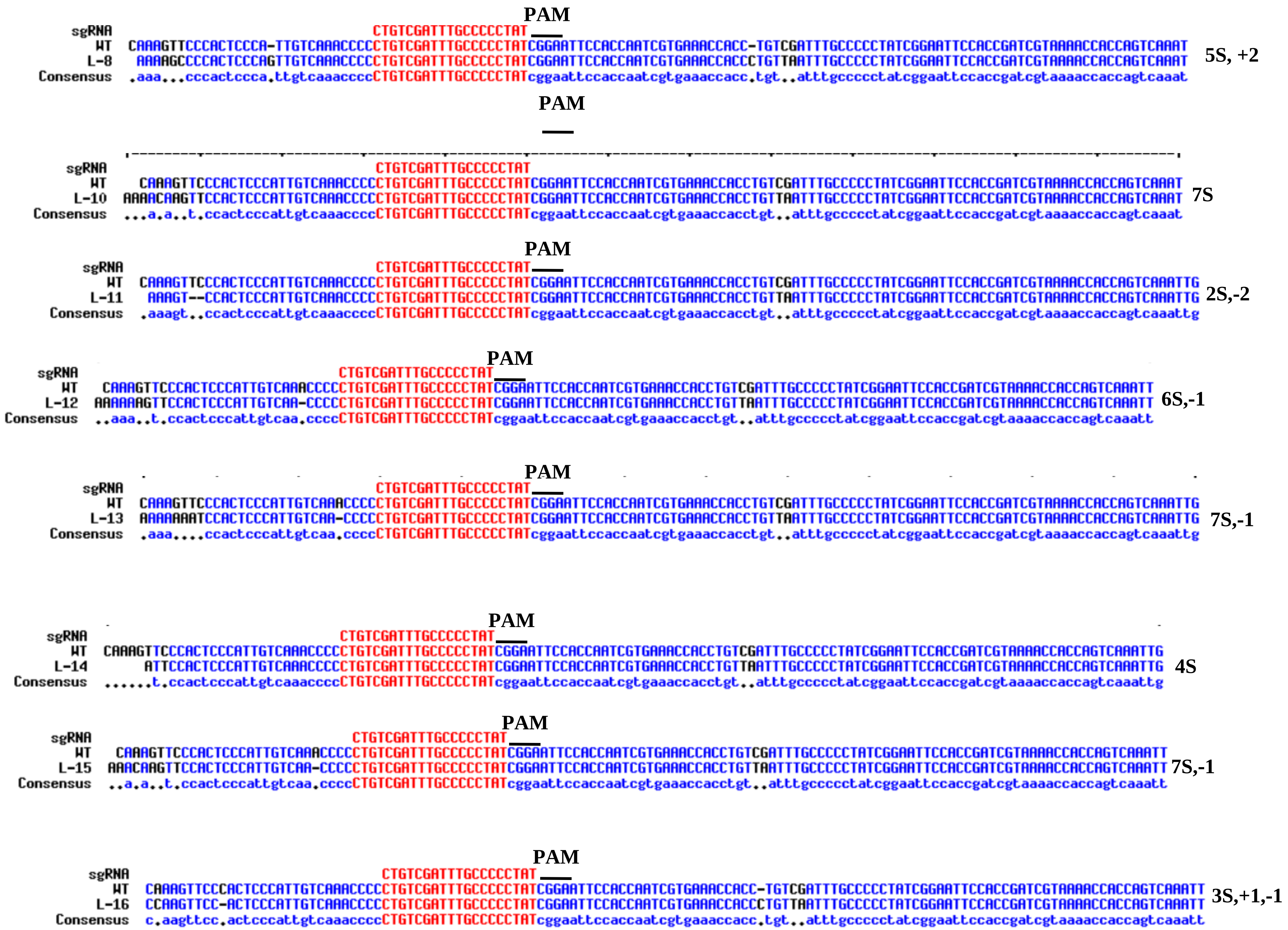
PAM
sgRNA CTGTCGATTTGCCCCCTAT
WT
L-8
Consensus
5S, +2
PAM
sgRNA CTGTCGATTTGCCCCCTAT
WT
L-10
Consensus
7S
PAM
sgRNA CTGTCGATTTGCCCCCTAT
WT
L-11
Consensus
2S,-2
PAM
sgRNA CTGTCGATTTGCCCCCTAT
WT
L-12
Consensus
6S,-1
PAM
sgRNA CTGTCGATTTGCCCCCTAT
WT
L-13
Consensus
7S,-1
PAM
sgRNA CTGTCGATTTGCCCCCTAT
WT
L-14
Consensus
4S
PAM
sgRNA CTGTCGATTTGCCCCCTAT
WT
L-15
Consensus
7S,-1
PAM
sgRNA CTGTCGATTTGCCCCCTAT
WT
L-16
Consensus
3S,+1,-1

C. *X. campestris* (BLS) stressed plants analysed through Sanger sequencing for CRSIPR-editing events (*SlHyPRP1)*

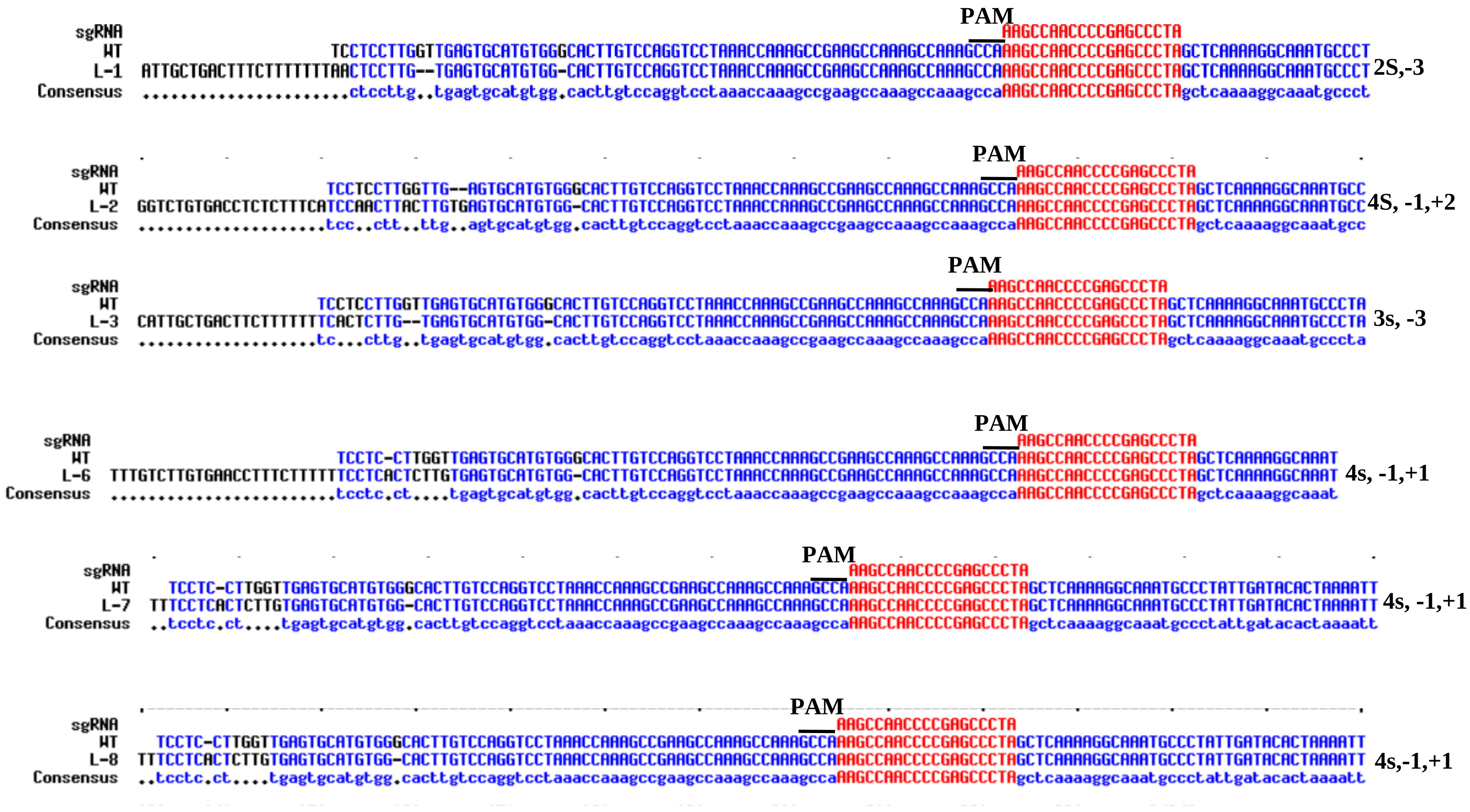

D. *X. campestris* (BLS) stressed plants analysed through Sanger sequencing for CRSIPR-editing events (*SlDEA1*)

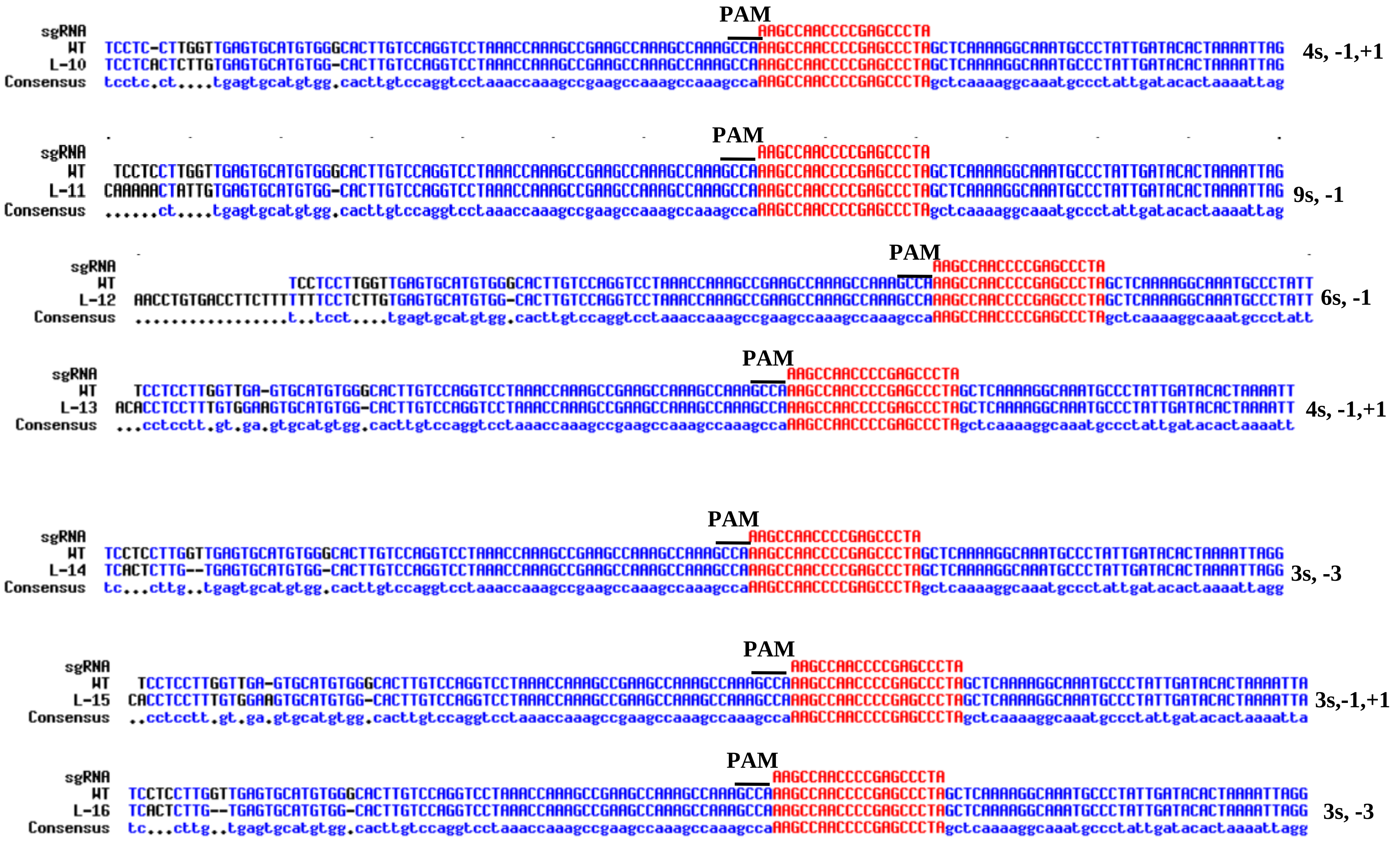

E. *R. solanacearum* (Bacterial wilt) stressed plants analysed through Sanger sequencing for CRSIPR-editing events

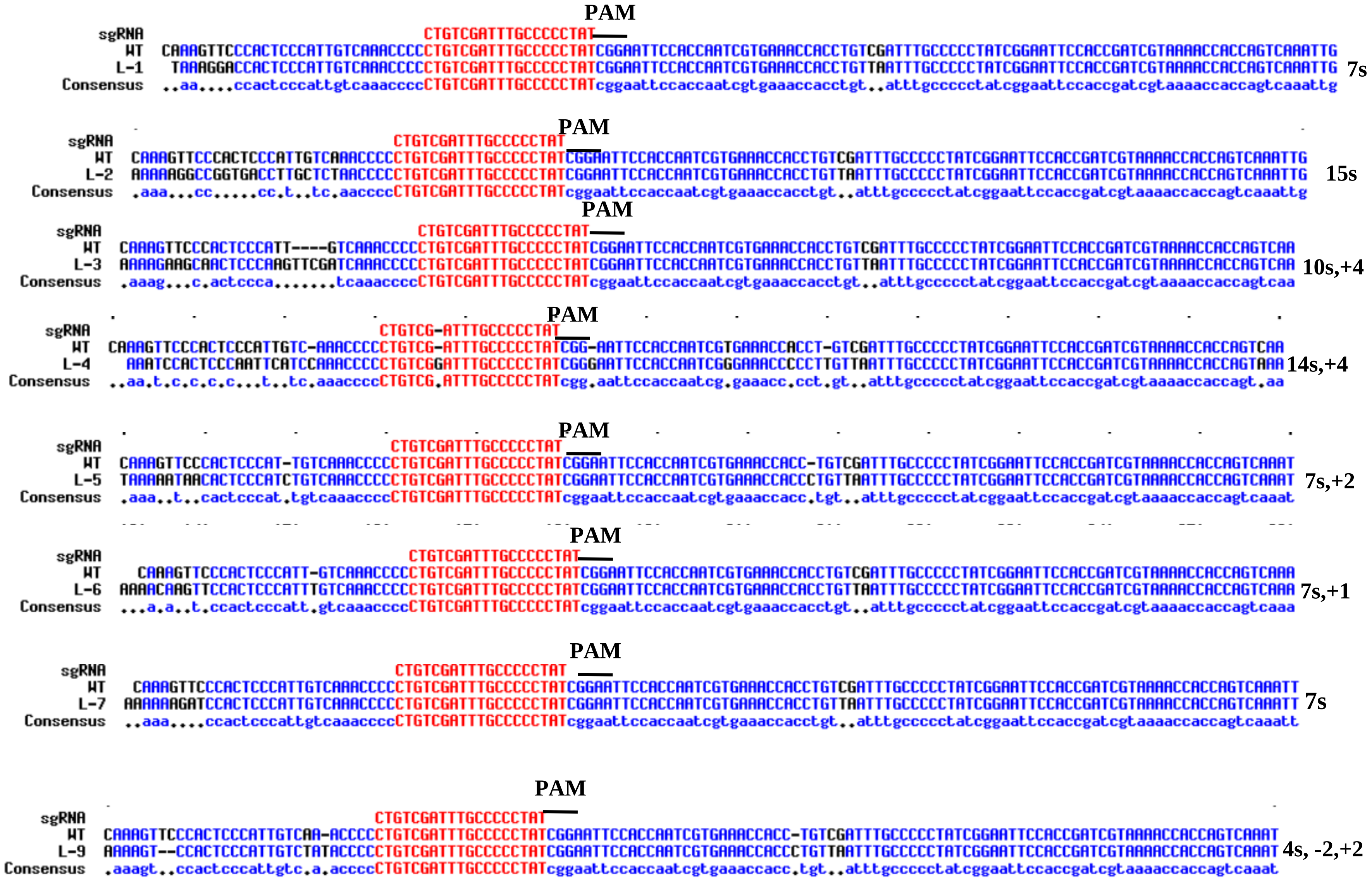

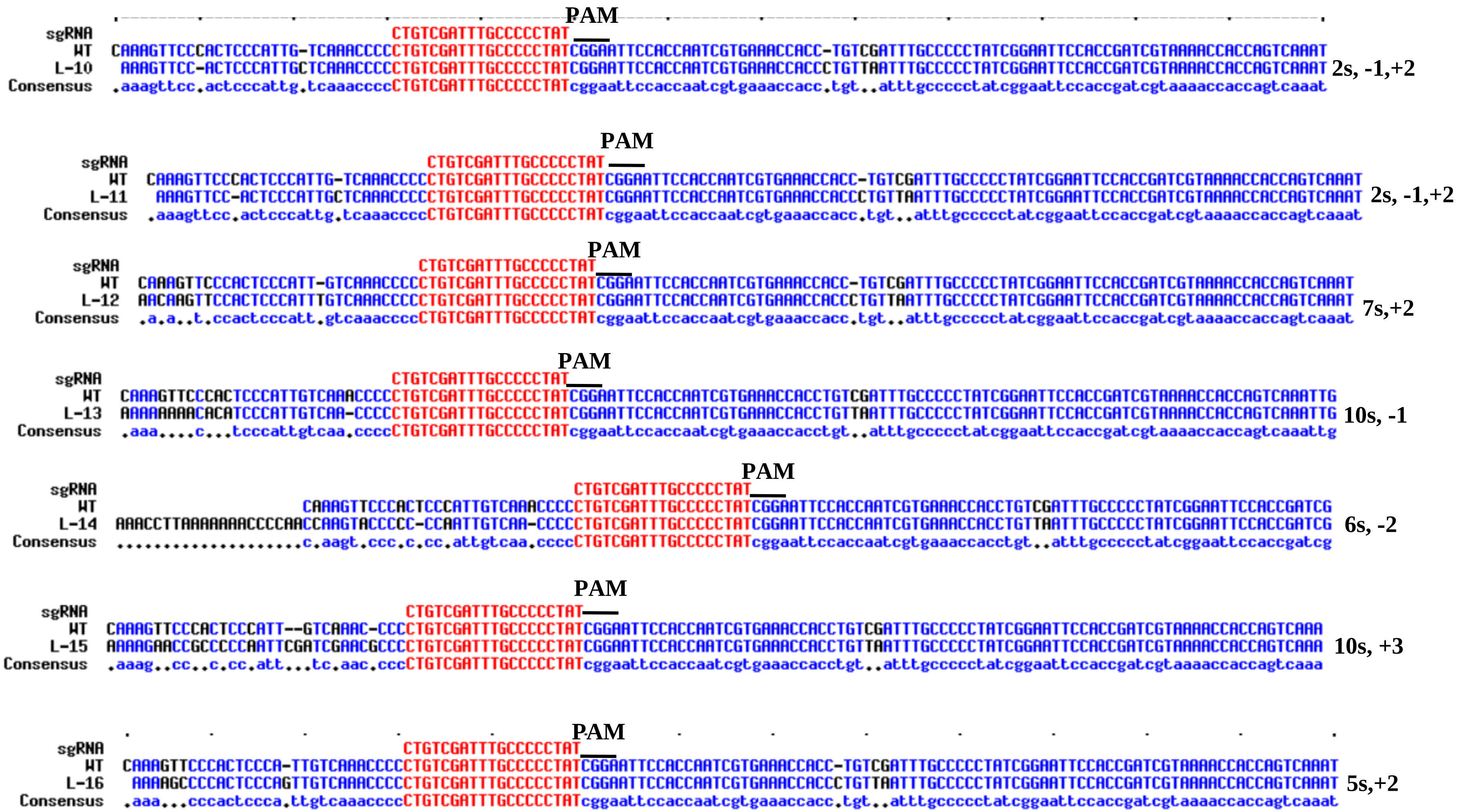

Fig S9. Confirmation of all CRISPR-editing events of transiently transformed leaves from *S.lycopersicum* L. cv Arka Vikas by Sanger sequencing. The Sanger sequence results were analyzed by Multalign tool.

A. Sanger sequencing alignment of single gene (*SlHyPRP1*) editing events in stable line (GEd0)

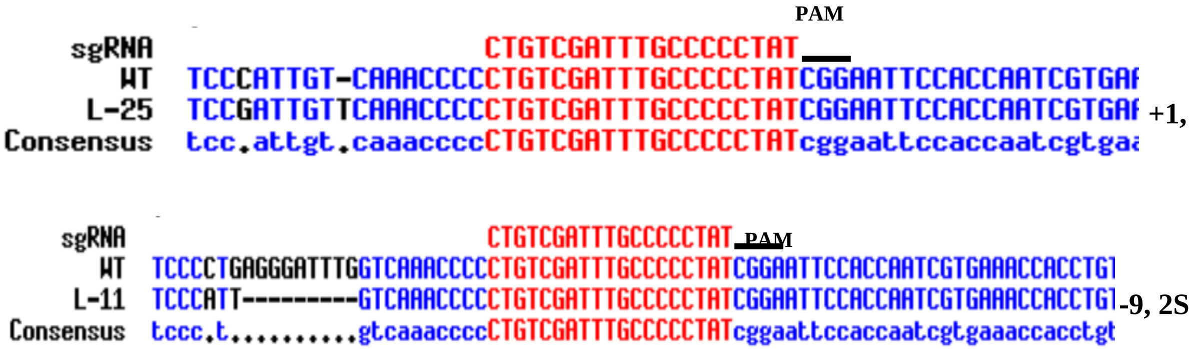

B. Sanger sequencing alignment of single gene (*SlDEA1*) editing events in stable line (GEd0)

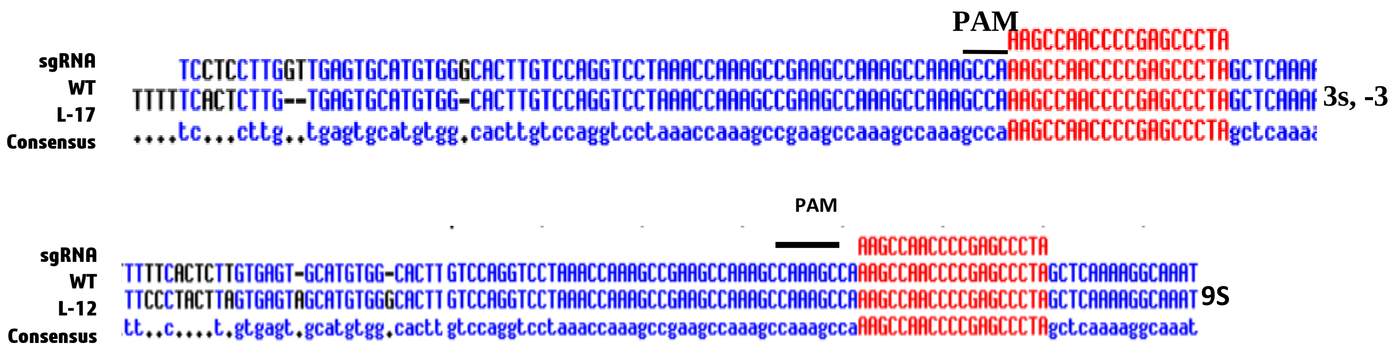

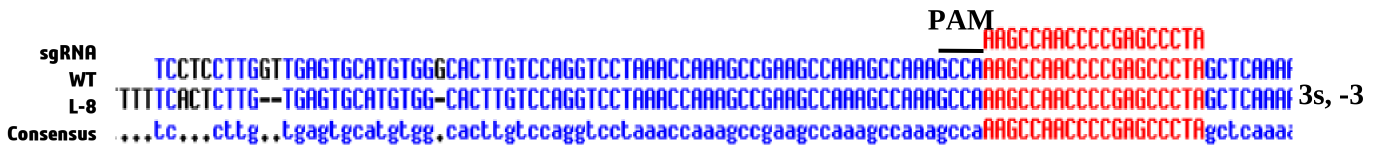

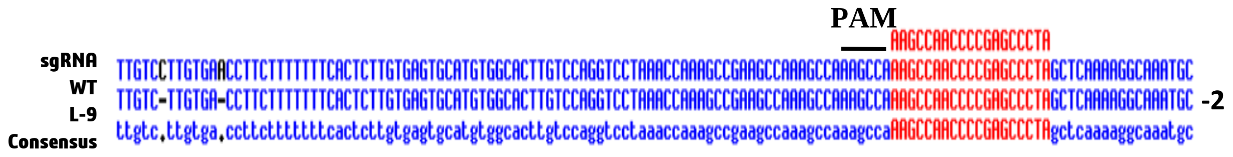

C. Sanger sequencing alignment of dual gene (*SlHyPRP1* and *SlDEA1*) editing events in stable line (GEd0)

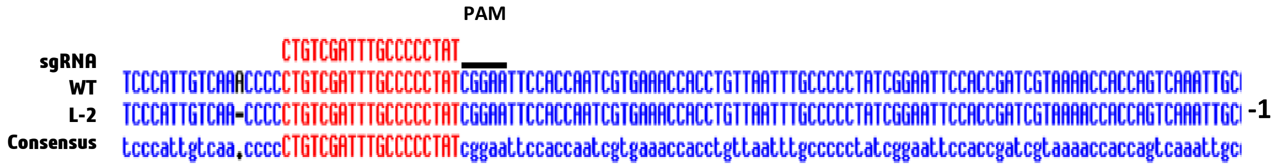

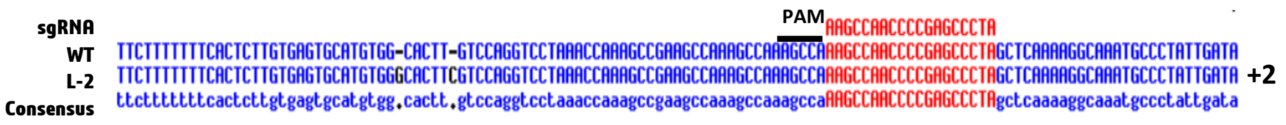

D. Sanger sequencing alignment of single gene (*SlHyPRP1*) editing events in stable line (GEd1)

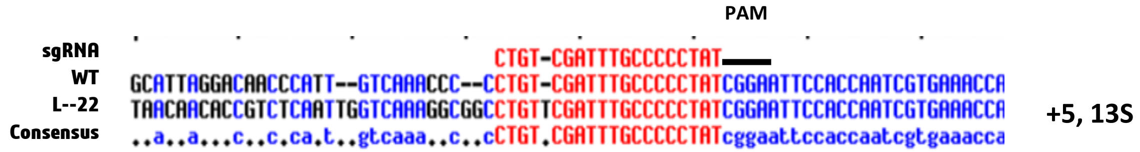

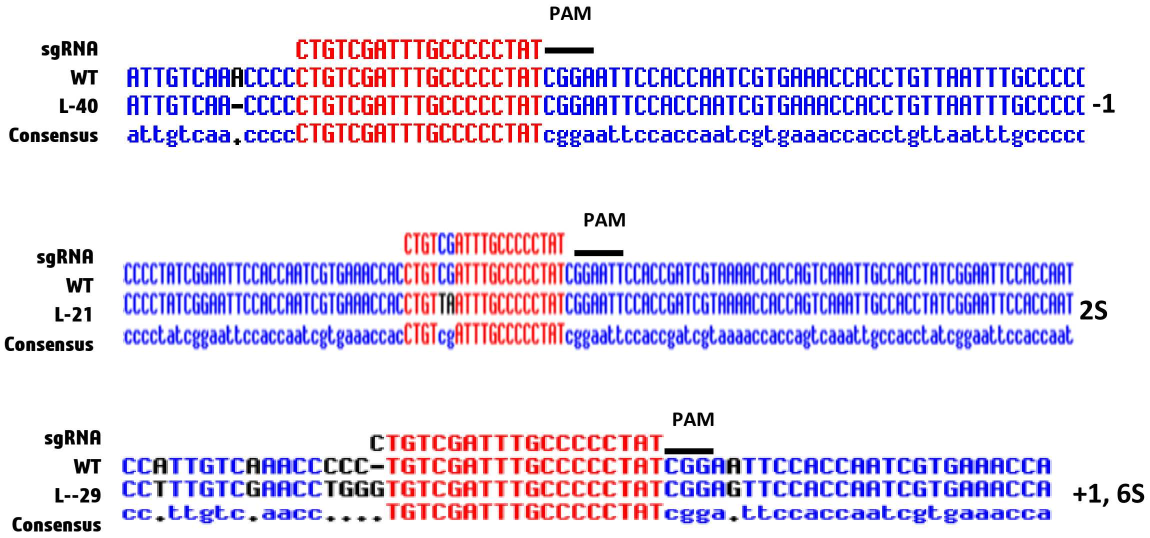

E. Sanger sequencing alignment of single gene (*SlDEA1*) editing events in stable line (GEd1)

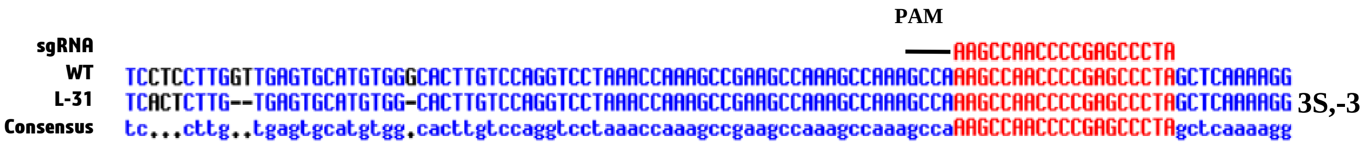

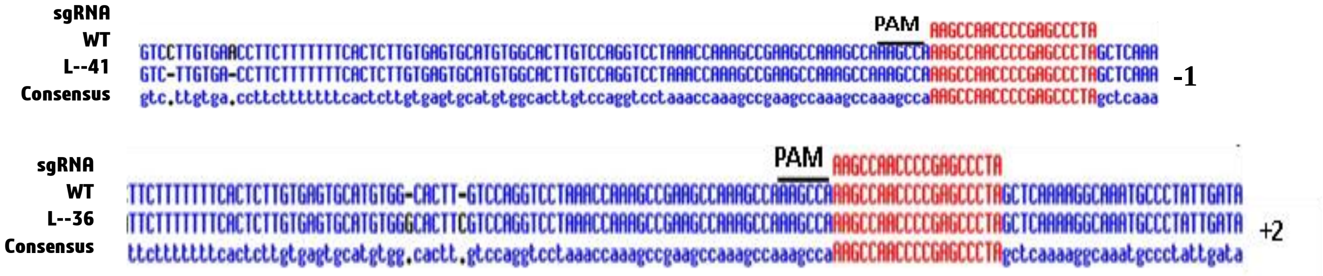

E. Raw PCR gel image showing amplification of 19 stable transformed lines

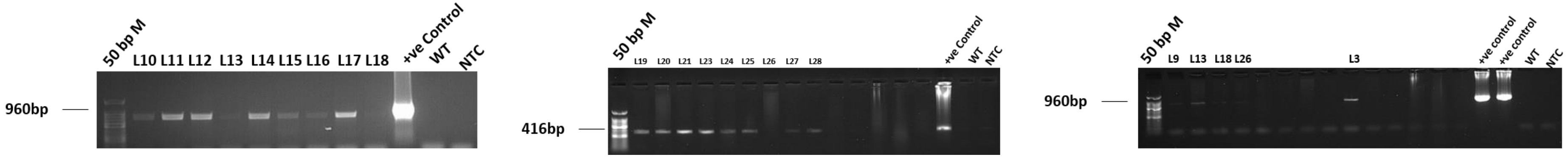

Fig S10. Sanger sequencing confirmation of the CRISPR editing events of stable lines from *S. lycopersicum* L. cv Arka Vikas. The direct PCR products were analyzed by Multalign.

2. Supplementary Tables

Table S1. List of primers used for the study (designed using Primer3 and VectorNTI software)

Primer name	Sequence (5'-3')
SlHyPRP1_F (gene specific primer)	AGCAGCTTCAAGTTCAACAAG
SlHyPRP1_R(gene specific primer)	GGCAACATGCCCAATTGAC
SlDEA1_F (gene specific primer)	GGGCATTTGCCTTTTGAGCTA
SlDEA1_R (gene specific primer)	CTCTTGTGAGTGCATGTGGC
EF1_F	GTCATTGGCCACGTCGACTC
EF1_R	TGTTCATCTCAGCGGCTTCC
Cas9_Fwd	CGGAACTAACTCTGTTGGATGGG
Cas9_Rvs	CCGATCTGAGCGAGGAGGTTAT
U6 promoter_Fwd	AGAAATCTCAAAATTCCGGCAGA
RNA Scaffold_Rvs	CCGACTCGGTGCCACTTTT
GUS-Fwd	AAACTTGTTCACCGACCACGGC
GUS-Rvs	CAACAAGTCGTTGCTACAGGCC
Solyc07g041180.2_Fwd	CAACTGGATCCGATTGGAGG

Solyc07g041180.2_Rvs	ACTGGACTGGATACCTGCAT
Solyc07g063250_Fwd	ATGAATTTACCCATAAGCCC
Solyc07g063250_Rvs	GGAACAGGAGAAGGAAGAAC

Table S2. Guide RNA designing of *SlHyPRP1* and *SlDEA1* using (CCTop) and CRISPR-P tools

Target gene	Target Sequence	PAM	Efficiency (%)	Specificity (%)
SlHyPRP1	CTGTCGATTTGCCCCCTAT	**CGG**	51.37	98.89
SlDEA1	TAGGGCTCGGGGTTGGCTT<br>AAGCCAACCCCGAGCCCTA<br>(Reverse compliment)	**TGG**<br>**ACC**	51.36	99.40

Table S3. Off-targets of *SlHyPRP1* and *SlDEA1* in transient and GEd0 CRELs

Target	Potential off-target	No. of mismatch	No. of plants tested	No. of mutations
Solyc07g041180 (for *SlHyPRP1*)	CCTTCCGAATTGCCCCCTCT	4	3	0
Solyc07g063250 (for *SlDEA1*)	AAGCCAAGCCCAAGCCCAAA	4	3	0

Table S4. Data on the determination of Chlorophyll content in drought-stressed seedlings

Sl. No.	Samples	Rep. 1			Rep. 2			Rep. 3		
		Chl. a	Chl. b	Chl. a+b	Chl. a	Chl. b	Chl. a+b	Chl. a	Chl. b	Chl. a+b
1	Mock	16.73	17.51	18.73	17.49	17.76	17.35	16.79	16.58	16.79
2	Mock	16.64	17.64	18.64	17.58	17.52	17.42	16.58	16.43	16.82
3	Mock	12.17	11.17	14.17	11.58	11.34	12.46	12.35	12.65	12.14
4	Mock	11.78	11.78	14.78	11.46	11.79	12.05	12.65	12.46	12.17
5	Mock	13.17	12.47	13.47	12.43	13.05	13.64	12.65	12.46	12.99
6	Mock	13.11	12.81	14.1	12.68	12.35	13.56	13.61	13.71	14.01
7	Mock	12.87	12.64	14.87	12.56	12.58	12.43	12.58	13.04	13.16
8	Mock	13.1	12.7	14.45	13.14	13.05	12.59	12.47	13.64	13.28
9	Mock	17.32	19.77	16.32	16.35	17.28	17.24	16.37	17.69	17.85
10	Mock	17.81	19.81	16.81	18.09	17.64	16.48	17.64	18.18	18.43
11	Mock	18.95	18.61	17.55	15.47	17.35	17.86	18.67	18.94	17.49
12	Mock	18.89	18.73	17.89	16.89	17.68	17.49	18.76	17.61	18.57
13	Mock	16.71	16.71	16.54	15.89	16.45	16.79	16.34	18.67	19.47
14	Mock	16.85	16.68	16.63	17.49	17.59	17.64	17.81	16.76	16.28
15	Mock	19.1	19	18.1	17.68	17.49	17.29	17.29	18.49	18.24
16	Mock	19.12	19.15	18.12	19.24	18.67	18.64	18.29	18.67	18.16
17	WT-Treated	8.25	5.75	8.87	8.69	8.76	9.58	9.36	8.15	7.98
18	WT-Treated	8.68	5.68	8.69	8.56	8.46	8.25	9.67	10.58	9.36

19	WT-Treated	6.23	5.13	6.33	7.65	6.31	6.24	6.19	6.08	7.1
20	WT-Treated	6.27	5.17	6.39	6.44	8.16	8.04	8.19	7.35	6.46
21	WT-Treated	4.86	4.98	4.97	5.67	5.19	6.34	6.31	7.56	7.42
22	WT-Treated	4.92	4.89	4.92	7.25	5.21	4.89	4.62	4.38	4.63
23	WT-Treated	3.14	3.04	3.14	5.29	4.35	4.18	4.24	5.26	5.35
24	WT-Treated	3.25	3.12	3.25	4.56	5.26	5.14	4.85	4.36	4.87
25	WT-Treated	5.15	5.08	5.18	6.25	5.46	5.29	5.14	5.13	5.43
26	WT-Treated	5.19	5.11	5.2	6.31	4.65	5.24	5.16	5.24	5.31
27	WT-Treated	4.78	4.32	4.89	4.19	4.35	4.28	4.43	5.02	4.66
28	WT-Treated	4.8	4.36	4.93	4.27	4.15	5.34	5.28	6.34	4.28
29	WT-Treated	3.85	2.75	3.85	4.25	3.66	3.78	3.35	4.52	4.61
30	WT-Treated	3.9	2.67	3.94	2.47	3.52	3.94	3.58	4.37	5.26
31	WT-Treated	3.45	3.36	3.57	4.39	4.01	4.03	4	3.59	4.24
32	WT-Treated	3.56	3.39	3.66	4.11	3.67	3.44	3.54	3.66	3.12
33	CRISPR-Treated	11.64	10.59	11.78	10.46	10.75	9.61	9.36	11	11.21
34	CRISPR-Treated	10.95	10.63	11.21	10.14	10.38	9.36	9.48	9.55	8.63
35	CRISPR-Treated	11.5	8.68	11.15	10.35	9.26	9.45	11.46	11.02	9.26
36	CRISPR-Treated	12.14	8.71	12.23	8.64	8.74	9.66	10.49	11.01	9.32
37	CRISPR-Treated	16.23	14.88	16.12	15.62	14.52	14.46	14.28	14.61	14.59
38	CRISPR-Treated	17.1	14.91	17.1	14.56	14.35	14.35	13.43	13.85	13.69
39	CRISPR-Treated	11.62	11.51	11.72	11.46	12.31	10.64	11.42	11.75	11.02
40	CRISPR-Treated	11.68	11.53	11.71	11.54	11.35	10.25	11.45	11.37	11.62
41	CRISPR-Treated	15.82	15.01	15.82	16.21	15.94	15.76	15.04	15.26	16.14
42	CRISPR-Treated	15.89	15.11	15.85	15.26	15.34	15.24	15.84	14.88	14.76
43	CRISPR-Treated	16.76	16.36	16.75	15.42	14.25	15.63	16.35	16.55	15.64
44	CRISPR-Treated	16.71	16.56	16.34	15.62	15.57	16.27	16.49	15.26	14.35
45	CRISPR-Treated	16.15	13.95	16.47	16.35	16.14	16.84	16.84	16.79	14.25
46	CRISPR-Treated	16.24	13.89	16.34	13.52	16.32	16.94	16.41	13.58	14.25
47	CRISPR-Treated	10.15	9.17	10.95	11.32	10.58	10.46	9.88	10.03	10.35
48	CRISPR-Treated	11.45	9.2	11.35	11.48	11.64	11.76	12	10.79	11.14

Table S5. Data on the determination of Proline content of drought-stressed seedlings

Sl. No.	Samples	Rep1	Rep2	Rep3
		Proline content uM	Proline content uM	Proline content uM
1	MOCK	4.5	4.59	5.13
2	MOCK	4.98	4.96	5.24
3	MOCK	4.25	4.26	4.86
4	MOCK	4.43	4.98	4.71
5	MOCK	5.59	5.14	5.13
6	MOCK	5.71	5.35	5.35
7	MOCK	6.10	4.85	5.26
8	MOCK	6.15	4.98	5.46
9	MOCK	5.53	5.59	5.26
10	MOCK	5.65	5.53	5.59
11	MOCK	6.39	4.64	5.71
12	MOCK	6.57	4.85	6.26
13	MOCK	6.02	4.97	5.53
14	MOCK	6.26	4.98	5.62
15	MOCK	4.43	4.99	4.85
16	MOCK	4.68	4.98	4.99
17	WT-Treated	11.36	10.94	8.75
18	WT-Treated	11.45	10.88	10.80
19	WT-Treated	7.30	8.56	8.86
20	WT-Treated	7.54	8.75	8.87
21	WT-Treated	10.84	11.59	11.48
22	WT-Treated	10.71	11.62	8.45
23	WT-Treated	7.48	11.99	11.89
24	WT-Treated	8.09	11.97	11.89
25	WT-Treated	12	11.23	11.59
26	WT-Treated	12.07	11.62	10.94

27	WT-Treated	12.548	12.18	13.32
28	WT-Treated	12.73	12.15	12.15
29	WT-Treated	12.06	12.34	12.53
30	WT-Treated	12.18	12.40	12.73
31	WT-Treated	9.317	10.71	10.71
32	WT-Treated	9.378	10.91	10.91
33	CRISPR-Treated	17.06	15.46	17.06
34	CRISPR-Treated	17.24	15.89	17.24
35	CRISPR-Treated	13.52	15.25	15.99
36	CRISPR-Treated	13.70	15.25	15.99
37	CRISPR-Treated	23.58	21.62	20.64
38	CRISPR-Treated	23.64	24.36	22.53
39	CRISPR-Treated	20.47	17.56	17.84
40	CRISPR-Treated	20.53	17.65	3.986
41	CRISPR-Treated	20.84	25.71	25.53
42	CRISPR-Treated	20.90	25.53	25.71
43	CRISPR-Treated	23.70	28.56	24.36
44	CRISPR-Treated	23.67	28.58	25.71
45	CRISPR-Treated	23.70	23.90	28.12
46	CRISPR-Treated	23.89	24	28.41
47	CRISPR-Treated	18.524	16.65	17.46
48	CRISPR-Treated	18.768	16.78	17.16

Table S6. Data on the determination of Chlorophyll content of salt-stressed seedlings

Sl. No.	Samples	Rep.1			Rep.2			Rep.3		
		Chl. a	Chl. b	Chl. a+b	Chl. a	Chl. b	Chl. a+b	Chl. a	Chl. b	Chl. a+b
1	Mock	14.173	14.173	15.04	14.21	14.11	15.15	14.39	14.65	14.23
2	Mock	13.92	13.92	14.92	13.16	14.28	15.35	14.05	13.75	13.35
3	Mock	14.9	15.39	14.93	14.36	13.56	14.98	14.79	14.83	14.21
4	Mock	13.087	14.56	14.76	13.68	13.46	14.59	13.58	14.66	14.61

5	Mock	13.9	14.45	14.59	14.88	14.16	15.01	14.96	15.06	15
6	Mock	13.602	13.56	13.56	13.24	13.6	13.67	13.421	13.31	13.03
7	Mock	14.54	14.11	14.45	14.34	14.28	14.77	14.2	14.12	14.5
8	Mock	14.295	13.95	13.95	14.115	14.22	13.34	14.26	13.92	13.88
9	Mock	14.24	14.49	13.98	14.36	14.51	13.31	14.26	13.43	14.07
10	Mock	13.89	14.05	14.21	13.63	14.18	14.36	14.14	14.26	13.67
11	Mock	13.19	13.67	14.87	13.17	13.52	14.29	13.59	13.11	13.76
12	Mock	14.87	14.78	15.08	14.73	14.92	14.16	15.23	14.63	14.49
13	Mock	14.98	13.98	15.78	14.84	13.85	14.99	14.76	14.57	13.86
14	Mock	14.95	13.97	15.98	14.86	13.73	14.99	14.6	13.66	13.65
15	Mock	14.68	14.27	14.89	14.47	14.42	14.28	14.99	13.93	14.58
16	Mock	14.76	14.172	15.12	15.24	15.31	14.86	14.84	14.87	14.79
17	WT-Treated	8.75	8.34	8.54	8.57	8.99	8.7	7.95	8.69	8.88
18	WT-Treated	8.78	8.48	8.76	8.36	8.71	8.47	7.86	8.54	8.25
19	WT-Treated	7.67	7.98	7.87	8	8.14	7.67	7.84	7.98	7.77
20	WT-Treated	7.88	7.56	7.45	7.46	7.69	7.35	8.1	7.48	7.69
21	WT-Treated	8.01	7.98	7.85	8.24	8.31	8.32	7.86	7.74	7.62
22	WT-Treated	8.34	8.12	8.29	8.47	7.49	8.55	8.51	7.44	7.67
23	WT-Treated	8.44	8.34	8.54	8.34	8.34	7.96	8.53	8.14	8.64
24	WT-Treated	8.78	8.68	8.78	8.63	8.79	8.67	8.57	8.44	8.24
25	WT-Treated	8.22	8.76	8.69	8.59	8.53	8.75	8.68	8.37	7.88
26	WT-Treated	8.525	8.64	8.7	8.47	8.37	8.29	8.3	8.17	8.29
27	WT-Treated	7.98	7.87	8.01	7.86	7.92	7.82	8.24	8.4	7.8
28	WT-Treated	7.98	7.84	7.9	7.86	7.44	7.68	7.58	7.95	7.76
29	WT-Treated	8.01	8.01	8.12	8.64	8.45	8.36	8.47	8.15	8.16
30	WT-Treated	8.48	8.38	8.67	8.34	8.53	8.35	8.69	8.49	8.34
31	WT-Treated	8.43	8.44	8.4	8.79	8.47	8.61	8.35	8.47	8.38
32	WT-Treated	8.89	8.94	8.56	8.47	8.68	8.31	8.34	7.94	8.49
33	CRISPR-Treated	12.198	13.12	13.1	12.79	12.86	13.68	12.94	12.56	12.49
34	CRISPR-Treated	12.135	12.96	12.76	12.79	11.96	12.56	12.36	12.48	12.75

35	CRISPR-Treated	11.93	12.89	12.91	11.96	12	12.02	12.34	12.56	12.49
36	CRISPR-Treated	11.23	11.82	11.86	12.34	12.01	12.16	12.18	12.09	11.68
37	CRISPR-Treated	11.63	11.89	11.9	11.25	11.37	11.67	11.69	11.48	11.66
38	CRISPR-Treated	12.03	12.32	12.16	12.58	12.79	12.59	12.76	12.49	12.45
39	CRISPR-Treated	12.78	12.89	12.1	11.31	11.49	11.76	12.65	12.19	12.46
40	CRISPR-Treated	12	12.1	12.23	12.25	12.26	12.17	12.05	12.41	12.32
41	CRISPR-Treated	12.89	12.65	12.78	12.13	12.08	12.46	12.07	11.86	11.55
42	CRISPR-Treated	12.13	12.34	12.56	12.48	12.49	12.03	11.67	11.48	11.34
43	CRISPR-Treated	13.1	13.15	13.71	13.14	13.59	12.46	13.67	13.54	13.67
44	CRISPR-Treated	12.8	12.82	12.45	12.56	12.48	12.67	12.06	11.97	11.68
45	CRISPR-Treated	12.76	12.89	12.34	12.46	12.96	12.48	13.02	13.76	13.54
46	CRISPR-Treated	13.97	12.97	13.37	13.49	12.99	12.75	12.48	12.76	12.48
47	CRISPR-Treated	12.98	12.85	13.1	12.45	12.37	12.79	13.15	13.14	13.26
48	CRISPR-Treated	12.87	12.89	13.25	12.49	12.74	12.28	12.19	12.2	12.43

Table S7. Data on the determination of Proline content of salt stress seedlings

Sl. No.	Samples	Rep1	Rep2	Rep3
		**Proline content uM**	**Proline content uM**	**Proline content uM**
1	MOCK	8.82	9.65	8.46
2	MOCK	8.90	9.64	8.52
3	MOCK	4.5	5.59	5.69
4	MOCK	4.75	5.65	5.66
5	MOCK	6.51	6.26	6.88
6	MOCK	6.59	6.35	6.87
7	MOCK	7.24	8.22	8.05
8	MOCK	8.21	9.96	11.43
9	MOCK	5.29	5.72	5.69
10	MOCK	5.35	5.83	5.72
11	MOCK	4.25	4.27	4.59

12	MOCK	4.37	4.379	4.263
13	MOCK	15.59	17.12	15.79
14	MOCK	15.90	15.15	15.79
15	MOCK	15.23	16.15	15.23
16	MOCK	15.41	16.28	15.64
17	WT-Treated	20.78	21.52	18.35
18	WT-Treated	20.90	21.53	18.94
19	WT-Treated	14.62	15.79	16.11
20	WT-Treated	14.74	15.80	16.05
21	WT-Treated	14.68	16.06	17.45
22	WT-Treated	14.80	16.08	19.86
23	WT-Treated	16.26	20.63	20.01
24	WT-Treated	16.39	20.82	20.05
25	WT-Treated	21.32	19.67	20.10
26	WT-Treated	21.45	19.70	20.03
27	WT-Treated	21.89	19.95	19.03
28	WT-Treated	20.01	18.23	19.89
29	WT-Treated	19.09	19.32	18.21
30	WT-Treated	20.21	18.41	19.76
31	WT-Treated	19.20	19.24	19.39
32	WT-Treated	19.39	19.68	19.86
33	CRISPR-Treated	30.04	29.41	29.21
34	CRISPR-Treated	30.10	29.63	29.35
35	CRISPR-Treated	26.45	27.85	28.41
36	CRISPR-Treated	26.63	28.08	28.56
37	CRISPR-Treated	31.93	30.01	29.88
38	CRISPR-Treated	32.60	30.11	30
39	CRISPR-Treated	28.28	28.85	29.65
40	CRISPR-Treated	28.89	28.96	29.86
41	CRISPR-Treated	27.67	28.65	29.75
42	CRISPR-Treated	27.85	28.74	28.64

43	CRISPR-Treated	29.5	28.85	29.79
44	CRISPR-Treated	29.68	28.96	29.89
45	CRISPR-Treated	29.87	28.98	29.98
47	CRISPR-Treated	29.78	29.75	29.87
48	CRISPR-Treated	28.97	28.67	29.87

Table S8. DAB assay raw data for *X. campestris* stress

Sl. No.	Samples	Rep.1			Rep.2			Rep.3		
		total area	infected area	% of infected area	total area	infected area	% of infected area	total area	infected area	% of infected area
**1**	MOCK	**0**	**0**	**0**	**0**	**0**	**0**	**0**	**0**	**0**
**2**	WT-Treated	24.55	2.76	11.24	24.463	2.59	9.684	24.59	2.89	11.65
**3**	WT-Treated	24.76	2.22	8.96	24.63	2.63	9.856	25.61	2.77	9.05
**4**	WT-Treated	24.87	2.44	9.88	25.41	2.74	9.624	26.46	3.05	10.12
**5**	WT-Treated	35.74	5.64	15.78	25.61	2.16	9.596	25.14	2.66	9.842
**6**	WT-Treated	35.72	5.61	15.70	24.763	3.51	10.61	28.56	2.94	10.85
**7**	WT-Treated	35.66	5.52	15.47	26.46	2.99	10.67	26.74	2.64	9.45
**8**	WT-Treated	32.41	5.73	17.67	28.18	2.79	10.46	28.14	2.36	9.26
**9**	WT-Treated	32.34	5.88	18.18	27.94	2.64	9.864	27.65	2.58	9.54
**10**	WT-Treated	31.95	5.78	18.09	27.65	2.76	9.761	28.43	2.63	9.74
**11**	WT-Treated	29.91	2.79	9.32	28.33	3.01	10.69	28.49	2.75	9.874
**12**	WT-Treated	29.88	2.82	9.43	24.75	2.49	8.64	27.34	2.67	9.648
**13**	WT-Treated	28.98	2.85	9.83	28.64	2.85	9.866	25.66	2.94	10.54
14	WT-Treated	24.62	2.09	9.24	26.49	2.68	8.743	25.76	2.86	10.25
15	WT-Treated	25.19	2.79	9.06	25.46	2.43	8.014	28.79	2.64	9.12
16	WT-Treated	27.60	3.46	9.86	24.86	2.96	10.96	27.41	2.79	9.46
17	WT-Treated	28.64	2.89	9.97	27.65	2.76	9.76	25.41	2.74	9.62

18	CRISPR-Treated	16.819	0.41	2.43	20.25	0.31	2.96	18.79	0.42	2.65
19	CRISPR-Treated	16.77	0.44	2.62	19.48	0.43	2.85	17.59	0.21	2.14
20	CRISPR-Treated	16.89	0.46	2.72	18.25	0.35	2.84	18.76	0.37	2.54
21	CRISPR-Treated	18.35	0.747	4.07	17.65	0.26	1.53	18.42	0.16	1.64
22	CRISPR-Treated	17.99	0.72	4.00	18.49	0.46	2.73	18.79	0.44	2.48
23	CRISPR-Treated	17.89	0.75	4.19	16.75	0.48	2.846	18.67	0.46	2.49
24	CRISPR-Treated	21.04	0.261	1.24	21.54	0.64	2.864	17.48	0.54	2.85
25	CRISPR-Treated	21.15	0.245	1.15	20.16	0.58	2.64	17.38	0.43	2.50
26	CRISPR-Treated	20.25	0.273	1.34	20.45	0.64	2.76	18.59	0.56	2.88
27	CRISPR-Treated	17.291	0.75	4.33	20.67	0.43	2.68	17.6	0.55	2.88
28	CRISPR-Treated	17.32	0.77	4.44	18.64	0.61	2.88	20.27	0.48	2.62
29	CRISPR-Treated	17.35	0.87	5.01	18.67	0.66	2.86	20.68	0.52	2.82
30	CRISPR-Treated	18.34	0.74	4.02	17.65	0.26	1.53	17.59	0.21	2.14
31	CRISPR-Treated	17.45	0.72	4.01	21.54	0.25	1.65	18.64	0.61	2.88
32	CRISPR-Treated	17.39	0.83	5.16	20.16	0.27	1.66	16.77	0.44	2.62
33	CRISPR-Treated	17.49	0.86	5.36	18.79	0.44	2.48	21.15	0.245	1.15

Table S9. Cell Death count raw data for *X. campestris* stress

**Sl. No.**	**Samples**	**Rep. 1**			**Rep. 2**			**Rep. 3**		
		**total leaf area**	**Diseased leaf area**	**percentage of disease damage**	**total leaf area**	**Diseased leaf area**	**percentage of disease damage**	**total leaf area**	**Diseased leaf area**	**percentage of disease damage**
**1**	Mock	48.9	0.7	1.43	50.12	0.2	0.52	50.45		
**2**	WT-Treated	48.87	0.72	1.44	48.4	0.42	1.21	25.41	2.74	9.62
**3**	WT-Treated	48.8	0.69	1.41	42.9	0.15	0.82	48.14	0.91	1.95
**4**	WT-Treated	45.9	0.86	1.87	46.2	1.14	1.16	35.66	0.95	1.93
**5**	WT-Treated	45.88	0.89	1.93	47.02	2.61	1.84	52.1	0.93	1.72
**6**	WT-Treated	46.86	0.9	1.92	44.59	0.10	1.14	48.8	0.85	1.60
**7**	WT-Treated	51.22	0.89	1.73	46.28	2.31	2.01	51.22	0.7	1.68

**8**	WT-Treated	52.1	0.9	1.72	47.63	3.16	2.46	48.9	0.76	1.69
**9**	WT-Treated	51.05	0.91	1.78	47.11	4.24	1.14	45.9	0.86	1.87
**10**	WT-Treated	50.55	0.95	1.87	42.08	2.16	1.56	48.9	0.7	1.43
**11**	WT-Treated	50.23	0.93	1.85	49.39	1.17	1.02	44.59	0.10	1.14
**12**	WT-Treated	51.22	0.94	1.83	41.28	2.10	2.03	51.05	0.91	1.78
**13**	WT-Treated	48.9	0.7	1.43	46.35	3.8	2.32	48.4	0.42	1.21
14	WT-Treated	45.9	0.76	1.56	48.94	2.65	2.15	25.41	2.74	9.62
15	WT-Treated	44.4	0.78	1.59	47.67	2.36	2.01	48.8	0.69	1.41
16	WT-Treated	48.8	0.85	1.60	47.49	2.48	2.15	46.86	0.9	1.92
17	WT-Treated	36.89	0.84	1.56	24.75	2.49	8.64	47.11	4.24	1.14
18	CRISPR-Treated	50.1	0.07	0.15	48.25	0.08	1.01	50.26	0.06	0.14
19	CRISPR-Treated	51.02	0.08	0.15	49.46	0.10	1.01	48.64	0.07	0.15
20	CRISPR-Treated	51.9	0.07	0.13	51.05	0.03	0.86	48.69	0.11	0.86
21	CRISPR-Treated	46.03	0.06	0.14	48.35	0.14	1.02	49.35	0.13	0.17
22	CRISPR-Treated	45	0.09	0.21	48.49	0.14	1.01	50.02	0.06	0.12
23	CRISPR-Treated	44.83	0.09	0.20	50.4	0.15	1.12	48.96	0.12	0.88
24	CRISPR-Treated	51	0.1	0.19	47.6	0.25	1.53	49.67	0.10	0.87
25	CRISPR-Treated	51.2	0.12	0.23	46.47	0.13	1.01	50.14	0.03	0.52
26	CRISPR-Treated	50.8	0.11	0.21	46.04	0.32	2.13	48.69	0.14	0.88
27	CRISPR-Treated	49	0.13	0.26	43.7	0.05	0.61	50.1	0.079	0.15
28	CRISPR-Treated	49.67	0.12	0.24	42.1	0.06	0.64	49.36	0.13	0.17
29	CRISPR-Treated	50.21	0.132	0.26	49.5	0.07	0.68	47.01	0.32	0.28
30	CRISPR-Treated	48.62	0.28	1.25	50.14	0.06	0.62	48.24	0.12	0.15
31	CRISPR-Treated	47.64	0.25	1.15	49.54	0.12	1.00	47.68	0.11	0.15
32	CRISPR-Treated	50.48	0.28	1.02	48.65	0.06	1.24	51.2	0.12	0.23
33	CRISPR-Treated	56.40	0.30	1.46	47.26	0.08	0.82	49.31	0.28	1.82

Table S10. Raw data for DAB staining assay of roots under *R. solanecearum* stress

Sl. No.	Samples	Percentage of accumulation of ROS in root		
		Rep. 1	Rep. 2	Rep. 3
1.	MOCK	6.88	7.85	8.25
2.	MOCK	5.63	6.31	8.62
3.	MOCK	7.89	5.26	5.34
4.	MOCK	5.66	4.25	5.02
5.	MOCK	7.67	5.01	5.62
6.	MOCK	5.57	4.06	5.71
7.	MOCK	5.90	5.04	5.86
8.	MOCK	5.45	5.06	6.24
9.	MOCK	5.72	6.86	6.35
10.	MOCK	8.42	7.42	6.27
11.	MOCK	5.70	8.65	6.34
12.	MOCK	5.81	7.64	6.78
13.	MOCK	4.25	5.20	5.37
14.	MOCK	5.06	5.32	5.03
15.	MOCK	5.18	4.31	7.61
16.	MOCK	6.34	4.18	7.00
17.	WT-Treated	26.13	24.16	33.16
18.	WT-Treated	25.66	28.05	33.15
19.	WT-Treated	29.25	28.14	36.14
20.	WT-Treated	22.53	26.24	26.31
21.	WT-Treated	30.41	29.14	25.00
22.	WT-Treated	35.54	27.00	27.19
23.	WT-Treated	36.85	26.01	28.61
24.	WT-Treated	21.77	25.64	24.18
25.	WT-Treated	25.84	22.34	25.31
26.	WT-Treated	49.24	22.01	28.15
27.	WT-Treated	43.39	30.56	22.64

28.	WT-Treated	25.54	30.65	23.67
29.	WT-Treated	30.82	34.79	23.02
30.	WT-Treated	35.20	34.15	24.32
31.	WT-Treated	25.62	34.25	26.31
32.	WT-Treated	28.64	35.62	24.63
33.	CRISPR-Treated	17.5	12.62	11.38
34.	CRISPR-Treated	14.74	12.37	15.62
35.	CRISPR-Treated	12.04	11.02	10.30
36.	CRISPR-Treated	10.18	9.152	10.25
37.	CRISPR-Treated	17.27	10.35	11.51
38.	CRISPR-Treated	16.70	10.00	12.05
39.	CRISPR-Treated	10.69	12.08	11.02
40.	CRISPR-Treated	10.58	13.04	10.35
41.	CRISPR-Treated	19.40	10.32	14.38
42.	CRISPR-Treated	10.02	10.11	14.02
43.	CRISPR-Treated	10.56	11.48	12.06
44.	CRISPR-Treated	12.01	13.15	10.45
45.	CRISPR-Treated	13.56	12.45	11.02
46.	CRISPR-Treated	10.35	12.48	12.63
47.	CRISPR-Treated	14.26	12.36	10.06
48.	CRISPR-Treated	15.32	12.14	12.65
